# Myopic versus perfect foresight target setting for Indonesia’s net zero electricity transition

**DOI:** 10.1016/j.isci.2025.112813

**Published:** 2025-06-02

**Authors:** Bintang Yuwono, Lukas Kranzl, Reinhard Haas, Ping Yowargana

**Affiliations:** 1 Energy Economics Group (EEG), Institute of Energy Systems and Electrical Drive, Technischen Universität Wien (TU Wien), Vienna 1040, Austria; 2 Biodiversity and Natural Resources Program (BNR), International Institute for Applied Systems Analysis (IIASA), Laxenburg 2361, Austria

**Keywords:** Energy resources, Energy sustainability, Energy Modeling

## Abstract

In the effort to align with Paris goals, decision-makers set targets that usually concern milestones earlier than 2100. These targets can be derived from different considerations of long-term implications of actions. This study investigates the implications of deciding on emissions targets based on myopic vis-à-vis perfect foresight using long-term energy system optimization model. The study reveals cost discrepancies correspond to the gaps between emissions derived from mixed integer linear programming (MILP) solution in perfect foresight scenarios versus exogenous values in myopic scenarios. When considering myopic approach, our study suggests that avoiding drastic emissions reduction can deliver minimum cost discrepancies relative to what can be achieved with perfect foresight. However, this poses a dilemma where less drastic emissions targets may risk increasing fossil power generation under lenient emissions reduction targets. Complementing less drastic emissions reduction targets with more ambitious policies promoting renewables is necessary to avoid the risk of increased reliance on fossil power generation.

## Highlights

• Myopic (MF) vis-à-vis perfect foresight (PF) implications toward energy system transition
• PF delivers earliest cost-effective actions; MF generates sub-optimal timing of actions
• MF biased toward mature technologies by focusing on the short-term requirements
• Early promotion of renewables can help avoid increased reliance on fossil power generation

## Graphical abstract

**Figure:**
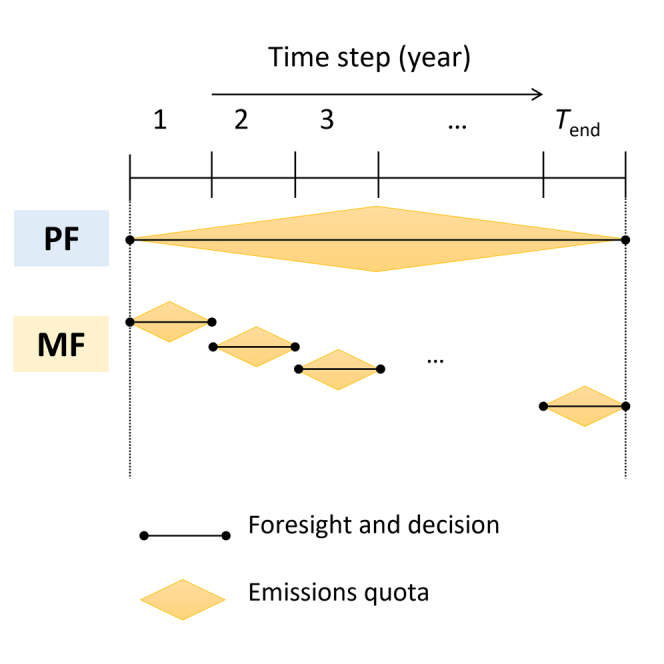

## Introduction

Achieving the goal of the Paris Agreement to limit global warming below 2°C by 2100 requires the implementation of greenhouse gases (GHGs) emissions reduction measures that are consistent with decarbonization pathways spanning over eight decades. Global transformation pathways to 1.5°C–2°C present a variety of emissions trajectories with different system-wide implications. These implications can be characterized by the timing and scale of emissions reduction measures—highlighting early improvement of energy efficiency and conservation measures, faster transition to renewable electricity, and utilization of carbon dioxide removal technologies.^1^ The long-term goal of these pathways is to keep global cumulative carbon emissions within a budget of about 420 GtCO_2_ from 2018 to 2100 for a good chance (>66%) of limiting warming to 1.5°C or about 580 GtCO_2_ for an even chance (50%).^2^ The pathways also present different timings in achieving net zero GHG emissions within the course of the 21^st^ century.

In the effort to align with Paris goals, decision-makers set strategies with targets that usually concern earlier milestones than 2100. These targets can take form in *inter alia* government policies or company commitments. At the two extremes, these shorter-term targets can either be (1) fully consistent with long-term trajectories that are in line with the global targets or (2) subject to contextual limitations at the time of target settings with limited information on its implication toward the full-length of the global target’s time horizon. The former resembles decision-making with perfect foresight, where decisions in the shorter terms consider full information about how the future may develop. In contrast, the latter resembles myopic decision-making, where decisions are made based on considerations of information that are relevant and available only for the short term and without considering the long-term implications.

Countries have set shorter-term targets to achieve net zero emissions within the remaining length of the 21^st^ century. These targets are formalized in national determined contributions (NDCs) and long-term low emissions development strategies (LT-LEDS) that are submitted to the United Nations Framework Convention on Climate Change (UNFCCC). The formulation of these targets differs among countries in terms of being based on a perfect foresight vis-à-vis myopic. Developed countries such as the European Union (EU),^3^^,^^4^ United Kingdom (UK),^5^^,^^6^ and United States of America (USA)^7^^,^^8^ have set net zero target year around 2050 or earlier based on alignment to global emissions pathways consistent with Paris goal. Despite not fully reflecting on a process that is based on perfect foresight, such an approach can still be considered a farsighted target setting as they are informed with long-term emissions trajectories that include the period beyond the target year. However, some developing countries, e.g., China,^9^^,^^10^ India,^11^^,^^12^ and Indonesia^13^^,^^14^ have set their net zero target year later than the developed countries based on the consideration of equity and common but differentiated responsibilities and capabilities principles of climate mitigation. In these examples, the selection of net zero target year is myopic in terms of alignment toward trajectories leading to the achievement of Paris goal in 2100, as they are motivated by differentiating climate change mitigation ambition between developed and developing countries. Similar deviation from using long term alignment Paris goal trajectories as target-setting consideration can also be found in companies’ net zero targets, e.g., Google,^15^ Samsung,^16^ and Amazon^17^ which are set based on internal aspiration of how their businesses should develop in the context of changing climate and business environment.

Indonesia have laid the vision to achieve net-zero emissions target in 2060 with concrete plans for transforming major emitting sectors of the economy.^13^^,^^14^ Indonesia’s energy sector holds the largest potential emissions reduction, including from the decarbonization of the electricity sector. Among other initiative to support this plan, Government of Indonesia and other partnering countries have jointly declared the Just Energy Transition Partnership (JETP), aiming at accelerating the achievement of Indonesia’s net zero electricity emissions by 2050.^18^ Furthermore, Indonesia has come up with concrete action plans laid out in the Comprehensive Investment Policy Plan in expanding the transmission grid infrastructure, deploying renewables capacity, phasing-out of coal power, and improving energy efficiency.^19^ Previous studies on power system net zero transition pathways have also suggested similar technology deployment strategies.^20^^,^^21^ However, existing policies and their substantiating action plans do not concern implications after targeted net zero year. This can be problematic especially considering Indonesia’s growing electricity demand which poses a unique challenge in addition to achieving Paris emissions reduction goal by 2100. In long-term planning context, early investment decision of strategic assets such as extending large-capacity transmission network or deploying large-scale power generation requires the anticipation of future demand. More specifically, early phase out of fossil power generation can either be more costly than anticipated or even reversed when future needs to fulfill new demand becomes urgent and apparent.

While other studies have examined the implications of myopic and perfect foresight decision-making in long-term energy system planning,^22^^,^^23^^,^^24^^,^^25^ knowledge gaps remain on the implication of using different decision-making foresights in setting emissions reduction targets. This study aims to fill this gap by investigating the implications of myopic versus perfect foresight target setting against the backdrop of achieving the Paris goal by the end of this century. Using energy system optimization model, we evaluate the implications of deciding on emissions targets based on myopic vis-à-vis perfect foresight toward the resulting emissions trajectory, costs and investment requirements, and technology configurations. We also investigate the implications under varying net zero ambitions. Our investigation is conducted using the specific case study of Indonesia’s net zero electricity transition. Such a selection provides context for evaluating specific features of the energy system mentioned previously while still providing insights that can be extrapolated to other energy systems. Finally, we present and discuss the results with the intention of deriving policy insights and providing recommendations for decision makers.

## Results

### Costs and investment requirements

Assessing implications of myopic versus perfect foresight target setting requires the exclusion of the implication of net zero transition requirement. This is important as net zero transition will already generate significant changes compared to the baseline scenario (BL), which might obscure the implications of myopic versus perfect foresight target setting. Therefore, comparison of results related to costs and investment (also for power generation, transmission, feedstock fuel, and CO_2_ transport in the following sections) only considers the additional requirements of net zero scenarios compared to BL. All cost values are expressed in US$ based on 2020 constant value.

**Figure 1:**
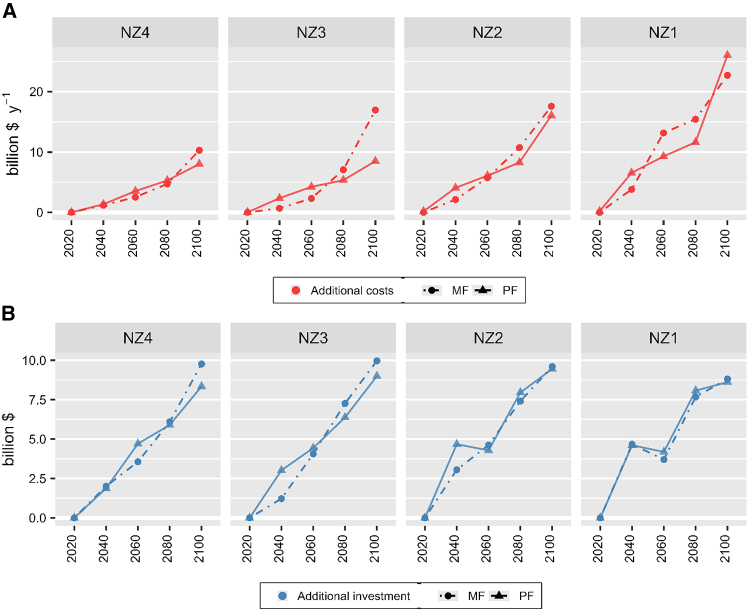
Total system costs and investment trajectories (A) Additional total system costs requirements (red). (B) Additional investments requirements (blue). The additional total system costs and investment requirements (billion US$ of 2020 constant values) of net zero ambitions (NZ1–NZ4) based on myopic (MF, dots with dashed lines) and perfect foresight (PF, triangles with solid lines) scenarios are compared to the baseline scenario (BL) from 2020 to 2100. Total system costs comprise of annualized costs of investment, operation and maintenance, and fuel. Total investment is defined as lump-sum capital expenditure required for deploying technology capacity for the length of timestep’s interval.

Across all net zero ambitions (NZ1–NZ4), myopic scenarios require higher additional costs than perfect foresight scenarios (Figure 1A). Although there are periods with lower additional system costs, myopic scenarios result in 2.7%–32% (0.5–6.6 billion $) higher cumulative additional costs in 2020–2100. Note that the gap in additional costs is most significant in NZ3 (32%) as compared to other net zero ambitions (NZ1 = 2.7%, NZ2 = 4.4%, and NZ4 = 2.9%). Note that additional costs of myopic and perfect foresight scenarios for all net zero ambitions correspond to approximately $249 billion of cumulative total system costs in BL. For more information on net zero ambition emission targets (NZ1-NZ4) can refer to method details.

Myopic scenarios lead to the postponement of investments needs to later periods, especially in lower net zero ambitions. In terms of cumulative additional investment throughout 2020–2100, myopic scenarios result in 2.4%, 6.4%, and 1.4% ($12 billion, $33 billion, and $6 billion) less requirement for NZ1, NZ2, and NZ3, respectively. However, myopic scenario results in 2.9% ($12 billion) more cumulative additional investment requirements in NZ4. Note that additional investments for all scenarios correspond to approximately $542 billion of cumulative investment in the baseline scenario.

### Emissions trajectories

**Figure 2:**
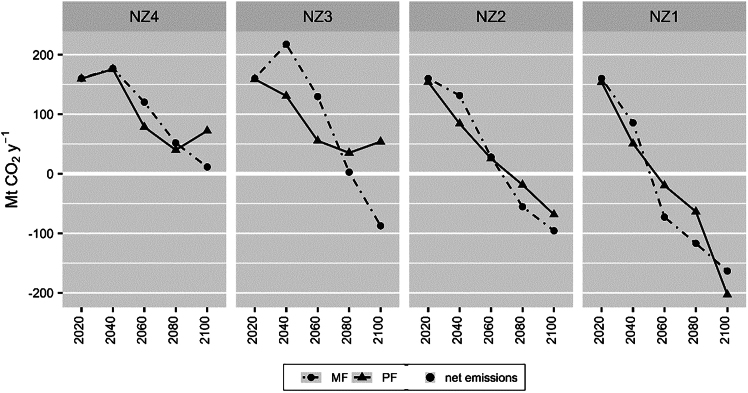
Annual net emissions trajectories The annual net emissions (MtCO_2_ y^−1^) trajectories of the different net zero ambitions (NZ1–NZ4) based on myopic (MF, dots with dashed lines) and perfect foresight (PF, triangles with solid lines) scenarios from 2020 to 2100. Net CO_2_ emissions comprise of gross CO_2_ emissions subtracted by CO_2_ emissions from bioenergy and CO_2_ captured. All captured CO_2_ is assumed to be injected and sequestrated.

Across all net zero ambitions (NZ1–NZ4), myopic scenarios result in pathways with higher emissions in earlier periods but turn lower in later periods compared to perfect foresight scenarios (see Figure 2). The most significant effect can be observed in NZ3, where myopic scenario results in 67% and 134% (87 and 74 MtCO_2_) higher emissions than perfect foresight scenario in 2040 and 2060 but then leads to 92% and 261% (32 and 141 MtCO_2_) lower in 2080 and 2100. This implies delayed actions to reduce emissions that are present until a certain point in time in the future, i.e., 2087 (NZ4), 2064 (NZ3), 2069 (NZ2), and 2042 (NZ1). From that point onwards, myopic scenarios lead to more emissions reduction, thus lower net emissions compared to perfect foresight scenarios. Scenarios that concern the highest level of ambition display a more complicated trajectory. While the periods of lower emissions as delayed climate mitigation action remain until 2100 in NZ2-NZ4, the corresponding lower emissions period under NZ1 remains only until 2081. In the following timestep (2100), perfect foresight scenario results in 24% (40 MtCO_2_) lower emissions than myopic scenario. Early emissions reduction in perfect foresight scenarios also corresponds to later achievement of net zero emissions (NZ1 and NZ2) or no requirement at all (NZ3 and NZ4) compared to myopic scenarios, despite both scenario sets meeting the same cumulative emissions quota constraint.

### Power generation and transmission

**Figure 3:**
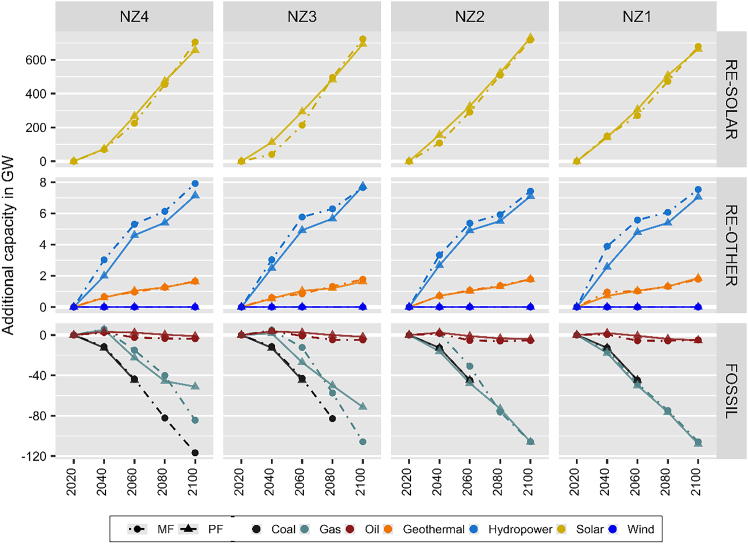
Additional electricity generation capacity requirements The additional electricity generation capacity requirements (GW) of net zero ambitions (NZ1–NZ4) based on myopic (MF, dots with dashed lines) and perfect foresight (PF, triangles with solid lines) scenarios are compared to baseline scenario (BL) from 2020 to 2100. Note that bioenergy presented separately in Figure 5A and other technologies considered in the model that are not selected by the optimization are not shown (i.e., Cofire, Cofire+CCS, Coal+CCS, Gas+CCS, and Nuclear).

Compared to perfect foresight scenarios, almost all myopic scenarios result in smaller deployment of renewables in early periods followed with larger deployment in later periods. This is mainly caused by solar power trajectories (Figure 3A), which represent most (75–97%) of the total renewable capacities in 2040–2100. The most significant effect can be observed in NZ3 trajectories, where myopic scenario results in 64% and 27% (72 and 80 GW) smaller additional solar power generation capacities in 2040 and 2060, followed by 3% and 4% (12 and 30 GW) larger in 2080 and 2100 compared to perfect foresight scenario. An exception to aforementioned pattern occurs in NZ2 where myopic scenario results in 2%–31% (13–48 GW) smaller additional solar power generation capacity than perfect foresight scenarios in all timesteps. Note that in BL, there is 0.3–54 GW of installed solar power generation capacities in 2020–2100.

The pattern for renewables deployment does not apply for specific renewable power generation other than solar power (see Figure 3A). Myopic and perfect foresight scenarios of all net zero ambitions (NZ1–NZ4) yield identical power generation capacity, also compared to BL, for wind power (5–154 MW in 2020–2100). For hydro power, myopic scenarios result in 4.5%–52% (0.3–1.3 GW) larger additional capacities than perfect foresight scenarios across all net zero ambitions (NZ1–NZ4) in 2040–2100. For geothermal power, myopic scenarios result in 0.4%–34% (5–245 MW) larger additional capacities across all net zero ambitions for the majority of timesteps, except for 2060 and 2100 (0.6%–18% or 10–190 MW smaller deployment).

As less renewables were being deployed in earlier periods, myopic scenarios correspond to delayed phase out of fossil power generation (see Figure 3A). However, the effect is not the same for all types of fossil fuel as well as different levels of net zero ambitions. Perfect foresight scenarios of all net zero ambitions result in complete phase out of coal after 2060. Similar trajectories can be found under myopic scenarios for higher net zero ambitions (NZ1 and NZ2). However, myopic scenarios for lower net zero ambitions demonstrate a delayed phase out after 2080 (NZ3) while still leaving 1.2 GW of coal power in operation until 2100 (NZ4). For gas power generation, myopic scenarios result in larger deployment of additional capacities in earlier periods, followed with smaller deployment in later periods. The most significant effect can be observed in NZ3, where myopic scenario results in 206% and 55% (3 and 15 GW) larger additional gas power generation capacities in 2040 and 2060, followed by 15% and 48% (7.5 and 34 GW) smaller in 2080 and 2100. However, gaps between myopic and perfect foresight scenarios become smaller toward higher net zero ambitions. For oil power generation, myopic scenarios result in smaller capacities in 2040–2100 across all net zero ambitions (NZ1–NZ4).

**Figure 4:**
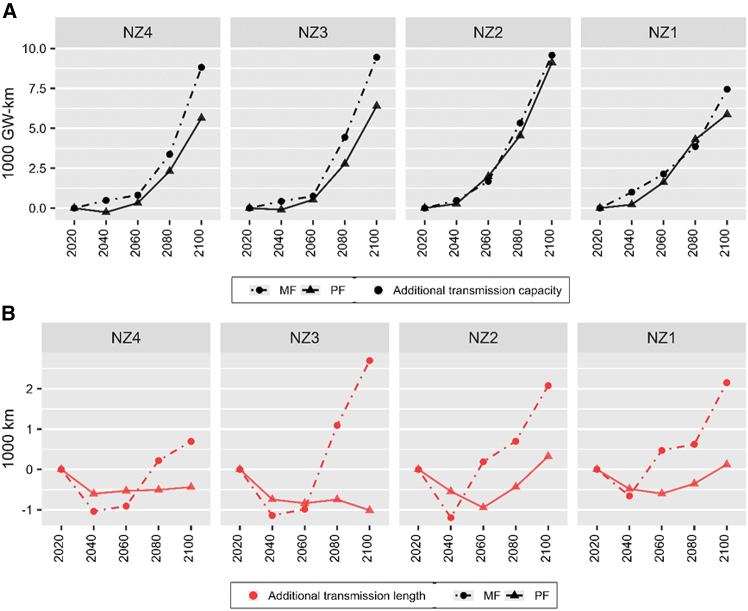
Additional transmission infrastructure requirements (A) Additional electricity transmission capacity requirements (in 1,000 GW km, black). (B) Additional transmission length requirements (in 1,000 km, red). Additional electricity transmission capacity and length requirements of net zero ambitions (NZ1–NZ4) based on myopic (MF, dots with dashed lines) and perfect foresight (PF, triangles with solid lines) scenarios are compared to baseline scenario (BL) from 2020 to 2100.

Myopic scenarios also result in 5%–530% (207-3,175 GW-km) larger deployment of additional transmission capacities in most of the timesteps of all net zero ambitions (see Figure 4A). Significant additional deployment is apparent in lower net zero ambition scenarios (NZ3 and NZ4) but not in higher net zero ambitions (NZ1 and NZ2). In terms of length of transmission network, myopic scenarios result in 258-1,652% (1,134-3,713 km) more additional expansion in 2100 for all net zero ambitions (NZ1–NZ4) (see Figure 4B). This is the aggregate result of 36%–120% (148–651 km) less additional expansion in earlier periods, followed with 120%–1,652% (727-3,712 km) more additional expansion in later periods. Note that the additional expansion of transmission capacities and length for all scenarios of net zero ambitions correspond to approximately 2,000–14,000 GW-km of transmission capacity and 35,000–89,000 km of transmission length in 2020–2100 under BL.

### Bioenergy, CCS, and BECCS

**Figure 5:**
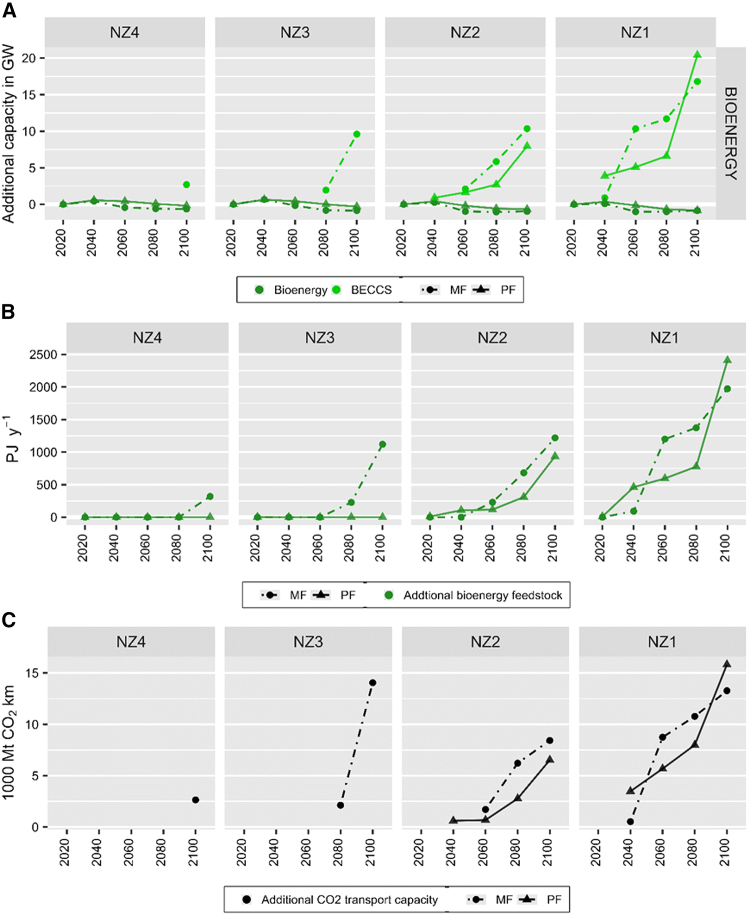
Additional requirements of BECCS and CO_2_ transport (A) Additional requirements of bioenergy electricity generation capacity (GW). (B) Additional requirements of biomass feedstock for power generation (in PJ y^−1^, green). (C) Additional requirements of CO_2_ transport capacity (in MtCO_2_ y^−1^ km, black). Additional requirements of bioenergy generation capacity, biomass feedstock, and CO_2_ transport capacity of net zero ambitions (NZ1–NZ4) based on myopic (MF, dots with dashed lines) and perfect foresight (PF, triangles with solid lines) scenarios are compared to baseline scenario (BL) from 2020 to 2100. Biomass feedstock comprises of solid biomass, bioethanol, and biodiesel, excluding biogas.

Deployment of additional bioenergy without CCS is minor in all scenarios throughout the modeling horizon. For bioenergy coupled with CCS (BECCS), myopic scenarios result in increased deployment in later timesteps (see Figure 5A). In lower net zero ambitions, myopic scenarios result in additional BECCS power generation capacities of 2 and 10 GW in 2080 and 2100 (NZ3) and 3 GW in 2100 (NZ4) while perfect foresight scenarios lead to no deployment throughout 2020–2100 (NZ3 and NZ4). In NZ2, myopic scenario results in 2 GW of BECCS being deployed in 2060, instead of 1 GW BECCS deployment in 2040 in the corresponding perfect foresight scenario.

Similar to other aspects previously presented, NZ1 result demonstrate multiple overlaps between myopic and perfect foresight scenarios’ trajectories. Compared to perfect foresight, myopic scenario generates smaller deployment of BECCS in 2040, followed with larger deployment in 2060–2080, before going back to deploying less BECCS in 2100. As seen in Figure 5A, this is due to the majority (56%) of BECCS deployment in myopic scenario taking place in 2060, while the majority (68%) of BECCS deployment in perfect foresight scenario takes place in 2100. Overall, myopic scenarios result in 10% (4 GW) more cumulative additional BECCS capacities being deployed throughout 2020–2100.

More BECCS deployment also means that myopic scenarios result in higher requirements of biomass feedstock (see Figure 5B) and CO_2_ transport capacity (see Figure 5C) than perfect foresight scenarios. This is most visible in lower net zero ambitions (NZ3 and NZ4), where myopic scenarios result in 1,119 and 318 PJ of additional biomass requirements in 2100, while perfect foresight scenarios require 0.4 PJ less to 0.74 PJ more than BL. In addition, myopic scenarios result in deployment of about 14,000 and 2,600 MtCO_2_ y^−1^ km of CO_2_ transport capacity in 2100 under NZ3 and NZ4 while perfect foresight leads to zero deployment under both NZ3 and NZ4. Meanwhile, in higher net zero ambitions (NZ1 and NZ2), myopic scenarios result in smaller biomass feedstock in early periods and then followed with larger requirements, corresponding to the increasing BECCS capacities. In addition, the delayed roll-out of BECCS also corresponds to the delayed expansion of CO_2_ transport infrastructure. Note that CCS technologies are only selected for BECCS application. Although options of fossil-fired CCS and co-firing biomass and coal with CCS are available, the optimization results suggest that they are less cost-competitive than the combination of renewables and BECCS deployments in achieving emissions reduction.

## Discussion

Our depiction of myopic target setting in myopic scenarios is the equivalent of substituting a decision variable (i.e., emissions) in perfect foresight scenarios with exogenous pre-defined values. It is, therefore, conceivable that the exogenous emissions values are also within the solution space of the mixed integer linear programming (MILP) problem depicting perfect foresight target setting which generates the study’s perfect foresight scenarios. As the MILP formulation utilizes cost minimization as an objective function, it is anticipated that perfect foresight scenarios should deliver lower total system costs compared to myopic scenarios.

This study provides quantitative insight on the cost “savings” that are generated by perfect foresight target setting vis-à-vis myopic approach. Perfect foresight scenarios result in 2.9%–24% (107-1,314 million $ per year) lower additional costs throughout 2020–2100, which correspond to 0.2%–2.4% of the total system costs (54–55 billion $ per year). Lower total system costs in perfect foresight scenarios have the trade-off of 1.1%–6.5% (51–319 million $ per year) higher additional investment or 0.03%–0.15% of total investment requirement (200–214 billion $ per year). This translates to about $2–4 costs saved per $1 increase in additional investment when comparing perfect foresight vis-à-vis myopic scenarios. The cost and investment requirement discrepancy between the two approaches are indeed insignificant in relative terms, but the magnitude of their absolute amount is useful in evaluating the necessity or advantages of either target setting approach.

In evaluating the implication of myopic and perfect foresight target setting across different levels of net zero ambitions, the study reveals no salient pattern that links the extent of cost discrepancy resulting from the two approaches with the varying level of ambitions. Instead, the cost discrepancies correspond to the gaps between emissions derived from MILP solution in perfect foresight scenarios versus exogenous values in myopic scenarios. Clearly, these gaps cannot be anticipated in advance since there will likely be just one approach taking place in a real-life decision-making environment. However, the different levels of ambitions represent different emissions trajectories with varying degrees of changes in annual emissions levels. From this perspective, the study reveals that the largest gap in cost-efficiency corresponds to exogenous targets with the “steepest” changes in emission levels over time (NZ3). This finding suggests that the myopic approach can be more costly when the selected targets imply drastic changes in emission levels, presumably resulting in distinct periods of high emission allowance vis-à-vis low or negative emission allowance.

Perfect foresight approach results in targets that are based on perfectly timed cost-effective actions after considering all potential technology configurations and future requirements for the entire planning horizon. In this study, the exogenous assumption in myopic approach results in delayed actions compared to the perfect foresight scenarios results. However, in other cases, myopic targets can also result in early actions. Using insights from MILP optimization, regardless of earlier or delayed actions as result, myopic emissions targets will always generate higher total system costs. Therefore, other than avoiding “steep” emissions reduction as previously indicated, myopic target setting should also consider the importance and consequences of early or delayed net zero transition.

Our study reveals significant implications of myopic vis-à-vis perfect foresight approach on technology configurations. Due to the shorter time horizon, myopic approach identifies least-cost options that are sufficient only for the requirements in the shorter-term and disregards their potential long-term implications. This approach offers more competitive advantage for mature technologies that are currently more affordable.^22^^,^^23^^,^^24^^,^^26^ At the same time, myopic approach presents disadvantages for less mature technologies that are currently more expensive but have significant potential to become more affordable in the future compared to mature technologies. Consequently, myopic approach results in sub-optimal timing of actions that may lead to considerable change of present and future technology requirements. This phenomenon is evident in our study results, particularly in observing the deployment of fossil power generation. Myopic scenarios result in deployment of additional coal power, which not only leads to delayed fossil phase-out but also future deployment of BECCS due to carbon dioxide removal requirements. This pattern is only evident in myopic scenarios that allow more space for increasing emission (NZ3 and NZ4). In scenarios that require immediate emission reductions (NZ1 and NZ2), additional coal power generation is not selected leading to complete phase-out in 2060.

In real-life decision-making context, perfect foresight is unattainable and all information about the future remains uncertain to varying degrees. This is also the case for a more specific “perfect foresight” approach depicted in this study, which adopts cost minimization in achieving long-term emissions goal as the main logic to generate optimal decisions. In reality, other factors can also influence, or even dominate, the consideration of climate targets. This can be found in previously presented examples where countries’ net zero targets are set based on their perceived perception of fairness in global climate mitigation efforts. Additionally, companies’ net-zero targets are driven by business motives, aiming to gain competitive advantage by means of retaining positive stakeholder perception and overcoming trade barriers. Although these are valid and common considerations in determining climate action targets, we consider these examples as “myopic” target-setting as they do not consider the long-term implications toward achieving the Paris goal. Considering the pervasiveness of “myopic” target setting, one of the primary objectives of this study is to provide recommendations that would allow such a target setting to result in minimum costs discrepancy compared to targets informed by implications toward achieving long-term goal. To this regard, the bias of myopic approach toward mature technologies poses an interesting dilemma. As demonstrated from our results, such a bias risks increasing reliance on coal power—leading to more costly future reduction requirements—under the presence of lenient emissions targets. On the other hand, setting excessively “steep” reduction targets risks increasing cost discrepancy relative to targets that are informed by optimal timing of actions in achieving long-term goal. The dilemma appears when more detailed policies (e.g., energy mix targets) are derived as a consequence of emission reduction targets. In addressing this dilemma, it is important to not rely on emissions reduction targets alone to influence sectoral policies for new technology investments. Instead, less ambitious emission reduction targets can be coupled with more ambitious policies in promoting renewables as the latter could also contribute to positive impacts beyond climate change mitigation (e.g., industrial competitiveness, resource conservation, energy security, etc.)

Implications of myopic vis-à-vis perfect foresight net zero target setting on technology configurations also has consequences on transmission requirements. Myopic scenarios result in increasing geographical expansion of transmission lines by 258-1,652% longer than perfect foresight scenarios. This leads to significant implementation complexity and may risk adversely affecting land uses and land cover change.^27^ Developing longer transmission grid network requires more time and resource while including a wider range of stakeholders and jurisdictions in securing right-of-way and constructing infrastructures.^28^ Due to Indonesia’s vast forest area, significant geographical expansion of transmission network could risk going through forests and protected areas leading to loss in habitat and biodiversity.^29^^,^^30^ Furthermore, clearing of corridors through forest areas may increase access for forest encroachment and conversion to agricultural lands, leading to significant losses in terrestrial carbon stock.^31^^,^^32^^,^^33^ These issues reflect another dimension of decision-making complexity beyond cost minimization. This insight provides further emphasis to previous caution on the importance of coupling myopic emissions reduction targets with specific relevant policies. These policies can range from closely linked net zero technology policies (e.g., renewable energy portfolio or fossil power phase out) to more loosely related policies such as strengthening land use governance especially in areas that are impacted by net zero infrastructure development.

### Study limitations and future developments

In our study, we investigated the implications of myopic vis-à-vis perfect foresight target setting for net zero emissions electricity transition using ESOM framework. As explained in the methodology section, ESOM generates normative pathways of specific scenario depiction. While this is useful in evaluating policies for energy system interventions, such an evaluation is based on single normative result which may obscure the full spectrum of possible alternatives. This is a common problem in all ESOM-based analysis,^34^ which is also inherited in our study.

Our study investigates the implications of myopic approach using specific exogenous targets based on four different scenarios of emissions allowances (NZ1–NZ4). NZ1–NZ4 represent a wider range of national emissions allocations scenarios derived from “burden sharing” frameworks in allocating national allowances from the global emissions scenarios of RCP2.6 (2°C) and RCP1.9 (1.5°C).^35^ Additionally, NZ1 and NZ2 myopic targets fall within the range of existing national net zero year targets around 2050 and 2060^13^^,^^14^ while NZ3 and NZ4 exhibit scenarios with lower levels of ambitions. By setting a limited set of scenarios, the investigation leaves out all other possible myopic target setting scenarios, such as other emissions pathways that are relevant to other warming levels, possibilities of temperature overshoots, or other descriptions of plausible future events. However, we consider that the selection of myopic emissions targets is sufficient, considering the relevance to global 1.5°C–2°C warming targets and alignment with existing national net zero targets. Additionally, the varying trajectories of myopic emissions targets are adequate in representing varying “steepness” and intensity of emissions reduction requirements. Future research development could explore a larger set of scenarios depicting more varying emissions trajectories, to help expand the range of insights on implications of myopic vis-à-vis perfect foresight approaches.

SELARU model implemented for this study considers no elasticities. As all input parameters are pre-determined, changes of decision variables in response to results from previous timesteps are not considered in the model. In this study, we apply exogenous input for demand projections. Additionally, future costs of technologies and prices of feedstock fuels are also exogenously introduced with no technology learning effect considered in the model. This setup is deliberate to isolate the impact of elasticity assumptions from the impacts of myopic vis-à-vis perfect foresight target setting. We expect that the effects of both demand response and endogenous technology learning effect will significantly influence future energy system configurations,^36^^,^^37^ obscuring the impacts of myopic vis-à-vis perfect foresight target setting. For instance, changing the decision to deploy certain technologies as new information was fed into the optimization model due to the cumulative change effect—which is not captured in a perfect foresight. Future research development could include demand response and endogenous technology learning to evaluate their additional implications to complement the results of this study.

In a real-life decision-making environment, uncertainties persist due to various factors such as technological advancements, market dynamics, and policy changes. However, our study utilizes a deterministic approach in which all input parameters (e.g., future power plant costs and efficiencies, demand projections, and emissions targets) are estimated using the best available data. Future research development could address uncertainties in a more robust manner by using, stochastic approaches.^38^^,^^39^^,^^40^

## Resource availability

### Lead contact

Further information and requests for resources and reagents should be directed to and will be fulfilled by the lead contact, Bintang Yuwono (yuwono@iiasa.ac.at).

### Materials availability

This study did not generate new unique reagents.

### Data and code availability

• All datasets that are used for the calculation are available from the cited references. Compilation of these datasets has been deposited in https://doi.org/10.5281/zenodo.15221073 and is publicly available as of the date of publication.
• Complete results of model runs can be reproduced using datasets and GAMS codes that have been deposited in https://doi.org/10.5281/zenodo.15221073 and are publicly available as of the date of publication.
• Summary of input data and calculation results can be found in the supplemental information.

## Acknowledgments

This work was supported by the RESTORE+ project (www.restoreplus.org), which is part of the International Climate Initiative (IKI), supported by the 10.13039/501100006549Federal Ministry for the Environment, Nature Conservation and Nuclear Safety (BMU) based on a decision adopted by the German Bundestag.

## Author contributions

B.Y. and P.Y. conceived the idea and developed the concept for the study. B.Y. performed input data collection and processing. B.Y. performed computer model formalization and execution of model-based investigation. B.Y. performed visualization and data presentation. B.Y. and P.Y. led the writing and analysis. All authors discussed the idea and contributed to the manuscript. P.Y. supervised the study.

## Declaration of interests

The authors declare no competing interests.

## STAR★Methods

### Key resources table

**Table:** 

REAGENT or RESOURCE	SOURCE	IDENTIFIER
**Software and algorithms**		

R Studio (Version 2023.09.0)	The R Project for Statistical Computing	https://cran.r-project.org/
QGIS (Version 3.36.2-Maidenhead)	QGIS Project (OSGeo)	https://www.qgis.org
GAMS (Version 36.2.0)	The General Algebraic Modeling Language	https://www.gams.com/
SELARU (Version 1.2)	Spatially explicit Energy and LAnd system InfrastRUcture (SELARU) modeling framework	https://doi.org/10.5281/zenodo.15221073

### Method details

#### SELARU modeling framework

Energy system optimization models (ESOMs) are based on accounting framework of various aspects of the energy system such as resource availability, technology options, costs, and prices. As they typically utilize linear programming approach, ESOM generates optimum energy system configuration through solving decision variables to achieve the objective function of cost minimization while adhering to pre-defined system constraints. Hence, such an approach produces, ideal, normative pathway of specific scenario depiction resulting in the widespread use of ESOM in policy evaluation for energy system interventions.^34^ We use SELARU, a long-term energy system optimization model that has been previously applied in the investigation of Indonesia’s electricity sector^41^ to explore various scenarios depicting net zero transition based on both myopic and perfect foresight target settings. As the same input parameters are used in all scenarios, we can isolate and assess the system-wide impacts (i.e., timing and scale of emission reduction, capacity deployment, resource use, costs, and investment requirements) of both target-setting approaches.

SELARU modeling framework covers a wide range of technological options in electricity generation, transmission, and carbon capture, transport, and storage (CCS). The optimization is formulated using mixed integer linear programming (MILP) generating required capacity deployment and utilization of the different technologies to minimize system-wide cost while meeting a pre-defined electricity demand and various technological and physical constraints such as resource availability, environmental restrictions, and policy constraints. The objective of this study is not to assess short- or medium-term projections but rather generating long-term strategies and insights, until the end of century. We set up SELARU to balance energy supply and demand in annual resolution and investments rounds in 20-year interval timesteps from 2020 to 2100. We consider that the 20-year interval is sufficient in capturing the dynamics of investment strategies throughout the lifetime of technologies. The lifespan of technology options considered in this study is between 20 and 100 years and were obtained from national^42^ and international sources.^43^^,^^44^^,^^45^^,^^46^^,^^47^ SELARU incorporates spatially explicit information on *inter alia* resource availability and demand location to allow the potential configurations of large-scale centralized vis-à-vis small-scale distributed electricity systems. Such a representation is conducted for 500 regions with 1700 possible connections between the regions. This is particularly important for Indonesia considering the geographical complexity and the need for expanding transmissions network in an archipelagic, developing economy context. A more detailed explanation of the modeling framework can be found in Methods S1. SELARU—MILP Formulation.

#### Baseline scenario

We establish a baseline scenario (BL), comprising the main conditions of electricity demand fulfillment without considering any climate change mitigation goals. The resulting pathway under BL considers complete information on future developments and is solved simultaneously for the entire planning horizon. Exogenous demand scenario is obtained from national demand projection that is downscaled to model spatial resolution based on national and sub-national historical electricity consumption,^48^ population,^49^ gross domestic product,^50^ and national population projection from the Shared Socioeconomic Pathways “Middle of the road” scenario (SSP2).^51^ Renewable resource potential and geographical distribution are obtained from ESDM One Map,^52^ World Bank Indonesia Hydropower Study,^53^ Global Solar Atlas,^54^ and Global Wind Atlas.^55^ Note that the modeled resource technical potentials are factored by the size of available land that excludes protected areas, settlements, steep incline areas, and waterbody. Resource potential and infrastructure development costs are different from one location to another based on considerations of terrain conditions, existing logistic infrastructures, and regional level and pace of development.

The description of costs and technical performance of different energy technologies are obtained from national^42^ and international sources.^43^^,^^44^^,^^45^^,^^46^^,^^47^ Due to shortcomings of literature, changes of investment costs are considered only up to 2050 and followed with no changes until 2100 (end of planning horizon). Fuel specifications and prices are based on 2020 national values obtained from multiple sources^48^^,^^56^ without considering any changes in future periods. Existing capacities of stock electricity generation facilities, transmission lines, and transformer substations are obtained from the ESDM One Map.^52^ Planned infrastructures are considered based on the information from Electricity Supply Business Plan of the State-Owned Electricity Company (RUPTL-PLN 2021–2030).^57^ Complete information and dataset on model input parameters can be accessed on the study’s public repository (https://doi.org/10.5281/zenodo.15221073). A summary of input data can be found in Document S1. Figures S1–S3 and Tables S1–S16.

#### Net zero ambitions

National alignment to the Paris goal requires disaggregation of emissions pathways from global to national levels. However, such a “burden sharing” exercise can be challenging as it requires normative decisions to manifest equitability principles^58^ that will in turn decide the mathematical formalization of the international emissions allocation mechanisms.^35^^,^^59^ Resulting national allocation from a single global emissions pathway can have varying emissions trajectories and cumulative quotas when different equitability principles are applied. Here we consider 416 emissions pathways for Indonesia^35^ based on 13 allocation mechanisms that represents diverse applications of equitability principles over 32 different scenarios covering a wide array of long-term uncertainties. These uncertainties are reflected in multiple emissions pathways based on multiple shared socio-economic pathways (SSPs) under different representative concentration pathways that correspond to 1.5°C–2°C warming (RCP1.9 and RCP2.6) from six different Integrated Assessment Models (IAMs). The national emissions scenarios are then sorted based on the earliest year of net zero emissions before grouped into four clusters, i.e., NZ1-NZ4. NZ1 represents the top 25% of ambitious climate scenarios with net zero emissions reached around 2050; NZ2 around 2070; NZ3 around 2080; and NZ4 (bottom 25%) which does not require getting to net zero emissions until 2100.

The average value of national emissions from the different scenarios that fall into NZ1-NZ4 are then downscaled to electricity sector (see Figure 1) considering projections of sectoral emissions and final energy mix up to 2050 in accordance to the assumptions used in Indonesia LT-LEDS.^14^ Similar to literature limitation in baseline scenario assumptions, we assume no changes in sectoral projection in the following period of 2050–2100. This exercise resulted in emissions trajectories for the four levels of net zero ambitions (i.e., NZ1-NZ4) which provide annual emissions values resulting in cumulative emissions of −81, 177, 435, and 527 MtCO_2_ from 2020 to 2100 in 20-year intervals.

#### Perfect foresight versus myopic target settings

Similar to other ESOMs, SELARU uses linear programming to solve decision variables with the objective function of cost minimization for the modeled time horizon. Within this time horizon, generated solutions for all decision variables are fully informed by the complete set of input parameters. In the context of long-term scenario analysis, this process already resembles decision-making that is based on perfect foresight. Therefore, in exploring net zero electricity transition scenarios that depict perfect foresight target setting, we implement the SELARU model to endogenously determine emissions values for all 20-year interval timesteps in 2020–2100. To investigate varying net zero ambition scenarios, we apply cumulative emissions for 2020–2100 from NZ1-NZ4 as constraints to ensure that the resulting emissions values—i.e., emissions targets for each timestep—will deliver national disaggregation of the Paris goal.

**Figure:**
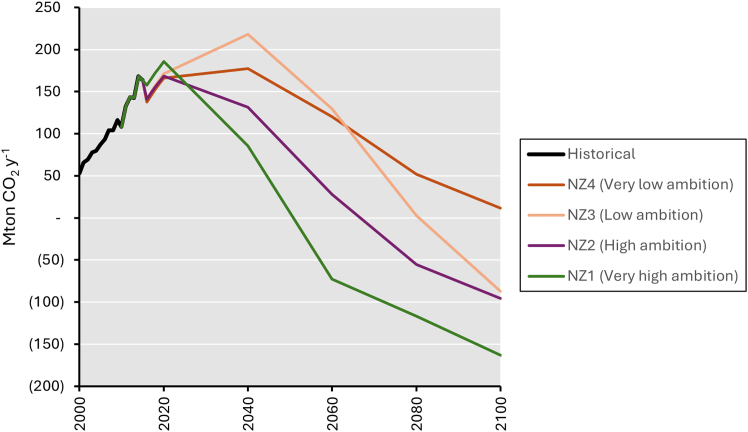
Emissions scenarios of Indonesia’s electricity sector Long-term emissions scenarios (in Mt CO_2_ y^−1^) for Indonesia’s electricity sector are presented under various climate ambitions (NZ1–NZ4). Historical emissions are sourced from Indonesia’s Biennial Update Report (BUR).

A different model treatment is required to generate net zero electricity transition scenarios that depict myopic target setting. For this, we shorten the model time horizon to 20 years, provide exogenous emissions quota for every timestep, and recursively run the model for the following timesteps throughout 2020–2100. In short, depiction of myopic net zero electricity transition scenarios is achieved by separating a single long-term optimization problem into a set of shorter-terms problems. The exogenous emissions quota in each timestep are based on yearly disaggregation values of NZ1-NZ46. This application will result in the same cumulative emissions values between myopic and perfect foresight scenarios to ensure commensurability. It is also possible to arbitrarily set the yearly emissions quota as long as they meet the constraint of generating the cumulative emissions value. However, the exogenous values we introduce represent national disaggregation for net zero ambition^35^ which happen to also yield comparable net zero year ambitions in national commitment and aspirations. Ultimately, we have two sets of net zero electricity transition scenarios, each set depicting myopic (MF) and perfect foresight (PF) target setting, covering four scenarios of net zero ambitions (NZ1-NZ4). A summary of model results can be found in Document S1. Tables S7–S24.

### Quantification and statistical analysis

Quantification of scenarios in this study were executed in GAMS using CPLEX solver. Spatial analyses of model input data and model results were conducted using QGIS.
