# Supplementary material for: Myopic versus perfect foresight target setting for Indonesia’s net zero electricity transition
Source: iScience. 2025 Jun 2;28(7):112813. doi: 10.1016/j.isci.2025.112813 (PMC12271591; doi:10.1016/j.isci.2025.112813)
Supplement: Document S1. Figures S1–S3, and Tables S1–S24, and Methods S1 [file mmc1.pdf]

iScience, Volume *28*

Supplemental information

Myopic versus perfect foresight target setting for Indonesia's net zero electricity transition

Bintang Yuwono, Lukas Kranzl, Reinhard Haas, and Ping Yowargana

Document S1. Figures S1—S3, Tables S1—S24, and Methods S1

Contents:

Figure S1. Schematic overview of SELARU modelling framework, related to **Methods S1**.
Figure S2. Spatial representation for SELARU Indonesia. SELARU Indonesia comprises 516 nodes of supply-demand balancing regions (green dots with associated bounding area outlined in black) and 1,624 possible inter-nodal connections (lines in green), related to **Methods S1**.
Figure S3. Model input of changes in overnight investment costs of electricity generation technologies.
Table S1. Model input of fuel prices and specifications.
Table S2. Model input of electricity generation technologies.
Table S3. Model input of transformer substation technologies.
Table S4. Model input of transmission line technologies.
Table S5. Model input of CO_2 transport technologies.
Table S6. Model input of demand scenarios.
Table S7. Model input of topography and CAPEX spatial correction.
Table S8. Model input of geothermal and hydropower resource potential.
Table S9. Model input of land availability and solar resource potential.
Table S10. Model input of wind resource potential.
Table S11. Model input of CO_2 storage potential.
Table S12. Model input of stock electricity generation capacities.
Table S13. Model input of stock transmission substation capacities.
Table S14. Model input of stock transmission line capacities.
Table S15. Model input of planned and under construction electricity generation capacities.
Table S16. Model input of planned and under construction electricity transmission line capacities.
Table S17. Model results of annual system costs.
Table S18. Model results of capital investment.
Table S19. Model results of annual CO2 emissions.
Table S20. Model results of installed electricity generation capacity.
Table S21. Model results of annual electricity generation.
Table S22. Model results of installed capacity of transmission substation.
Table S23. Model results of installed capacity of transmission lines.
Table S24. Model results of installed capacity of CO_2 transport.
Methods S1. SELARU – MILP Formulation.

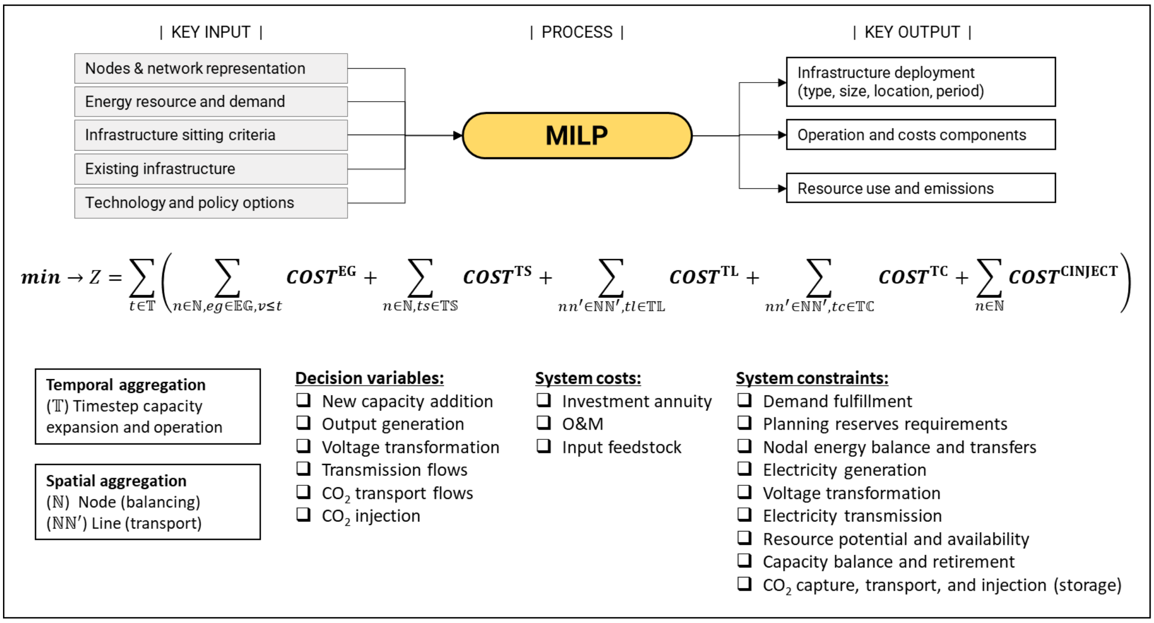

Figure S1. Schematic overview of SELARU modelling framework, related to **Methods S1**.

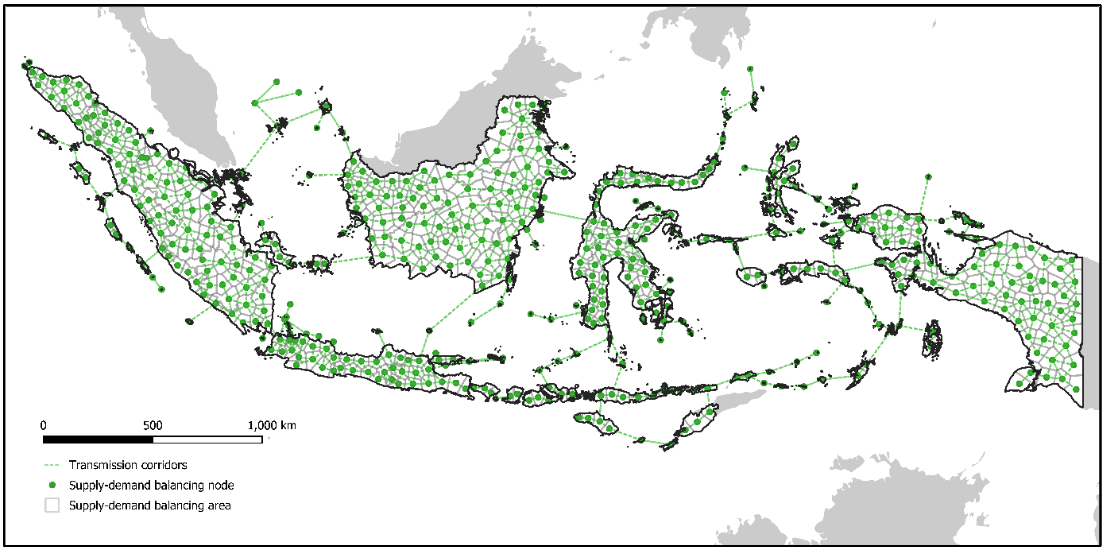

Figure S2. Spatial representation for SELARU Indonesia. SELARU Indonesia comprises 516 nodes of supply-demand balancing regions (green dots with associated bounding area outlined in black) and 1,624 possible inter-nodal connections (lines in green), related to **Methods S1**.

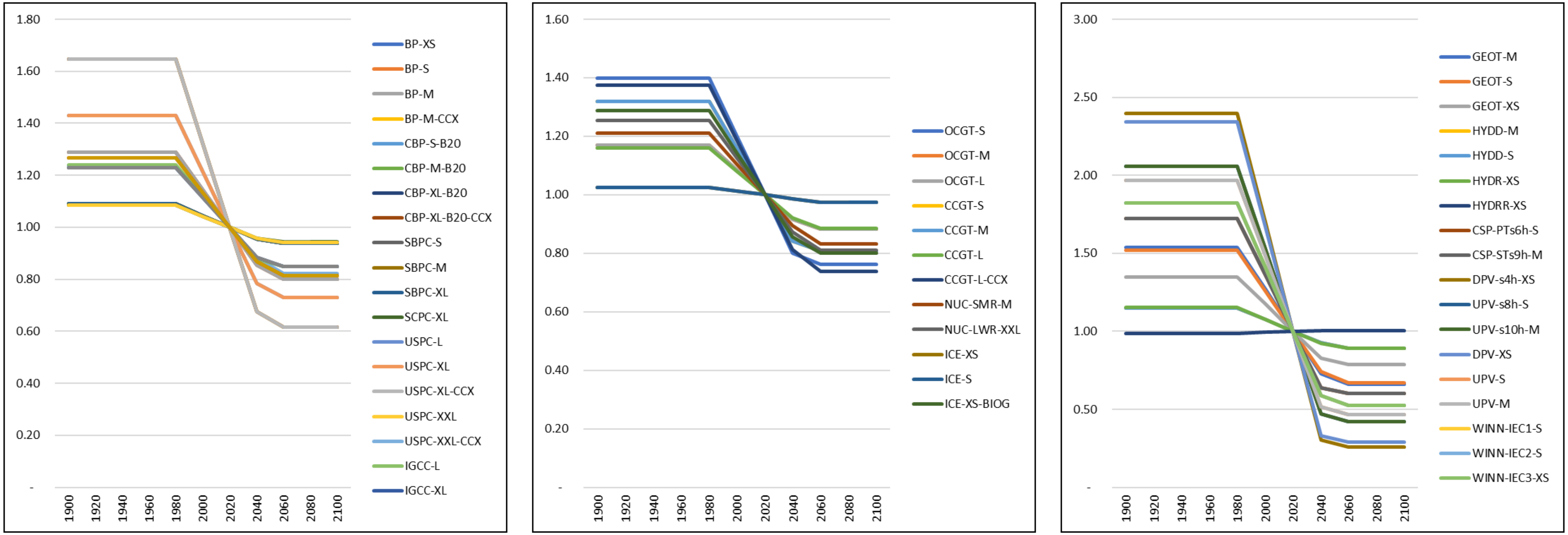

Figure S3. Model input of changes of overnight investment costs of electricity generation technologies (relative to base year 2020 = 1).

References:
ATB 2022 US$2020 https://data.nrel.gov/submissions/115
ESDM 2021 US$2020 https://gatrik.esdm.go.id/assets/uploads/download_index/files/c4d42-technology-data-for-the-indonesian-power-sector-2024-annoteret-af-kb-.pdf

Table S1. Model input of fuel prices and specifications.

	LHV	Price		GHGs		
	(GJ/ton)	($/ton)	($/GJ)	(kgCO2/GJ)	(kgCH4/GJ)	(kgNOx/GJ)
Biomass solid	16.74	78.03	4.66	100.0	30.0	4.0
Biogas	16.67	142.17	8.53	54.6	1.0	0.1
Biodiesel	38.26	1,217.07	31.81	70.8	3.0	0.6
Bioethanol	26.70	1,078.93	40.41	70.8	3.0	0.6
Diesel	42.60	1,525.51	35.81	74.1	3.0	0.6
Coal	25.10	65.03	2.59	96.1	10.0	1.5
Natural gas	47.10	327.29	6.95	56.1	1.0	0.1
Nuclear	3,900,000	1.66E+06	0.43			

References:

Heating values https://world-nuclear.org/information-library/facts-and-figures/heat-values-of-various-fuels.aspx
https://www.engineeringtoolbox.com/fuels-higher-calorific-values-d_169.html

Emissions factors https://www.ipcc-nggip.iges.or.jp/public/2006gl/pdf/2_Volume2/V2_2_Ch2_Stationary_Combustion.pdf

Fuel prices https://www2.deloitte.com/content/dam/Deloitte/ca/Documents/energy-resources/ca-en-energy-resources-industrials-o-g-price-forecast-report-Q4-aoda.pdf?icid=commentaryEN
https://www.eia.gov/outlooks/steo/report/prices.php
https://migas.esdm.go.id/uploads/harga-indek-pasar-/2020-hip/hip-solar-dalam-rangka-perhitungan-selisih-tahun-2020.pdf
https://www.minerba.esdm.go.id/harga_acuan
https://jdih.esdm.go.id/index.php/web/result/2355/detail
https://www.liputan6.com/bisnis/read/4401992/menengok-harga-keekonomian-biomassa-bahan-baku-cofiring-pltu
https://winrock.org/wp-content/uploads/2016/05/CIRCLE-Handbook-2nd-Edition-EN-25-Aug-2015-MASTER-rev02-final-new02-edited.pdf
https://www.iea.org/reports/outlook-for-biogas-and-biomethane-prospects-for-organic-growth/sustainable-supply-potential-and-costs
https://world-nuclear.org/information-library/economic-aspects/economics-of-nuclear-power.aspx#:~:text=Its%202020%20report%2C%20Capital%20Cost,%2FkWe%20(overnight%20cost).
https://www.eia.gov/opendata/v1/qb.php?category=40290&sdid=SEDS.NUETD.WI.A
https://www.eia.gov/uranium/marketing/#:~:text=During%202021%2C%2019%25%20of%20the,per%20pound%20(Table%207).
https://www.world-nuclear.org/uploadedfiles/org/info/pdf/economicsnp.pdf

Table S2. Model input of electricity generation technologies.

	Typical capacity			Technical lifetime	Economic lifetime	Construction lead years	Capital recovery	Annualized CAPEX	CAPEX+ IDC@6%	CAPEX	OMF	FOM	VOM
	(MW)	[i]	[c]	(y)	(y)	(y)	factor	(US$/kW)			(% CAPEX)	(US$/kW)	(US$/MWh)
BP-XS	1		1	25	20	2	0.0872	237.97	2,729.44	2,649.95	2.4%	63.1	3.06
BP-S	10	1		25	20	2	0.0872	201.36	2,309.53	2,242.26	2.4%	53.4	3.06
BP-M	150	1		25	20	2	0.0872	183.05	2,099.57	2,038.42	2.4%	48.5	3.06
BP-M-CCX	150	1		25	20	2	0.0872	361.52	4,146.65	4,025.88	2.3%	91.1	6.22
CBP-S	10	1		25	20	2	0.0872	262.97	3,016.28	2,928.43	2.7%	80.4	0.60
CBP-M	150	1		25	20	2	0.0872	234.53	2,690.08	2,611.73	2.7%	71.7	0.60
CBP-L	600	1		25	20	2	0.0872	195.41	2,241.36	2,176.07	2.7%	59.7	0.60
CBP-L-CCX	600	1		25	20	2	0.0872	311.24	3,569.93	3,465.96	2.4%	83.9	3.76
SBPC-S	10	1		40	25	4	0.0782	158.26	2,023.11	1,849.87	2.7%	50.8	0.13
SBPC-M	150	1		40	25	4	0.0782	143.87	1,839.19	1,681.70	2.7%	46.2	0.13
SBPC-L	600	1		40	25	4	0.0782	129.49	1,655.27	1,513.53	2.7%	41.6	0.13
SCPC-L	600	1		40	25	4	0.0782	122.07	1,560.53	1,426.89	2.9%	42.0	0.12
USPC-M	300	1		40	25	4	0.0782	159.05	2,033.14	1,859.04	3.7%	69.2	0.11
USPC-L	600	1		40	25	4	0.0782	145.79	1,863.72	1,704.12	3.7%	63.5	0.11
USPC-L-CCX	600	1		40	25	4	0.0782	315.83	4,037.31	3,691.58	2.8%	104.7	3.27
USPC-XL	1100	1		40	25	4	0.0782	132.54	1,694.29	1,549.20	3.7%	57.7	0.11
USPC-XL-CCX	1100	1		40	25	4	0.0782	302.57	3,867.88	3,536.66	2.8%	100.3	3.27
IGCC-M	250	1		40	25	4	0.0782	230.20	2,942.71	2,690.71	2.5%	67.3	0.12
IGCC-L	600	1		40	25	4	0.0782	209.27	2,675.19	2,446.10	2.5%	61.2	0.12
IGCC-L-CCX	600	1		40	25	4	0.0782	292.11	3,734.12	3,414.35	2.1%	70.2	5.52
IGCC-XL	1200	1		40	25	4	0.0782	188.34	2,407.67	2,201.49	2.5%	55.0	0.12
IGCC-XL-CCX	1200	1		40	25	4	0.0782	271.18	3,466.60	3,169.74	2.1%	65.2	5.52
OCGT-S	10	1		30	25	3	0.0782	71.66	916.10	863.27	4.7%	40.4	4.79
OCGT-M	100	1		30	25	3	0.0782	65.15	832.82	784.79	4.7%	36.7	4.79
OCGT-L	300	1		30	25	3	0.0782	58.63	749.54	706.31	4.7%	33.0	4.79
CCGT-S	60	1		30	25	3	0.0782	70.06	895.55	843.91	3.4%	28.7	2.34
CCGT-M	180	1		30	25	3	0.0782	64.22	820.92	773.58	3.4%	26.3	2.34
CCGT-L	430	1		30	25	3	0.0782	58.38	746.29	703.25	3.4%	24.0	2.34
CCGT-L-CCX	430	1		30	25	3	0.0782	155.68	1,990.12	1,875.35	1.8%	33.1	3.57
NUC-SMR-M	200	1		60	30	6	0.0726	532.93	7,335.63	6,309.93	1.5%	96.8	3.06
NUC-LWR-L	1000	1		60	30	6	0.0726	520.01	7,157.89	6,157.05	2.0%	121.0	2.42
ICE-XS	0.5		1	20	15	2	0.1030	112.41	1,091.78	1,059.98	1.0%	10.6	6.52
ICE-S	20	1		20	15	2	0.1030	86.47	839.83	815.37	1.0%	8.2	6.52
ICE-XS-BIOG	1.2		1	30	20	2	0.0872	255.81	2,934.15	2,848.69	4.5%	128.5	0.11
GEOT-M	55	1		50	20	3	0.1000	377.19	4,326.34	4,076.84	1.3%	51.0	0.25
GEOT-S	10	1		50	20	3	0.0872	471.49	5,407.93	5,096.05	1.3%	66.2	0.38
GEOT-XS	0.5		1	50	20	3	0.0872	565.79	6,489.51	6,115.26	1.3%	79.5	0.38
HYDD-L	150	1		100	30	10	0.0726	203.00	2,794.27	2,119.96	1.8%	38.4	0.66
HYDD-M	50	1		100	30	10	0.0726	223.50	3,076.38	2,333.99	1.8%	42.7	0.66
HYDD-S	5		1	100	30	10	0.0726	268.20	3,691.66	2,800.79	2.0%	55.0	0.51
HYDR-S	5		1	100	30	2	0.0726	205.92	2,834.42	2,751.87	2.0%	54.0	0.51
HYDR-XS	0.1		1	100	30	1	0.0726	239.90	3,302.24	3,302.24	2.0%	64.8	0.51
CSP-PTs6h-S	5		1	30	25	3	0.0782	522.99	6,685.56	6,300.00	1.2%	74.5	
CSP-STs9h-M	20	1		30	25	3	0.0782	639.21	8,171.24	7,700.00	1.2%	91.1	
DPV-s4h-XS	0.007		1	30	25	1	0.0782	369.45	4,722.78	4,722.78	1.8%	86.6	
UPV-s8h-S	10		1	30	25	1	0.0782	295.65	3,779.40	3,779.40	1.8%	69.3	
UPV-s10h-M	100	1		30	25	2	0.0782	249.19	3,185.51	3,092.73	1.8%	56.7	
DPV-XS	0.007		1	30	25	1	0.0782	123.13	1,574.07	1,574.07	1.1%	17.2	
UPV-S	10		1	30	25	1	0.0782	119.55	1,528.20	1,528.20	1.2%	18.5	
UPV-M	100	1		30	25	2	0.0782	84.34	1,078.13	1,046.73	1.8%	19.1	
WINN-IEC1-S	700		1	30	25	1	0.0782	119.59	1,528.81	1,528.81	4.0%	61.2	
WINN-IEC2-S	250		1	30	25	1	0.0782	183.38	2,344.18	2,344.18	4.0%	93.8	
WINN-IEC3-XS	0.85		1	30	25	1	0.0782	318.92	4,076.84	4,076.84	1.8%	74.6	

Table S2. Model input of electricity generation technologies. (continued)

		Typical capacity (MW)	Max CF	Min CF	Max full load hours (h)	Reserve capacity credit	Heat rate (GJ/MWh)	Energy Efficiency	Land-use (ha/MW)
Biomass-pellets firing SB 1MW	BP-XS	1	76.0%	22.8%	6,658	93%	11.90	0.77	1.42
Biomass-pellets firing SB 10MW	BP-S	10	80.0%	24.0%	7,008	93%	11.52	0.78	1.42
Biomass-pellets firing SB 150MW	BP-M	150	85.0%	25.5%	7,446	93%	11.16	0.81	1.42
Biomass-pellets firing SB 150MW +CO2 capture	BP-M-CCX	150	90.0%	54.0%	7,884	93%	14.88	0.65	1.42
Direct cofiring biomass-coal SB 10MW	CBP-S	10	75.0%	22.5%	6,570	90%	9.65	0.37	1.42
Direct cofiring biomass-coal SB 150MW	CBP-M	150	80.0%	24.0%	7,008	90%	9.65	0.37	1.42
Direct cofiring biomass-coal SB 600MW	CBP-L	600	85.0%	25.5%	7,446	90%	8.93	0.40	1.42
Direct cofiring biomass-coal SB 600MW +CO2 capture	CBP-L-CCX	600	90.0%	54.0%	7,884	90%	11.16	0.32	1.42
Sub-critic pulverized coal firing 10MW	SBPC-S	10	75.0%	22.5%	6,570	92%	10.66	0.34	0.45
Sub-critic pulverized coal firing 150MW	SBPC-M	150	75.0%	22.5%	6,570	92%	10.50	0.34	0.40
Sub-critic pulverized coal firing 600MW	SBPC-L	600	75.0%	22.5%	6,570	92%	10.20	0.35	0.40
Super-critic pulverized coal firing 600MW	SCPC-L	600	80.0%	24.0%	7,008	92%	9.65	0.37	0.36
Ultra super-critic pulverized coal firing 300MW	USPC-M	300	80.0%	24.0%	7,008	92%	8.93	0.40	0.36
Ultra super-critic pulverized coal firing 600MW	USPC-L	600	85.0%	25.5%	7,446	92%	8.71	0.41	0.32
Ultra super-critic pulverized coal firing 600MW +CO2 capture	USPC-L-CCX	600	90.0%	54.0%	7,884	92%	10.66	0.34	0.32
Ultra super-critic pulverized coal firing 1100MW	USPC-XL	1100	85.0%	25.5%	7,446	92%	8.50	0.42	0.28
Ultra super-critic pulverized coal firing 1100MW +CO2 capture	USPC-XL-CCX	1100	90.0%	54.0%	7,884	92%	10.50	0.34	0.28
Integrated gasification coal combined-cycle 250MW	IGCC-M	250	80.0%	24.0%	7,008	92%	8.93	0.40	0.28
Integrated gasification coal combined-cycle 600MW	IGCC-L	600	85.0%	25.5%	7,446	92%	8.50	0.42	0.28
Integrated gasification coal combined-cycle 600MW +CO2 capture	IGCC-L-CCX	600	90.0%	54.0%	7,884	92%	10.50	0.34	0.28
Integrated gasification coal combined-cycle 1200MW	IGCC-XL	1200	85.0%	25.5%	7,446	92%	8.31	0.43	0.24
Integrated gasification coal combined-cycle 1200MW +CO2 capture	IGCC-XL-CCX	1200	90.0%	54.0%	7,884	92%	10.20	0.35	0.24
Open-cycle gas turbine 2x5MW	OCGT-S	10	70.0%	7.0%	6,132	92%	10.66	0.34	0.07
Open-cycle gas turbine 2x50MW	OCGT-M	100	75.0%	22.5%	6,570	92%	10.50	0.34	0.08
Open-cycle gas turbine 6x50MW	OCGT-L	300	80.0%	24.0%	7,008	92%	10.20	0.35	0.09
Combined cycle gas turbine 60MW	CCGT-S	60	75.0%	22.5%	6,570	92%	6.74	0.53	0.08
Combined cycle gas turbine 3x60MW	CCGT-M	180	78.0%	23.4%	6,833	92%	6.49	0.55	0.09
Combined cycle gas turbine 430MW	CCGT-L	430	81.0%	24.3%	7,096	92%	6.38	0.56	0.09
Combined cycle gas turbine 430MW +CO2 capture	CCGT-L-CCX	430	85.0%	51.0%	7,446	92%	7.14	0.50	0.14
Nuclear Small Modular Reactor 200MW	NUC-SMR-M	200	83.0%	24.9%	7,271	95%	11.90	0.30	0.36
Nuclear Light Water Reactor 1GW	NUC-LWR-L	1000	85.0%	25.5%	7,446	95%	11.90	0.30	0.36
Internal combustion engine <1MW	ICE-XS	0.5	80.0%	1.6%	7,008	90%	9.40	0.38	0.08
Internal combustion engine 4x5MW	ICE-S	20	80.0%	4.0%	7,008	90%	7.94	0.45	0.08
Biogas-ICE 1.2MW +Covered Lagoon Anaerobic Digester	ICE-XS-BIOG	1.2	70.0%	21.0%	6,132	80%	10.50	0.34	7.00
Geothermal 55MW (flash/dry, high temp)	GEOT-M	55	85.0%	25.5%	7,446	95%		0.15	3.00
Geothermal 10MW (binary/condensing, low temp)	GEOT-S	10	85.0%	8.5%	7,446	95%		0.10	3.00
Geothermal 5MW (binary/condensing, low temp)	GEOT-XS	0.5	85.0%	8.5%	7,446	95%		0.10	3.00
Large hydropower 150MW, dam	HYDD-L	150	88.0%	26.4%	7,709	98%		0.95	3.24
Medium hydropower 50MW, dam	HYDD-M	50	88.0%	26.4%	7,709	98%		0.95	3.24
Small hydropower 5MW, dam	HYDD-S	5	88.0%	8.8%	7,709	98%		0.95	2.54
Small hydropower 5x1MW, run-off-river	HYDR-S	5	88.0%	4.4%	7,709	98%		0.80	2.54
Very-small hydropower 100kW, run-off-river	HYDR-XS	0.1	88.0%	1.8%	7,709	98%		0.80	1.39
Concentrating Solar Power, Parabolic Through 5MW +6h storage	CSP-PTs6h-S	5	43.0%	0.9%	3,767	60%		0.16	2.02
Concentrating Solar Power, Solar Tower 20MW +9h storage	CSP-STs9h-M	20	65.0%	3.3%	5,694	70%		0.20	3.24
Small PV 7kW +4h storage	DPV-s4h-XS	0.007	27.9%	0.6%	2,444	47%		0.17	1.29
Medium PV 10MW +8h storage	UPV-s8h-S	10	36.0%	0.7%	3,154	78%		0.17	2.23
Large PV 100MW +10h storage	UPV-s10h-M	100	41.0%	2.1%	3,592	98%		0.17	2.47
Small PV 7kW	DPV-XS	0.007	16.0%		1,402			0.19	1.29
Medium PV 10MW	UPV-S	10	16.0%		1,402			0.20	2.23
Large PV 100MW	UPV-M	100	16.0%		1,402			0.20	2.47
Onshore wind power IEC1 20x3.5MW	WINN-IEC1-S	700	50.0%	2.5%	4,380	10%		0.40	18.09
Onshore wind power IEC2 10x2.5MW	WINN-IEC2-S	250	35.0%	0.7%	3,066	9%		0.30	12.14
Onshore wind power IEC3 15x850kW	WINN-IEC3-XS	0.85	35.0%	0.7%	3,066	7%	11.90	0.20	12.14

Table S2. Model input of electricity generation technologies. (continued)

	**Typical capacity**	**Surface area**	**Solar multiple**	**Array density**	**Module efficiency**	**Cell efficiency**	**Inverter loading ratio**	**Rated wind speed**	**Specific power density**	**Swept area**	**Rotor blade length**	**Hub height**
	**(MW)**	**(m2/MW)**	**(kWp/m2)**	**(kWe/kWp)**	**(MWp/MW)**	**(m/s)**	**(W/m2)**	**(m2)**	**(m)**	**(m)**		
CSP-PTs6h-S	5	8430.96	2.4									
CSP-STs9h-M	20	11990.70	2.7									
DPV-s4h-XS	0.007	4884.00		175	19.0%	22.0%	1.17					
UPV-s8h-S	10	4535.15		175	19.5%	22.0%	1.26					
UPV-s10h-M	100	4395.60		175	20.0%	22.0%	1.3					
DPV-XS	0.007	4884.00		175	19.0%	22.0%	1.17					
UPV-S	10	4535.15		175	19.5%	22.0%	1.26					
UPV-M	100	4395.60		175	20.0%	22.0%	1.3					
WINN-IEC1-S	700							10	320	9856	56	100
WINN-IEC2-S	250							8.5	300	12474	63	100
WINN-IEC3-XS	0.85							7.5	205	14532.57143	68	100

References:

IRENA RENEWABLES COSTS 2020	https://www.irena.org/publications/2021/Jun/Renewable-Power-Costs-in-2020
AEO 2020 CAPITAL COSTS	https://www.eia.gov/analysis/studies/powerplants/capitalcost/pdf/capital_cost_AEO2020.pdf
NREL ATB	https://atb.nrel.gov/
Costs Calculation	https://atb.nrel.gov/electricity/2021/equations_&_variables#equations
PV + Battery Storage Peaking Capacity	https://www.nrel.gov/docs/fy19osti/74184.pdf
PV + Battery Storage Costs	https://www.nrel.gov/docs/fy21osti/77324.pdf
Wind Turbine	https://www.nrel.gov/docs/fy13osti/56290.pdf
Wind Turbine	https://globalwindatlas.info/about/ReleaseNotes
PV+Storage	https://www.nrel.gov/docs/fy21osti/78882.pdf
Landuse	https://www.nrel.gov/analysis/tech-size.html
	https://www.gem.wiki/Concentrating_solar_power_land_use
	https://ourworldindata.org/land-use-per-energy-source
	https://docs.wind-watch.org/US-footprints-Strata-2017.pdf
DJK ESDM - DANIDA	https://ens.dk/sites/ens.dk/files/Globalcooperation/technology_data_for_the_indonesian_power_sector_-_final.pdf
CO2 Capture	https://ieaghg.org/docs/general_publications/cocapture.pdf
CF Ranges	https://www.eia.gov/todayinenergy/detail.php?id=22832

Table S3. Model input of transformer substation technologies.

		Typical capacity	Economic lifetime	Construction lead years	Capital recovery	Annualized CAPEX	CAPEX+ IDC@4%	CAPEX
		(MW)	(y)	(y)	factor	(US$/MW)		
GI_EHV 1000MW	EHV	500	25	3	0.06	1574.24	24,592.95	23,634.94
GI_HHV 500MW	HHV	50	25	3	0.06	554.73	8,666.11	8,328.52
GI_HV 50MW	HV	25	25	2	0.06	253.77	3,964.38	3,886.64
GI_MV 1MW	MV	1	25	1	0.06	107.03	1,672.04	1,672.04

Table S4. Model input of transmission line technologies

		Typical capacity	Losses rate	Economic lifetime	Construction lead years	Capital Recovery	Annualized CAPEX	CAPEX+ IDC@4%	CAPEX
		(MW)	(/10km)	(y)	(y)	factor	(US$/MW-km)		
500/765kV AC 1000-2000MW	EHV	500	0.07%	40	3	0.05	69.89	1383.27	1,329.38
150/275 kV AC 100-500MW	HHV	50	1.20%	40	2	0.05	76.90	1522.01	1,492.17
70kV AC 50MW	HV	25	3.50%	40	2	0.05	79.62	1575.82	1,544.92
30kV AC 1MW	MV	1	11.00%	40	1	0.05	153.32	3034.57	3,034.57

References:
https://iea-etsap.org/E-TechDS/PDF/E12_el-t&d_KV_Apr2014_GSOK.pdf
https://www.adb.org/sites/default/files/linked-documents/47296-001-ea.pdf
https://www.wecc.org/Administrative/TEPPC_TransCapCostCalculator_E3_2019_Update.xlsx

*) National cost of transmission and distribution is approximated using constants of 10% and 30% of total system costs.

Table S5. Model input of CO_2 transport technologies.

		Typical capacity	Economic lifetime	Construction lead years	Capital Recovery	Annualized CAPEX	CAPEX+ IDC@4%	CAPEX	OMF	FOM
		(MW)	(y)	(y)	factor	(US$/MtCO$_2$pa-km)			(% CAPEX)	(US$/MtCO$_2$pa-km)
CO2 pipeline, on-shore 2.5 MtCO2pa	pipeS	2.5	40	2	0.05	551,217	10,910,119	10,910,119	0.52%	56,922
CO2 pipeline, on-shore 10 MtCO2 pa	pipeM	10	40	3	0.05	144,035	2,850,862	2,850,862	0.50%	14,231
CO2 pipeline, on-shore 20 MtCO2 pa	pipeL	20	40	3	0.05	91,790	1,816,772	1,816,772	0.39%	7,115

Table S6. Model input of electricity demand scenario.

**In TWh y-1**	**2020**					**2040**					**2060**					**2080**					**2100**				
	sum.	avg.	max	min	stdev	sum.	avg.	max	min	stdev	sum.	avg.	max	min	stdev	sum.	avg.	max	min	stdev	sum.	avg.	max	min	stdev
Aceh	2.94	0.16	0.55	0.00	0.16	5.26	0.29	1.05	0.01	0.28	7.86	0.44	1.58	0.01	0.41	11.41	0.63	2.31	0.01	0.59	16.32	0.91	3.32	0.02	0.85
Bali	4.95	1.24	3.58	0.05	1.62	9.00	2.25	6.61	0.09	3.00	13.65	3.41	10.02	0.14	4.56	20.13	5.03	14.78	0.20	6.72	29.25	7.31	21.48	0.29	9.77
Banten	22.27	5.57	11.66	0.44	5.66	37.89	9.47	20.47	0.70	9.80	53.37	13.34	28.85	0.99	13.81	72.51	18.13	39.20	1.34	18.76	96.23	24.06	52.03	1.77	24.90
Bengkulu	1.01	0.14	0.61	0.00	0.21	1.85	0.26	1.11	0.00	0.38	2.83	0.40	1.70	0.00	0.59	4.24	0.61	2.55	0.01	0.88	6.28	0.90	3.77	0.01	1.30
DI Yogyakarta	3.01	3.01	3.01	3.01		5.28	5.28	5.28	5.28		7.71	7.71	7.71	7.71		10.92	10.92	10.92	10.92		15.19	15.19	15.19	15.19	
DKI Jakarta	31.08	10.36	31.01	0.03	17.88	51.54	17.18	51.41	0.05	29.64	70.60	23.53	70.43	0.07	40.61	93.10	31.03	92.88	0.09	53.56	119.69	39.90	119.41	0.12	68.86
Gorontalo	0.60	0.15	0.35	0.02	0.14	1.11	0.28	0.64	0.05	0.26	1.72	0.43	1.00	0.07	0.40	2.62	0.66	1.52	0.11	0.61	3.96	0.99	2.30	0.16	0.92
Jambi	2.01	0.25	0.55	0.12	0.15	3.61	0.45	0.98	0.21	0.26	5.41	0.68	1.47	0.31	0.40	7.89	0.99	2.15	0.45	0.58	11.36	1.42	3.10	0.65	0.83
Jawa Barat	49.63	4.96	13.53	0.62	4.66	87.24	8.72	23.94	1.08	8.35	127.08	12.71	34.78	1.57	12.19	178.80	17.88	48.88	2.22	17.17	246.09	24.61	67.23	3.05	23.64
Jawa Tengah	25.09	2.09	5.93	0.00	1.76	44.20	3.68	10.61	0.01	3.13	64.59	5.38	15.58	0.01	4.60	91.26	7.60	22.07	0.02	6.51	126.25	10.52	30.57	0.02	9.02
Jawa Timur	37.61	2.21	9.24	0.01	2.23	65.78	3.87	16.31	0.01	3.92	95.34	5.61	23.70	0.02	5.69	133.45	7.85	33.20	0.03	7.98	182.72	10.75	45.49	0.04	10.93
Kalimantan Barat	2.72	0.09	0.76	0.00	0.14	4.91	0.17	1.39	0.00	0.25	7.37	0.25	2.09	0.00	0.38	10.78	0.37	3.06	0.01	0.56	15.53	0.54	4.41	0.01	0.81
Kalimantan Selatan	2.93	0.37	1.12	0.01	0.35	5.32	0.66	2.07	0.01	0.64	8.05	1.01	3.15	0.02	0.98	11.87	1.48	4.66	0.03	1.45	17.24	2.16	6.79	0.04	2.11
Kalimantan Tengah	1.49	0.07	0.22	0.00	0.07	2.73	0.13	0.40	0.00	0.13	4.19	0.20	0.62	0.00	0.20	6.26	0.30	0.92	0.01	0.29	9.27	0.44	1.37	0.01	0.43
Kalimantan Timur	2.04	0.10	0.55	0.00	0.14	3.60	0.17	0.91	0.00	0.24	5.28	0.25	1.32	0.00	0.35	7.54	0.36	1.87	0.00	0.50	10.58	0.50	2.62	0.00	0.71
Kalimantan Utara	0.34	0.03	0.14	0.00	0.04	0.63	0.06	0.26	0.00	0.08	1.00	0.09	0.42	0.00	0.13	1.55	0.14	0.65	0.00	0.20	2.40	0.22	1.01	0.01	0.30
Kepulauan Bangka Belitung	1.22	0.20	0.45	0.10	0.14	2.26	0.38	0.84	0.18	0.26	3.52	0.59	1.32	0.28	0.40	5.36	0.89	2.01	0.42	0.61	8.09	1.35	3.04	0.64	0.93
Kepulauan Riau	3.33	0.30	2.14	0.00	0.63	5.88	0.53	3.74	0.00	1.10	8.65	0.79	5.52	0.00	1.62	12.33	1.12	7.89	0.00	2.32	17.29	1.57	11.07	0.00	3.25
Lampung	4.96	0.71	1.61	0.12	0.49	8.99	1.28	2.92	0.22	0.88	13.59	1.94	4.42	0.34	1.34	19.97	2.85	6.49	0.50	1.96	28.90	4.13	9.40	0.72	2.84
Maluku	0.55	0.02	0.22	0.00	0.04	0.97	0.03	0.39	0.00	0.07	1.44	0.05	0.57	0.00	0.11	2.07	0.07	0.83	0.00	0.15	2.96	0.11	1.18	0.00	0.22
Maluku Utara	0.59	0.03	0.16	0.00	0.04	1.07	0.06	0.32	0.00	0.07	1.64	0.09	0.48	0.01	0.11	2.44	0.14	0.72	0.01	0.16	3.60	0.20	1.07	0.01	0.24
Nusa Tenggara Barat	2.15	0.27	0.74	0.03	0.24	4.00	0.50	1.45	0.05	0.47	6.24	0.78	2.28	0.08	0.74	9.51	1.19	3.48	0.12	1.13	14.35	1.79	5.26	0.18	1.70
Nusa Tenggara Timur	1.13	0.06	0.31	0.00	0.07	2.07	0.10	0.59	0.00	0.13	3.17	0.16	0.91	0.00	0.20	4.73	0.24	1.35	0.00	0.29	6.99	0.35	2.01	0.00	0.43
Papua	1.13	0.02	0.34	0.00	0.06	2.06	0.04	0.54	0.00	0.10	3.13	0.06	0.84	0.00	0.15	4.64	0.09	1.27	0.00	0.23	6.82	0.13	1.87	0.00	0.33
Papua Barat	0.55	0.02	0.15	0.00	0.04	1.00	0.04	0.28	0.00	0.06	1.52	0.05	0.43	0.00	0.10	2.26	0.08	0.64	0.00	0.15	3.36	0.12	0.95	0.00	0.22
Riau	4.97	0.33	1.07	0.00	0.28	9.15	0.61	2.06	0.00	0.52	14.07	0.94	3.24	0.00	0.81	21.06	1.40	4.90	0.00	1.22	31.10	2.07	7.28	0.00	1.80
Sulawesi Barat	0.42	0.08	0.16	0.00	0.06	0.79	0.16	0.30	0.00	0.11	1.24	0.25	0.47	0.00	0.17	1.92	0.38	0.73	0.00	0.27	2.96	0.59	1.13	0.01	0.42
Sulawesi Selatan	5.98	0.26	2.50	0.00	0.51	10.82	0.47	4.53	0.00	0.93	16.30	0.71	6.85	0.01	1.40	23.87	1.04	10.03	0.01	2.05	34.40	1.50	14.46	0.01	2.95
Sulawesi Tengah	1.27	0.06	0.28	0.00	0.07	2.35	0.10	0.44	0.00	0.12	3.63	0.16	0.70	0.00	0.19	5.49	0.24	1.08	0.00	0.29	8.23	0.36	1.63	0.00	0.43
Sulawesi Tenggara	1.06	0.07	0.27	0.00	0.08	1.97	0.13	0.51	0.00	0.15	3.06	0.20	0.79	0.00	0.23	4.66	0.31	1.20	0.00	0.35	7.04	0.47	1.82	0.00	0.52
Sulawesi Utara	1.86	0.19	0.98	0.00	0.30	3.35	0.34	1.79	0.00	0.54	5.04	0.50	2.69	0.00	0.82	7.39	0.74	3.94	0.00	1.20	10.67	1.07	5.70	0.00	1.73
Sumatera Barat	3.43	0.29	1.01	0.01	0.34	5.96	0.50	1.73	0.03	0.58	8.59	0.72	2.50	0.04	0.84	12.01	1.00	3.49	0.05	1.18	16.48	1.37	4.80	0.07	1.61
Sumatera Selatan	5.39	0.38	2.17	0.00	0.53	9.73	0.69	3.98	0.00	0.98	14.63	1.04	6.02	0.01	1.48	21.37	1.53	8.81	0.01	2.17	30.72	2.19	12.68	0.01	3.12
Sumatera Utara	11.19	0.59	5.39	0.01	1.20	19.47	1.02	9.48	0.01	2.11	28.10	1.48	13.70	0.02	3.05	39.20	2.06	19.13	0.03	4.26	53.58	2.82	26.15	0.04	5.82

References:

STATISTIK PLN 2021	https://web.pln.co.id/statics/uploads/2022/08/Statistik-PLN-2021-29-7-22-Final.pdf
BPS - Sensus Penduduk 2020	https://www.bps.go.id/pressrelease/2021/01/21/1854/hasil-sensus-penduduk-2020.html
BPS - PDRB Regional 2014-2018	https://www.bps.go.id/publication/2019/10/04/9812a1c4ea25298004839596/produk-domestik-regional-bruto-kabupaten-kota-di-indonesia-2014-2018.html
SSP2 Scenario Database	https://www.sciencedirect.com/science/article/pii/S0959378016300681

*) Peak demand was approximated using load factor to annual demand (78.32%). The planning reserve margin is set to be 35%. Demand at node is supplied with electricity accounting for distribution line losses (7.22%) and distribution substation own-use (0.27%). In non-spatial application, national aggregated transmission losses (9.54%) are comprised of line losses (2.08%) and own use (0.06%).

Table S7. Model input of topography and CAPEX spatial correction.

		**Area**					**Elevation**					**Roughness**					**Slope**					**CAPEX multiplier**				
	[n]	(sqkm)	avg.	max	min	stdev	(m)	avg.	max	min	stdev	(m)	avg.	max	min	stdev	(deg.)	avg.	max	min	stdev		avg.	max	min	stdev
INDONESIA		1891077					339					66					5.4					0.50				
Aceh	18	56779	3154	6762	90	1860	629	471	1486	49	442	113	104	193	28	52	9.4	8.6	16.0	2.3	4.4	1.05	0.84	1.81	0.00	0.54
Sumatera Utara	19	72321	3806	6457	1136	1648	433	386	1077	30	359	63	61	139	9	40	5.2	5.0	11.0	0.7	3.3	0.39	0.44	1.11	0.00	0.39
Sumatera Barat	14	42225	3016	7197	3	2419	478	308	757	10	297	95	70	145	11	44	7.7	5.7	12.1	0.9	3.7	0.67	0.56	1.41	0.00	0.45
Riau	15	89808	5987	8584	0	2329	51	45	147	1	42	15	14	50	2	12	1.2	1.0	3.9	0.2	0.9	0.00	0.03	0.35	0.00	0.09
Jambi	8	48939	6117	9969	4929	1663	252	260	1181	13	408	40	40	134	4	45	3.2	3.2	11.2	0.3	3.8	0.32	0.25	1.17	0.00	0.46
Sumatera Selatan	14	86962	6212	10993	2479	2153	152	148	621	7	208	24	23	77	3	25	1.9	1.9	6.4	0.2	2.1	0.05	0.15	0.76	0.00	0.25
Bengkulu	7	20026	2861	4433	397	1375	442	381	641	56	206	76	71	103	32	23	6.3	5.8	8.4	2.5	1.9	0.67	0.54	0.82	0.00	0.29
Lampung	8	33602	4200	7300	26	2301	187	201	673	18	227	35	49	117	8	41	2.9	4.1	10.2	0.6	3.5	0.05	0.37	1.05	0.00	0.42
Kepulauan Bangka Belitung	6	16706	2784	3649	1841	653	27	27	29	24	3	16	16	17	14	1	1.2	1.2	1.4	1.1	0.1	0.00	0.00	0.00	0.00	0.00
Kepulauan Riau	11	8250	750	1736	5	663	41	41	107	16	27	36	42	102	17	27	2.9	3.5	8.7	1.2	2.3	0.00	0.19	0.76	0.00	0.29
DKI Jakarta	4	654	163	643	0	320	18	5	17	1	8	6	4	6	3	1	0.4	0.3	0.4	0.2	0.1	0.00	0.00	0.00	0.00	0.00
Jawa Barat	10	37065	3707	6018	2238	1097	436	395	962	48	305	59	56	94	14	29	5.0	4.7	7.9	1.2	2.5	0.39	0.42	0.76	0.00	0.32
Jawa Tengah	12	34438	2870	5102	47	1356	286	272	573	52	156	45	47	80	23	18	3.8	4.0	6.7	1.9	1.5	0.32	0.30	0.76	0.00	0.22
DI Yogyakarta	1	3172	3172	3172	3172		212	212	212	212		39	39	39	39		3.2	3.2	3.2	3.2		0.32	0.35	0.35	0.35	
Jawa Timur	17	48048	2826	5797	41	1752	301	216	692	19	197	47	41	96	11	27	3.9	3.5	8.0	1.0	2.2	0.32	0.25	0.76	0.00	0.31
Banten	4	9357	2339	3458	1035	1036	163	160	416	23	175	39	37	81	6	32	3.2	3.0	6.7	0.4	2.6	0.32	0.19	0.70	0.00	0.34
Bali	4	5588	1397	2531	208	1136	440	340	600	195	190	75	71	95	54	19	6.3	5.9	8.0	4.6	1.6	0.67	0.57	0.76	0.41	0.19
Nusa Tenggara Barat	8	19670	2459	3185	1706	529	355	361	607	217	151	97	95	118	65	19	8.1	8.0	9.9	5.4	1.6	0.67	0.78	1.05	0.41	0.21
Nusa Tenggara Timur	20	46456	2323	7291	461	1618	390	334	628	85	148	91	93	147	30	36	7.7	7.8	12.2	2.6	3.0	0.67	0.76	1.41	0.00	0.42
Kalimantan Barat	29	147227	5077	10201	255	1971	170	144	613	14	141	51	48	144	7	35	4.1	3.9	12.0	0.5	2.9	0.39	0.28	1.11	0.00	0.33
Kalimantan Tengah	21	154166	7341	11521	4106	2153	129	129	570	9	150	34	32	102	4	31	2.7	2.6	8.5	0.3	2.6	0.05	0.17	0.82	0.00	0.26
Kalimantan Selatan	8	36751	4594	9268	22	2678	114	94	199	1	64	37	32	60	5	17	3.0	2.6	4.9	0.4	1.4	0.32	0.15	0.41	0.00	0.19
Kalimantan Timur	21	126273	6013	9882	39	2370	241	209	790	8	216	55	49	134	11	35	4.5	4.0	11.0	0.8	3.0	0.39	0.28	1.11	0.00	0.34
Kalimantan Utara	11	69977	6362	11634	1574	3177	587	440	1009	22	391	109	89	149	15	56	8.8	7.2	12.1	1.1	4.6	0.78	0.69	1.46	0.00	0.61
Sulawesi Utara	10	14522	1452	3571	2	1338	434	291	581	9	205	109	97	163	23	41	9.1	8.1	13.9	1.7	3.5	1.01	0.79	1.40	0.00	0.39
Sulawesi Tengah	23	61173	2660	4960	22	1563	624	478	1311	51	319	126	109	189	47	39	10.4	9.1	14.4	4.0	3.1	1.05	0.91	1.46	0.29	0.38
Sulawesi Selatan	23	45464	1977	5195	1	1543	575	371	1592	0	444	92	65	171	0	50	7.8	5.5	14.4	0.0	4.2	0.71	0.49	1.46	0.00	0.49
Sulawesi Tenggara	15	36576	2438	4552	9	1641	358	276	953	35	251	96	85	201	17	51	7.8	7.0	14.8	1.4	3.9	0.67	0.65	1.52	0.00	0.44
Gorontalo	4	12032	3008	3893	2229	800	407	407	552	264	118	117	118	143	91	22	9.8	9.8	12.0	7.6	1.9	1.01	1.05	1.41	0.70	0.29
Sulawesi Barat	5	16569	3314	5637	22	2157	678	502	1044	0	392	132	101	167	0	64	11.0	8.4	13.8	0.0	5.4	1.05	0.89	1.52	0.00	0.57
Maluku	30	46212	1540	5012	11	1640	293	209	729	27	172	84	86	165	11	46	7.0	7.3	14.4	0.8	3.9	0.67	0.72	1.41	0.00	0.48
Maluku Utara	18	31508	1750	3802	29	1297	273	228	353	32	97	96	93	150	27	28	7.9	7.8	13.0	2.3	2.4	0.67	0.75	1.35	0.00	0.31
Papua Barat	28	98528	3519	8899	36	2025	363	302	1058	3	305	77	69	179	5	49	6.3	5.6	14.7	0.4	4.0	0.67	0.51	1.52	0.00	0.50
Papua	54	314033	5815	10142	5	2838	510	502	2480	8	664	78	81	252	4	74	6.2	6.4	19.0	0.3	5.7	0.71	0.63	2.34	0.00	0.71

References:
INA Geoportal https://geoportal.big.go.id/
EarthEnv https://www.earthenv.org/topography

Table S8. Model input of geothermal and hydropower resource potential.

		**Geothermal Potential**					**Hydropower dam potential**					**Hydropower run-off-river potential**					**CF, Dam**					**CF, RoR**				
	[n]	(MW)	avg.	max	min	stdev	(MW)	avg.	max	min	stdev	(MW)	avg.	max	min	stdev		avg.	max	min	stdev		avg.	max	min	stdev
INDONESIA		16282					67949					17830					0.51					0.65				
Aceh	18	854	107	282	10	80	6727	477	1234	0	377	1138	81	265	0	72	0.55	0.55	0.76	0.40	0.16	0.60	0.60	0.80	0.35	0.22
Sumatera Utara	19	1387	198	388	25	138	3160	232	884	0	265	1551	119	454	0	141	0.54	0.54	0.76	0.40	0.18	0.73	0.73	0.80	0.35	0.17
Sumatera Barat	14	1019	255	408	118	119	2570	302	966	0	325	839	104	199	0	65	0.45	0.45	0.76	0.40	0.13	0.74	0.74	0.80	0.35	0.16
Riau	15	45	15	35	5	17	932	233	337	8	151	0	0	0	0	0	0.40	0.40	0.40	0.40	0.00	0.80	0.80	0.80	0.80	0.00
Jambi	8	427	142	350	3	183	1494	373	705	34	363	546	137	543	0	271	0.40	0.40	0.40	0.40	0.00	0.80	0.80	0.80	0.80	0.00
Sumatera Selatan	14	534	178	289	110	97	846	117	366	0	141	486	69	259	0	92	0.50	0.50	0.76	0.40	0.18	0.74	0.74	0.80	0.35	0.17
Bengkulu	7	569	285	389	180	148	1325	200	559	87	183	548	91	258	19	86	0.46	0.46	0.76	0.40	0.15	0.76	0.76	0.80	0.55	0.10
Lampung	8	1323	265	588	40	199	505	110	365	0	172	318	80	140	15	69	0.49	0.49	0.76	0.40	0.18	0.46	0.46	0.68	0.35	0.15
Kepulauan Bangka Belitung	6	100	25	50	5	21																				
Kepulauan Riau	11																									
DKI Jakarta	4																									
Jawa Barat	10	2615	327	1053	75	331	4138	374	940	0	357	511	51	146	0	50	0.57	0.57	0.76	0.40	0.18	0.71	0.71	0.80	0.35	0.19
Jawa Tengah	12	1072	134	332	15	118	1104	122	461	0	186	223	25	79	0	32	0.48	0.48	0.76	0.40	0.16	0.75	0.75	0.80	0.35	0.15
DI Yogyakarta	1	10	10	10	10		18	18	18	18		1	1	1	1		0.40	0.40	0.40	0.40		0.35	0.35	0.35	0.35	
Jawa Timur	17	999	125	220	25	73	525	46	234	0	73	198	20	103	0	32	0.62	0.62	0.76	0.40	0.18	0.71	0.71	0.80	0.35	0.19
Banten	4	410	137	170	115	29	204	68	204	0	118	63	21	55	2	29	0.55	0.55	0.76	0.40	0.19	0.65	0.65	0.80	0.35	0.26
Bali	4	120	60	110	10	71	10	5	10	0	7	100	50	56	44	9	0.76	0.76	0.76	0.76	0.00	0.80	0.80	0.80	0.80	0.00
Nusa Tenggara Barat	8	175	58	100	6	48	77	15	36	0	15	41	8	18	0	7	0.62	0.62	0.76	0.40	0.20	0.63	0.63	0.80	0.35	0.23
Nusa Tenggara Timur	20	923	92	152	20	47	481	37	136	0	46	209	16	84	0	23	0.61	0.61	0.76	0.40	0.17	0.68	0.68	0.80	0.35	0.20
Kalimantan Barat	29	65	16	25	5	9	5737	318	2199	0	663	20	1	10	0	2	0.58	0.58	0.76	0.40	0.17	0.78	0.78	0.80	0.49	0.07
Kalimantan Tengah	21						48	48	48	48		0	0	0	0		0.76	0.76	0.76	0.76		0.80	0.80	0.80	0.80	
Kalimantan Selatan	8	49	49	49	49		492	98	202	58	59	0	0	0	0	0	0.47	0.47	0.76	0.40	0.16	0.80	0.80	0.80	0.80	0.00
Kalimantan Timur	21	17	9	10	7	2	3356	240	1157	0	367	271	19	166	0	50	0.48	0.48	0.76	0.40	0.15	0.77	0.77	0.80	0.35	0.12
Kalimantan Utara	11	37	12	17	5	6	7135	1189	3666	154	1258	0	0	0	0	0	0.40	0.40	0.40	0.40	0.00	0.73	0.73	0.80	0.35	0.18
Sulawesi Utara	10	663	221	410	73	172	242	31	66	0	31	157	26	58	1	26	0.52	0.52	0.76	0.40	0.19	0.50	0.50	0.80	0.35	0.23
Sulawesi Tengah	23	705	71	239	5	74	3305	167	1804	0	447	659	41	363	0	88	0.49	0.49	0.76	0.40	0.16	0.46	0.46	0.80	0.35	0.19
Sulawesi Selatan	23	435	40	90	12	23	3997	276	1757	0	482	1107	79	679	0	183	0.45	0.45	0.76	0.40	0.13	0.51	0.51	0.80	0.35	0.22
Sulawesi Tenggara	15	267	45	100	14	35	903	147	397	0	165	247	41	191	0	76	0.52	0.52	0.76	0.40	0.19	0.43	0.43	0.80	0.35	0.18
Gorontalo	4	136	45	75	21	27	227	76	140	21	60	33	11	33	0	19	0.40	0.40	0.40	0.40	0.00	0.80	0.80	0.80	0.80	0.00
Sulawesi Barat	5	321	80	208	5	90	2898	725	1276	145	469	47	12	26	0	11	0.40	0.40	0.40	0.40	0.00	0.46	0.46	0.80	0.35	0.23
Maluku	30	452	50	125	14	34	424	66	149	3	52	199	32	146	0	58	0.64	0.64	0.76	0.40	0.19	0.50	0.50	0.80	0.35	0.23
Maluku Utara	18	477	68	175	15	65	145	16	46	0	16	26	3	8	0	3	0.44	0.44	0.76	0.40	0.12	0.70	0.70	0.80	0.35	0.20
Papua Barat	28	75	25	25	25	0	2583	161	673	0	188	1144	71	259	0	95	0.48	0.48	0.76	0.40	0.15	0.66	0.66	0.80	0.35	0.21
Papua	54						12343	426	7401	0	1413	7144	246	1326	0	354	0.50	0.50	0.76	0.40	0.16	0.56	0.56	0.80	0.35	0.22

References:
ESDM One-Map https://geoportal.esdm.go.id/
World Bank - Indonesia Hydropower Study https://datacatalog.worldbank.org/dataset/indonesia-small-hydro-gis-database-2017

*) Geothermal maximum capacity factor is indifferent across the different nodes (refer to **SI-2.3** for geothermal power generation maximum CF).

Table S9. Model input of land availability and solar resource potential.

		Land available for Utility scale generation					Land available for Distributed generation					GHI, daily avg.					DNI, daily avg.				
	[n]	(sqkm)	avg.	max	min	stdev	(sqkm)	avg.	max	min	stdev	(kWh/m2)	avg.	max	min	stdev	(kWh/m2)	avg.	max	min	stdev
INDONESIA		938929					90672					4.7					2.9				
Aceh	18	17294	961	2481	0	817	2710	151	361	2	106	4.7	4.8	5.0	4.5	0.2	3.0	3.0	3.4	2.6	0.3
Sumatera Utara	19	31761	1672	4123	422	1008	5414	285	930	7	238	4.6	4.6	4.8	4.5	0.1	2.7	2.7	3.0	2.5	0.2
Sumatera Barat	14	14192	1014	3439	3	915	2482	207	598	19	186	4.6	4.7	4.9	4.4	0.2	2.7	2.8	3.2	2.4	0.2
Riau	15	67093	4473	6434	0	1747	3313	237	415	116	99	4.5	4.5	4.7	4.4	0.1	2.4	2.4	2.6	2.2	0.1
Jambi	8	31304	3913	7653	443	2047	1772	222	429	141	99	4.5	4.5	4.6	4.4	0.1	2.3	2.3	2.4	2.2	0.1
Sumatera Selatan	14	56126	4009	7534	1755	1609	4481	320	713	14	188	4.6	4.7	4.8	4.5	0.1	2.5	2.6	2.8	2.3	0.1
Bengkulu	7	7974	1139	1761	191	523	1136	189	359	101	95	4.8	4.8	4.9	4.7	0.1	3.0	3.0	3.2	2.8	0.1
Lampung	8	10236	1462	4432	491	1548	4701	672	1051	310	260	4.7	4.7	4.8	4.6	0.0	2.6	2.7	2.8	2.5	0.1
Kepulauan Bangka Belitung	6	9867	1644	2266	875	483	1043	174	267	84	78	4.5	4.5	4.6	4.4	0.0	2.6	2.7	2.8	2.5	0.1
Kepulauan Riau	11	4575	416	1523	4	461	566	71	181	0	73	4.5	4.6	4.8	4.5	0.1	2.8	2.9	3.4	2.6	0.3
DKI Jakarta	4	1	0	1	0	1	194	48	193	0	96	5.0	5.0	5.0	5.0	0.0	3.2	3.2	3.2	3.2	0.0
Jawa Barat	10	6337	634	1185	255	339	7897	790	1269	421	225	4.8	4.7	5.1	4.6	0.2	3.0	3.0	3.4	2.7	0.2
Jawa Tengah	12	4248	354	746	2	270	8583	715	1264	8	354	4.9	4.9	5.3	4.3	0.3	3.2	3.3	3.8	2.6	0.4
DI Yogyakarta	1	187	187	187	187		885	885	885	885		5.2	5.2	5.2	5.2		3.7	3.7	3.7	3.7	
Jawa Timur	17	4543	267	752	1	234	10679	628	1082	11	382	5.2	5.2	5.5	4.9	0.1	3.8	3.9	4.6	3.5	0.3
Banten	4	1540	385	754	13	337	1936	484	723	303	210	4.5	4.7	5.0	4.5	0.2	2.7	2.8	3.2	2.6	0.3
Bali	4	309	77	181	21	73	1187	297	582	49	268	4.8	4.9	5.3	4.6	0.3	3.5	3.6	4.2	3.2	0.4
Nusa Tenggara Barat	8	6657	832	1588	178	468	1424	178	424	51	145	5.4	5.3	5.5	4.9	0.2	4.5	4.4	4.9	3.8	0.4
Nusa Tenggara Timur	20	19716	986	3071	4	865	3869	193	665	1	162	5.5	5.5	5.9	5.1	0.2	4.8	4.8	5.6	4.2	0.4
Kalimantan Barat	29	86538	2984	6412	26	1315	4174	144	334	2	102	4.7	4.7	4.8	4.5	0.1	2.8	2.8	3.1	2.5	0.2
Kalimantan Tengah	21	110715	5272	9627	2145	1976	2137	107	316	3	81	4.6	4.6	4.7	4.4	0.1	2.6	2.7	2.9	2.4	0.2
Kalimantan Selatan	8	18759	2345	4734	20	1398	2415	302	671	0	236	4.6	4.6	5.0	4.5	0.2	2.9	3.0	3.9	2.7	0.4
Kalimantan Timur	21	88684	4223	7997	0	1883	2126	101	461	1	117	4.6	4.6	5.0	4.3	0.1	2.8	2.8	4.0	2.5	0.3
Kalimantan Utara	11	37028	3366	5985	1126	1826	446	41	91	1	32	4.7	4.7	4.8	4.5	0.1	2.9	2.9	3.3	2.6	0.3
Sulawesi Utara	10	5105	511	1219	1	496	960	96	216	0	86	4.9	5.0	5.2	4.8	0.1	3.7	3.9	4.2	3.5	0.2
Sulawesi Tengah	23	24335	1159	2576	2	645	2200	96	277	0	62	4.7	4.8	5.4	4.5	0.2	3.3	3.4	4.4	2.9	0.4
Sulawesi Selatan	23	10530	458	1446	0	420	4782	228	587	1	180	5.0	5.1	5.7	4.6	0.3	3.6	3.8	5.3	2.9	0.7
Sulawesi Tenggara	15	12153	810	1969	2	612	1907	127	376	2	118	4.9	4.9	5.5	4.7	0.2	3.5	3.6	4.8	3.1	0.4
Gorontalo	4	4148	1037	1397	781	284	682	171	283	38	107	5.0	5.0	5.1	4.8	0.1	3.8	3.7	3.9	3.5	0.1
Sulawesi Barat	5	4783	957	2061	22	767	904	226	265	185	33	4.8	4.8	5.0	4.7	0.1	3.3	3.3	3.5	3.1	0.2
Maluku	30	27721	956	3555	2	1072	784	29	103	2	29	4.6	4.9	5.7	4.3	0.4	3.3	3.6	5.0	2.7	0.6
Maluku Utara	18	16995	944	1913	3	714	627	35	108	1	30	4.8	4.9	5.5	4.5	0.2	3.6	3.7	4.6	3.2	0.3
Papua Barat	28	54064	2079	5750	0	1444	657	23	82	1	23	4.7	4.7	5.2	4.2	0.2	3.1	3.2	4.2	2.5	0.4
Papua	54	143413	2706	9078	2	2504	1594	30	193	1	40	4.6	4.6	5.0	4.3	0.2	2.8	2.9	3.5	2.3	0.3

References:
Global Solar Atlas https://globalsolaratlas.info/map

Table S10. Model input of wind resource potential.

		Wind-speed @100m					Wind power density @100m					CF-IEC1					CF-IEC2					CF-IEC3				
	[n]	(m/s)	avg.	max	min	stdev	(W/m2)	avg.	max	min	stdev		avg.	max	min	stdev		avg.	max	min	stdev		avg.	max	min	stdev
INDONESIA		2.9					38.0					0.04					0.05					0.06				
Aceh	18	2.5	2.9	6.5	2.0	1.2	26.8	55.4	300.5	13.6	74.8	0.02	0.05	0.30	0.01	0.08	0.03	0.07	0.35	0.01	0.09	0.04	0.08	0.38	0.02	0.10
Sumatera Utara	19	2.8	2.7	3.1	1.9	0.3	37.9	33.2	63.7	11.7	15.2	0.04	0.03	0.07	0.01	0.02	0.05	0.04	0.08	0.01	0.02	0.05	0.05	0.09	0.01	0.02
Sumatera Barat	14	2.5	2.8	4.3	2.2	0.6	27.6	38.0	115.5	16.0	29.2	0.03	0.04	0.13	0.01	0.03	0.03	0.05	0.16	0.02	0.04	0.04	0.06	0.18	0.02	0.05
Riau	15	3.1	3.1	3.7	2.5	0.3	33.1	34.4	48.1	25.2	6.6	0.03	0.03	0.05	0.02	0.01	0.04	0.05	0.07	0.03	0.01	0.05	0.05	0.08	0.04	0.01
Jambi	8	3.2	3.3	3.8	2.6	0.3	38.3	40.6	52.0	31.4	7.5	0.04	0.04	0.05	0.03	0.01	0.05	0.06	0.07	0.04	0.01	0.06	0.07	0.08	0.04	0.01
Sumatera Selatan	14	3.5	3.4	3.9	2.5	0.5	47.2	47.6	60.0	26.6	10.4	0.05	0.05	0.07	0.03	0.01	0.07	0.07	0.09	0.03	0.02	0.08	0.08	0.10	0.04	0.02
Bengkulu	7	3.0	3.1	3.9	2.7	0.4	37.0	40.0	67.1	27.7	13.5	0.04	0.04	0.08	0.03	0.02	0.05	0.05	0.10	0.04	0.02	0.06	0.06	0.11	0.04	0.02
Lampung	8	3.7	3.6	3.9	3.2	0.2	57.4	61.6	73.3	50.0	10.0	0.06	0.07	0.08	0.05	0.01	0.08	0.09	0.10	0.07	0.01	0.10	0.10	0.12	0.08	0.02
Kepulauan Bangka Belitung	6	3.9	3.9	4.0	3.8	0.1	55.0	55.0	58.3	53.4	1.7	0.06	0.06	0.07	0.06	0.00	0.08	0.08	0.09	0.08	0.00	0.09	0.10	0.10	0.09	0.00
Kepulauan Riau	11	3.8	4.0	4.5	3.5	0.3	62.0	73.9	109.1	44.3	21.5	0.07	0.08	0.12	0.05	0.03	0.09	0.11	0.15	0.06	0.03	0.10	0.12	0.17	0.07	0.03
DKI Jakarta	4	4.0	3.9	4.0	3.8	0.1	88.9	88.1	92.1	83.3	4.5	0.10	0.10	0.10	0.09	0.00	0.12	0.12	0.12	0.11	0.01	0.14	0.13	0.14	0.13	0.01
Jawa Barat	10	3.9	3.7	4.7	2.9	0.6	84.8	81.7	140.7	44.5	33.9	0.09	0.09	0.14	0.05	0.04	0.11	0.11	0.17	0.06	0.04	0.13	0.12	0.19	0.07	0.05
Jawa Tengah	12	4.0	4.2	5.2	2.9	0.8	96.8	109.1	188.2	45.1	43.1	0.10	0.12	0.20	0.05	0.05	0.13	0.14	0.24	0.06	0.06	0.15	0.16	0.26	0.07	0.06
DI Yogyakarta	1	4.8	4.8	4.8	4.8		117.6	117.6	117.6	117.6		0.14	0.14	0.14	0.14		0.17	0.17	0.17	0.17		0.20	0.20	0.20	0.20	
Jawa Timur	17	3.9	4.2	5.2	2.8	0.7	89.7	98.3	150.6	56.2	29.9	0.10	0.11	0.18	0.05	0.04	0.12	0.13	0.21	0.07	0.04	0.14	0.15	0.25	0.08	0.05
Banten	4	4.0	3.8	4.5	3.4	0.5	81.9	69.0	105.2	51.5	24.4	0.09	0.08	0.12	0.06	0.03	0.12	0.10	0.15	0.07	0.04	0.14	0.11	0.17	0.09	0.04
Bali	4	3.2	3.2	4.3	2.6	0.8	53.0	55.5	95.2	29.5	28.0	0.06	0.06	0.11	0.03	0.03	0.07	0.07	0.13	0.04	0.04	0.08	0.09	0.16	0.04	0.05
Nusa Tenggara Barat	8	3.6	3.6	4.1	3.1	0.4	72.7	73.5	99.7	58.4	14.5	0.08	0.08	0.11	0.06	0.02	0.10	0.10	0.13	0.08	0.02	0.11	0.12	0.15	0.09	0.02
Nusa Tenggara Timur	20	4.1	3.9	5.9	2.5	1.1	109.0	98.9	199.0	36.7	51.7	0.12	0.10	0.23	0.03	0.06	0.14	0.13	0.28	0.04	0.07	0.16	0.14	0.31	0.05	0.08
Kalimantan Barat	29	2.7	2.8	4.6	1.6	0.7	30.1	32.2	126.4	7.4	21.2	0.03	0.03	0.14	0.00	0.02	0.04	0.04	0.17	0.01	0.03	0.05	0.05	0.19	0.01	0.03
Kalimantan Tengah	21	2.7	2.7	4.0	1.8	0.7	26.9	28.0	56.5	12.2	14.2	0.03	0.03	0.06	0.01	0.02	0.04	0.04	0.08	0.01	0.02	0.04	0.04	0.10	0.02	0.03
Kalimantan Selatan	8	3.1	3.3	4.9	2.2	0.8	39.5	50.9	132.8	18.5	36.6	0.04	0.06	0.16	0.02	0.05	0.06	0.07	0.19	0.02	0.05	0.07	0.08	0.22	0.03	0.06
Kalimantan Timur	21	2.7	2.7	3.6	1.6	0.5	28.1	29.6	50.0	11.7	10.9	0.03	0.03	0.05	0.01	0.01	0.04	0.04	0.07	0.01	0.02	0.04	0.05	0.08	0.02	0.02
Kalimantan Utara	11	2.1	2.2	2.7	1.8	0.3	16.4	16.6	22.1	11.7	3.6	0.01	0.01	0.02	0.01	0.00	0.02	0.02	0.03	0.01	0.00	0.02	0.02	0.03	0.02	0.01
Sulawesi Utara	10	2.9	3.8	5.6	2.2	1.1	55.1	92.6	201.6	32.8	52.5	0.06	0.10	0.22	0.03	0.06	0.07	0.12	0.27	0.04	0.07	0.08	0.14	0.30	0.04	0.08
Sulawesi Tengah	23	2.5	2.5	3.9	1.7	0.6	32.8	31.8	92.8	10.6	20.1	0.03	0.03	0.10	0.01	0.02	0.04	0.04	0.12	0.01	0.03	0.05	0.05	0.13	0.01	0.03
Sulawesi Selatan	23	3.6	3.9	5.4	1.8	1.2	83.9	98.4	187.7	13.4	60.2	0.09	0.10	0.21	0.01	0.07	0.11	0.13	0.25	0.02	0.08	0.12	0.15	0.28	0.02	0.09
Sulawesi Tenggara	15	2.8	3.1	4.8	2.0	0.9	32.1	45.3	137.2	14.7	34.1	0.03	0.05	0.15	0.01	0.04	0.04	0.06	0.18	0.02	0.05	0.05	0.07	0.20	0.02	0.05
Gorontalo	4	2.9	2.9	3.8	2.2	0.7	49.8	53.3	95.4	29.3	31.0	0.05	0.05	0.10	0.03	0.03	0.06	0.07	0.12	0.03	0.04	0.07	0.08	0.14	0.04	0.05
Sulawesi Barat	5	2.0	2.5	3.5	1.7	0.7	17.1	29.4	60.7	10.7	21.1	0.01	0.03	0.07	0.01	0.03	0.02	0.04	0.09	0.01	0.03	0.02	0.04	0.10	0.01	0.04
Maluku	30	3.9	4.3	6.0	2.5	1.0	79.5	111.2	254.4	32.4	56.6	0.09	0.12	0.25	0.03	0.06	0.11	0.15	0.30	0.04	0.07	0.13	0.17	0.33	0.05	0.08
Maluku Utara	18	2.6	2.9	4.6	2.3	0.6	28.8	39.8	115.1	21.7	23.1	0.03	0.04	0.13	0.02	0.03	0.04	0.05	0.16	0.03	0.03	0.04	0.06	0.19	0.03	0.04
Papua Barat	28	2.7	2.8	3.8	2.1	0.5	29.8	32.6	74.6	12.1	16.0	0.03	0.03	0.08	0.01	0.02	0.04	0.04	0.10	0.01	0.02	0.05	0.05	0.12	0.02	0.03
Papua	54	3.0	2.8	5.6	1.6	0.9	37.1	33.0	148.1	7.9	26.9	0.04	0.03	0.18	0.01	0.03	0.05	0.04	0.23	0.01	0.04	0.06	0.05	0.27	0.01	0.05

References:
Global Wind Atlas https://globalwindatlas.info

Table S11. Model input of CO_2 storage potential.

		**CO_2 storage room**					**Maximum CO_2 injection rate**				
	[n]	($MtCO_2$)	avg.	max	min	stdev	($MtCO_2$/y)	avg.	max	min	stdev
INDONESIA		403,200					806				
Aceh	1	910	910	910	910		2	2	2	2	
Sumatera Utara	2	840	420	840	-	594	2	1	2	-	1
Riau	10	24,150	2,415	18,970	-	5,893	48	5	38	-	12
Jambi	5	115,850	23,170	77,910	-	34,732	232	46	156	-	69
Sumatera Selatan	7	37,240	5,320	20,370	-	7,505	74	11	41	-	15
Kepulauan Riau	3	2,660	887	2,310	-	1,245	5	2	5	-	2
DKI Jakarta	2	2,590	1,295	2,590	-	1,831	5	3	5	-	4
Jawa Barat	6	153,510	25,585	48,650	5,110	18,081	307	51	97	10	36
Jawa Tengah	2	70	35	70	-	49	0	0	0	-	0
Jawa Timur	7	12,530	1,790	9,450	-	3,422	25	4	19	-	7
Kalimantan Selatan	1	-	-	-	-		-	-	-	-	
Kalimantan Timur	6	48,930	8,155	48,930	-	19,976	98	16	98	-	40
Kalimantan Utara	2	1,610	805	1,610	-	1,138	3	2	3	-	2
Sulawesi Tengah	1	1	350	350	350	350		1	1	1	1
Maluku	1	1	280	280	280	280		1	1	1	1
Papua Barat	6	6	1,680	280	1,260	-	493	3	1	3	-

References:
COE CCUS Indonesia https://documents1.worldbank.org/curated/en/563781468284373788/pdf/FINAL-OK2-Jun-2415Cov-reduced-with-WB-cover.pdf

Table S12. Model input of stock electricity generation capacities.

(MW)	BP-S	BP-XS	CCGT-L	CCGT-M	CCGT-S	DPV-s4h-XS	GEOT-M	GEOT-S	GEOT-XS	HYDD-L	HYDD-M	HYDD-S	HYDR-S	HYDR-XS	ICE-S	ICE-XS	ICE-XS-BIOG
Aceh					24.0	1.1							17.8	4.4	478.1	207.4	
Sumatera Utara	90.0	38.5	817.9			0.4	429.4	10.0	2.0	783.0		219.5	113.3	7.9	535.5	51.4	14.2
Sumatera Barat						0.2	89.3			175.0	68.0	10.5	39.8	9.2		9.5	
Riau	15.0	7.9		10.0	20.0	0.3				114.0					443.0	158.3	
Jambi	20.0	3.0												0.5	213.0	30.6	
Sumatera Selatan				470.0			55.0						9.9	3.3	85.8	20.0	4.0
Bengkulu										213.0		45.6	18.0	5.5		6.0	
Lampung							220.0				90.0	28.3			46.0	12.2	3.0
Kepulauan Bangka Belitung						0.3									158.5	72.2	4.2
Kepulauan Riau		1.0		145.6											178.9	135.1	
DKI Jakarta			4,615.3												105.3	-	
Jawa Barat	14.0		1,461.2	372.0		6.2	1,193.8			1,888.7		145.0	104.4	16.2	24.8	7.7	
Jawa Tengah		19.8	1,033.9			1.8	60.0			180.9		151.3	31.1	12.2		4.4	
DI Yogyakarta														0.9			
Jawa Timur		16.7	3,043.0							105.0	54.0	131.9		3.5		63.9	
Banten			739.4			0.9							8.0	7.5	12.4	17.7	
Bali														1.4	263.7	0.7	
Nusa Tenggara Barat		1.6		126.9	11.6							18.0	7.0	9.1	386.4	130.0	
Nusa Tenggara Timur						3.4		10.0	3.9					4.0	193.6	165.2	
Kalimantan Barat	10.0	8.5				0.2					90.0			4.3	334.8	166.6	
Kalimantan Tengah	10.0	3.0													210.3	138.5	4.4
Kalimantan Selatan												30.0			69.7	48.5	2.4
Kalimantan Timur		0.8			89.6	0.1									318.3	133.1	2.0
Kalimantan Utara						1.0								0.2	161.7	83.8	
Sulawesi Utara						1.0	121.8					51.4		8.3	392.7	115.0	
Sulawesi Tengah										451.0		3.8	16.6	20.2	70.9	131.5	
Sulawesi Selatan			315.0			1.2				126.4	90.0	19.5		1.6	140.2	10.8	
Sulawesi Tenggara						0.7								5.2	209.7	203.1	
Gorontalo						2.0								6.2	21.7	1.5	
Sulawesi Barat														8.1		2.1	
Maluku						1.9									225.1	141.0	
Maluku Utara						1.0									89.9	105.5	
Papua Barat						0.2								6.5	191.1	76.8	
Papua		3.5				0.8						20.0		4.5	314.6	160.0	

Table S12. Model input of stock electricity generation capacities. (continued)

(MW)	OCGT-L	OCGT-M	OCGT-S	SBPC-L	SBPC-M	SBPC-S	UPV-s10h-M	UPV-s8h-S	USPC-L	USPC-XL	WINN-IEC2-S	WINN-IEC3-XS
Aceh					220.0							
Sumatera Utara	307.0		143.6	860.0	295.0	11.5						
Sumatera Barat			64.1		424.0	8.5						
Riau		21.6	138.2		345.0	22.0						
Jambi		161.8				20.0						
Sumatera Selatan		88.7	175.3		1,320.0	20.0						
Bengkulu		115.0			115.0							
Lampung		100.0	21.4		300.0	167.0						
Kepulauan Bangka Belitung			4.0		60.0	59.9						
Kepulauan Riau		70.0	275.4		130.0	29.0						
DKI Jakarta	400.0											
Jawa Barat	948.8	236.0	212.2		580.0	30.0			660.0	2,040.0		
Jawa Tengah		200.0	55.0	1,852.0					614.0	3,840.0		
DI Yogyakarta												
Jawa Timur	400.0	261.1	80.9	700.0		210.5			630.0	4,725.0		
Banten	400.0	200.0	8.4	915.0	295.0	120.0			1,290.0	6,075.0		
Bali		133.8	223.1	426.0								0.9
Nusa Tenggara Barat			50.0		161.1	147.0	220.0	20.0				
Nusa Tenggara Timur						78.1		5.0				
Kalimantan Barat			34.0		250.0	186.0						
Kalimantan Tengah					230.1	141.0						
Kalimantan Selatan			21.0		360.0	314.2						
Kalimantan Timur		140.0	132.0		675.0	329.1						
Kalimantan Utara			1.0			21.5						
Sulawesi Utara					120.0	110.0		18.7				0.1
Sulawesi Tengah		66.0		700.0	430.0	75.1						
Sulawesi Selatan			122.7		670.0	245.0					80.8	
Sulawesi Tenggara					187.5	70.0						
Gorontalo		100.0				136.0		11.2				
Sulawesi Barat						60.0						
Maluku												
Maluku Utara						14.0						
Papua Barat			4.0			12.0						
Papua		48.9			195.0	22.0						

References:
ESDM One-Map https://geoportal.esdm.go.id/

Table S13. Model input of stock transmission substation capacities.

	EHV					HHV					HV					MV				
(MW)	sum.	avg	max	min	stdev	sum.	avg	max	min	stdev	sum.	avg	max	min	stdev	sum.	avg	max	min	stdev
Aceh						1,150	96	200	50	50										
Sumatera Utara						3,200	229	1,250	50	314										
Sumatera Barat						1,300	186	300	50	85										
Riau						1,050	117	350	50	100										
Jambi						900	180	450	50	164										
Sumatera Selatan						2,150	195	850	50	246	175	175	175	175						
Bengkulu						300	100	150	50	50	25	25	25	25						
Lampung						1,400	233	550	100	160										
Kepulauan Bangka Belitung						400	133	250	50	104	75	75	75	75						
Kepulauan Riau						550	275	450	100	247										
DKI Jakarta	4,000	4,000	4,000	4,000		3,150	3,150	3,150	3,150		50	50	50	50						
Jawa Barat	16,000	2,667	5,000	1,000	1,633	6,000	667	1,950	100	635	1,400	156	275	50	79	1	1	1	1	
Jawa Tengah	7,000	1,750	4,000	1,000	1,500	4,000	364	1,050	50	292	25	25	25	25		2	1	1	1	-
DI Yogyakarta						450	450	450	450											
Jawa Timur	7,000	1,400	2,000	1,000	548	5,500	423	2,600	50	669	1,250	208	425	50	135	1	1	1	1	
Banten	7,000	7,000	7,000	7,000		3,400	850	1,550	50	812	50	50	50	50						
Bali						1,200	400	850	150	391										
Nusa Tenggara Barat						850	213	350	50	138	100	100	100	100						
Nusa Tenggara Timur						200	67	100	50	29	250	42	75	25	20					
Kalimantan Barat						1,200	109	250	50	70										
Kalimantan Tengah						750	94	150	50	42										
Kalimantan Selatan						950	190	350	100	108	100	100	100	100						
Kalimantan Timur						1,150	164	400	50	155										
Kalimantan Utara						150	75	100	50	35										
Sulawesi Utara						600	120	150	50	45	200	100	125	75	35					
Sulawesi Tengah						500	100	200	50	71	75	75	75	75						
Sulawesi Selatan						1,850	142	500	50	130	150	75	125	25	71	3	2	2	1	1
Sulawesi Tenggara						350	88	200	50	75	50	50	50	50						
Gorontalo						300	100	150	50	50										
Sulawesi Barat						350	88	150	50	48										
Maluku						100	100	100	100		75	75	75	75						
Maluku Utara						100	100	100	100											
Papua Barat						100	100	100	100											
Papua						50	50	50	50		100	100	100	100						

References:
ESDM One-Map https://geoportal.esdm.go.id/

Table S14. Model input of stock transmission line capacities.

**(MW-km)**		**EHV**					**HHV**					**HV**				
from	to	sum.	avg	max	min	stdev	sum.	avg	max	min	stdev	sum.	avg	max	min	stdev
Aceh	Aceh						25,610	1,829	3,621	1,303	778					
	Sumatera Utara						4,703	1,568	2,621	896	924					
Bali	Bali						4,605	1,151	1,332	971	208					
	Jawa Timur						963	963	963	963						
Banten	Banten	46,252	23,126	23,126	23,126	-	8,237	1,373	1,622	1,156	210					
	DKI Jakarta	6,505	6,505	6,505	6,505		651	651	651	651		416	416	416	416	
	Jawa Barat	31,029	15,515	18,924	12,105	4,821	2,393	1,197	1,211	1,183	20	946	946	946	946	
Bengkulu	Bengkulu						6,204	3,102	3,102	3,102	-					
	Sumatera Selatan						3,170	1,585	1,858	1,312	387					
DI Yogyakarta	Jawa Tengah	26,331	13,166	15,001	11,330	2,596	2,633	1,317	1,500	1,133	260					
DKI Jakarta	Banten	6,505	6,505	6,505	6,505		651	651	651	651		416	416	416	416	
	DKI Jakarta											905	453	453	453	-
	Jawa Barat	21,602	10,801	12,282	9,321	2,094	2,160	1,080	1,228	932	209	614	614	614	614	
Gorontalo	Gorontalo						8,202	2,050	2,451	1,650	463					
	Sulawesi Utara						1,305	1,305	1,305	1,305						
Jambi	Jambi						34,959	2,497	4,796	1,640	1,151					
	Sumatera Barat						4,160	4,160	4,160	4,160						
	Sumatera Selatan						8,691	4,345	5,995	2,696	2,333					
Jawa Barat	Banten	31,029	15,515	18,924	12,105	4,821	2,393	1,197	1,211	1,183	20	946	946	946	946	
	DKI Jakarta	21,602	10,801	12,282	9,321	2,094	2,160	1,080	1,228	932	209	614	614	614	614	
	Jawa Barat	289,730	16,096	20,688	13,215	2,439	30,843	1,542	2,069	1,185	241	9,119	760	1,034	593	151
	Jawa Tengah	25,107	12,554	16,024	9,084	4,907	2,511	1,255	1,602	908	491					
Jawa Tengah	DI Yogyakarta	26,331	13,166	15,001	11,330	2,596	2,633	1,317	1,500	1,133	260					
	Jawa Barat	25,107	12,554	16,024	9,084	4,907	2,511	1,255	1,602	908	491					
	Jawa Tengah	255,112	15,945	18,433	11,345	2,315	43,411	1,550	1,843	1,135	198					
	Jawa Timur	20,973	10,487	11,505	9,469	1,440	3,011	1,004	1,150	914	128					
Jawa Timur	Bali						963	963	963	963						
	Jawa Tengah	20,973	10,487	11,505	9,469	1,440	3,011	1,004	1,150	914	128					
	Jawa Timur	199,850	16,654	22,021	13,280	2,781	39,536	1,647	2,202	1,327	228	11,562	826	997	684	94
Kalimantan Barat	Kalimantan Barat						29,854	1,866	2,490	1,383	364					
Kalimantan Selatan	Kalimantan Selatan						18,768	1,877	2,365	1,353	458					
	Kalimantan Tengah						3,922	1,961	2,105	1,816	205					
	Kalimantan Timur						1,622	1,622	1,622	1,622						
Kalimantan Tengah	Kalimantan Selatan						3,922	1,961	2,105	1,816	205					
	Kalimantan Tengah						38,142	2,119	2,515	1,803	262					
Kalimantan Timur	Kalimantan Selatan						1,622	1,622	1,622	1,622						
	Kalimantan Timur						19,539	1,954	2,547	1,591	357					
Kepulauan Bangka Belitung	Kepulauan Bangka Belitung											944	472	472	472	-
Kepulauan Riau	Kepulauan Riau						2,746	1,373	1,373	1,373	-					
Lampung	Lampung						21,340	1,778	2,232	1,470	321					
	Sumatera Selatan						6,513	2,171	2,237	2,101	68					
Nusa Tenggara Barat	Nusa Tenggara Barat						7,786	1,298	1,700	893	361	2,433	608	648	569	45
Nusa Tenggara Timur	Nusa Tenggara Timur											6,300	788	950	662	130

Table S14. Model input of stock transmission line capacities. (continued)

**(MW-km)**		**EHV**					**HHV**					**HV**				
from	to	sum.	avg	max	min	stdev	sum.	avg	max	min	stdev	sum.	avg	max	min	stdev
Riau	Riau						35,199	1,956	2,927	1,393	426					
	Sumatera Barat						3,527	1,763	1,947	1,580	259					
	Sumatera Utara						598	598	598	598						
Sulawesi Barat	Sulawesi Barat						10,205	1,701	1,986	1,251	353					
	Sulawesi Selatan						1,652	1,652	1,652	1,652						
	Sulawesi Tengah						1,513	1,513	1,513	1,513						
Sulawesi Selatan	Sulawesi Barat						1,652	1,652	1,652	1,652						
	Sulawesi Selatan						52,157	1,739	3,586	901	802	1,183	591	591	591	-
	Sulawesi Tengah						2,642	2,642	2,642	2,642						
	Sulawesi Tenggara						2,315	2,315	2,315	2,315						
Sulawesi Tengah	Sulawesi Barat						1,513	1,513	1,513	1,513						
	Sulawesi Selatan						2,642	2,642	2,642	2,642						
	Sulawesi Tengah						11,156	1,859	2,403	1,253	517					
Sulawesi Tenggara	Sulawesi Selatan						2,315	2,315	2,315	2,315						
	Sulawesi Tenggara						11,496	1,437	1,701	1,202	243					
Sulawesi Utara	Gorontalo						1,305	1,305	1,305	1,305						
	Sulawesi Utara						9,839	1,230	1,579	1,019	243	1,019	509	509	509	-
Sumatera Barat	Jambi						4,160	4,160	4,160	4,160						
	Riau						3,527	1,763	1,947	1,580	259					
	Sumatera Barat						58,282	3,238	6,528	1,248	2,172					
	Sumatera Utara						4,766	4,766	4,766	4,766						
Sumatera Selatan	Bengkulu						3,170	1,585	1,858	1,312	387					
	Jambi						8,691	4,345	5,995	2,696	2,333					
	Lampung						6,513	2,171	2,237	2,101	68					
	Sumatera Selatan						62,255	2,830	5,051	1,540	1,299					
Sumatera Utara	Aceh						4,703	1,568	2,621	896	924					
	Riau						598	598	598	598						
	Sumatera Barat						4,766	4,766	4,766	4,766						
	Sumatera Utara						99,466	2,925	5,939	1,175	1,733	1,340	670	670	670	-

References:

ESDM One-Map https://geoportal.esdm.go.id/

Table S15. Model input of planned and under construction electricity generation capacities.

(MW)	BP-S	CCGT-L	CCGT-M	DPV-XS	GEOT-M	GEOT-S	GEOT-XS	HYDD-L	HYDD-M	HYDD-S	HYDR-S	HYDR-XS	ICE-S	ICE-XS	OCGT-L	OCGT-M	OCGT-S	SBPC-M	SBPC-S	UPV-M	UPV-S	UPV-s8h-S	USPC-L	USPC-M	USPC-XL	WINN-IEC2-S	WINN-IEC3-XS
Aceh						10			133														400				
Sumatera Utara					195			684	90				20	5				300									
Sumatera Barat					145					44																	
Riau			525										200														
Jambi					55			457															1200				
Sumatera Selatan					275	20		114															600	300	1200		
Bengkulu					220																						
Lampung					220																						
Kepulauan Bangka Belitung													21														
DKI Jakarta	35		200																								
Jawa Barat		1760			255	45		110			12.3				650					145					2244		
Jawa Tengah	5	1779		3	480	20																			3900		
Jawa Timur	9			9	275																11.1						
Banten				1	110						15.2													315	2000		
Bali				3.5	65							1.3			300												
Nusa Tenggara Barat				6								2.3	270					100	20								
Nusa Tenggara Timur				0.6	40	62	13					1.8	60					100	36								2
Kalimantan Barat								100							300			100	55								
Kalimantan Tengah													140			100			50								
Kalimantan Selatan			100												200			200	14							70	
Kalimantan Timur				1					55	29						100			14								
Kalimantan Utara									90				10	3			80		14								
Sulawesi Utara				1.3						16.6			180						106								
Sulawesi Tengah								200			19.6	4.6	40					100									
Sulawesi Selatan				1.3				464.5	90		26.3		20		200			100									
Sulawesi Tenggara		450								43			30			120											
Gorontalo											9.9								40								
Sulawesi Barat											4																
Maluku				3		20			54	12	8.8		300									15					
Maluku Utara			200			10							200	5					6		10	15					
Papua Barat	10												160						28			15					
Papua	20									24	19.2	1.5	145	5					14			20					

References:
RUPTL 2021-2030 https://gatrik.esdm.go.id/assets/uploads/download_index/files/38622-ruptl-pln-2021-2030.pdf

Table S16. Planned and under construction transmission line capacities.

**(MW-km)**		**EHV**					**HV**					**MV**				
from	to	*sum.*	*avg*	*max*	*min*	*stdev*	*sum.*	*avg*	*max*	*min*	*stdev*	*sum.*	*avg*	*max*	*min*	*stdev*
Aceh	Aceh						30,057	1,670	2,895	1,045	656					
	Sumatera Utara						1,186	1,186	1,186	1,186						
Bali	Bali	46,055	11,514	13,317	9,710	2,082	971	486	486	486	-					
	Jawa Timur	9,626	9,626	9,626	9,626											
Banten	DKI Jakarta	6,505	6,505	6,505	6,505											
	Jawa Barat						946	946	946	946						
Bengkulu	Bengkulu						7,936	992	1,551	753	349					
	Sumatera Barat						596	596	596	596						
DI Yogyakarta	Jawa Tengah						566	566	566	566						
DKI Jakarta	Banten	6,505	6,505	6,505	6,505											
	Jawa Barat	21,602	10,801	12,282	9,321	2,094										
Gorontalo	Gorontalo						1,140	570	570	570	-					
	Sulawesi Tengah						1,997	998	1,000	997	2					
	Sulawesi Utara						652	652	652	652						
Jambi	Jambi	75,246	18,812	21,218	16,405	2,779	7,755	1,292	1,837	820	458					
	Riau	17,728	17,728	17,728	17,728											
	Sumatera Barat						650	650	650	650						
	Sumatera Selatan	19,982	19,982	19,982	19,982											
Jawa Barat	Banten						946	946	946	946						
	DKI Jakarta	21,602	10,801	12,282	9,321	2,094										
	Jawa Barat	217,162	18,097	27,135	13,215	4,896	18,569	1,032	1,812	661	348					
	Jawa Tengah	16,024	16,024	16,024	16,024											
Jawa Tengah	DI Yogyakarta						566	566	566	566						
	Jawa Barat	16,024	16,024	16,024	16,024											
	Jawa Tengah	166,110	16,611	18,152	14,304	1,424	13,363	955	1,815	715	371					
	Jawa Timur	9,469	9,469	9,469	9,469											
Jawa Timur	Bali	9,626	9,626	9,626	9,626											
	Jawa Tengah	9,469	9,469	9,469	9,469											
	Jawa Timur	97,149	16,192	22,021	13,273	4,516	21,630	1,082	1,737	664	439					
Kalimantan Barat	Kalimantan Barat						24,276	867	1,105	634	137					
	Kalimantan Tengah						1,322	1,322	1,322	1,322						
Kalimantan Selatan	Kalimantan Selatan						4,645	1,161	1,178	1,144	20					
	Kalimantan Tengah						1,321	1,321	1,321	1,321						
	Kalimantan Timur						1,182	1,182	1,182	1,182						
Kalimantan Tengah	Kalimantan Barat						1,322	1,322	1,322	1,322						
	Kalimantan Selatan						1,321	1,321	1,321	1,321						
	Kalimantan Tengah						11,248	1,125	1,408	745	239					
	Kalimantan Timur						1,619	1,619	1,619	1,619						
Kalimantan Timur	Kalimantan Selatan						1,182	1,182	1,182	1,182						
	Kalimantan Tengah						1,619	1,619	1,619	1,619						
	Kalimantan Timur	189,604	23,700	25,942	18,620	3,165	24,475	1,020	1,297	639	179					
	Kalimantan Utara	19,832	19,832	19,832	19,832		992	992	992	992						

Table S16. Planned and under construction transmission line capacities. (continued)

**(MW-km)**		**EHV**					**HV**					**MV**				
from	to	*sum.*	*avg*	*max*	*min*	*stdev*	*sum.*	*avg*	*max*	*min*	*stdev*	*sum.*	*avg*	*max*	*min*	*stdev*
Kalimantan Utara	Kalimantan Timur	19,832	19,832	19,832	19,832		992	992	992	992						
	Kalimantan Utara	44,047	22,023	22,023	22,023	-	13,192	942	1,262	695	191					
Kepulauan Bangka Belitung	Sumatera Selatan						2,450	2,450	2,450	2,450						
Kepulauan Riau	Riau						992	992	992	992						
Lampung	Lampung						12,736	1,592	2,600	1,017	678					
	Sumatera Selatan						3,262	3,262	3,262	3,262						
Maluku	Maluku											53	26	26	26	-
Maluku Utara	Maluku Utara						6,614	1,653	1,754	1,553	117					
Nusa Tenggara Barat	Nusa Tenggara Barat						893	447	447	447	-					
Nusa Tenggara Timur	Nusa Tenggara Timur						4,991	832	950	686	120	130	33	37	29	5
Papua	Papua						5,724	954	1,182	814	178					
Papua Barat	Papua Barat						2,849	712	716	708	5					
Riau	Jambi	17,728	17,728	17,728	17,728											
	Kepulauan Riau						992	992	992	992						
	Riau	278,810	19,915	23,168	16,350	2,429	23,927	1,088	1,768	817	261					
	Sumatera Barat						1,947	1,947	1,947	1,947						
	Sumatera Utara	5,983	5,983	5,983	5,983		299	299	299	299						
Sulawesi Selatan	Sulawesi Selatan						21,774	1,361	1,982	901	310					
	Sulawesi Tengah						1,302	1,302	1,302	1,302						
Sulawesi Tengah	Gorontalo						1,997	998	1,000	997	2					
	Sulawesi Selatan						1,302	1,302	1,302	1,302						
	Sulawesi Tengah						23,024	886	1,275	598	228					
	Sulawesi Tenggara						1,315	1,315	1,315	1,315						
Sulawesi Tenggara	Sulawesi Tengah						1,315	1,315	1,315	1,315						
	Sulawesi Tenggara						12,273	1,227	1,701	692	377					
Sulawesi Utara	Gorontalo						652	652	652	652						
	Sulawesi Utara						4,920	615	789	509	121					
Sumatera Barat	Bengkulu						596	596	596	596						
	Jambi						650	650	650	650						
	Riau						1,947	1,947	1,947	1,947						
	Sumatera Barat						1,801	900	900	900	-					
	Sumatera Utara						824	824	824	824						
Sumatera Selatan	Jambi	19,982	19,982	19,982	19,982	#DIV/0!										
	Kepulauan Bangka Belitung						2,450	2,450	2,450	2,450						
	Lampung						3,262	3,262	3,262	3,262						
	Sumatera Selatan	123,951	20,658	23,501	16,002	3,636	18,076	1,506	2,538	800	595					
Sumatera Utara	Aceh						1,186	1,186	1,186	1,186						
	Riau	5,983	5,983	5,983	5,983	#DIV/0!	299	299	299	299						
	Sumatera Barat						824	824	824	824						
	Sumatera Utara	173,097	17,310	19,798	14,760	2,107	21,122	1,173	2,441	720	569					

References:

RUPTL 2021-2030 https://gatrik.esdm.go.id/assets/uploads/download_index/files/38622-ruptl-pln-2021-2030.pdf

Table S17. Model results of annual system costs.

(Million US$ in 2020 value)										
**scenario**	**y**	**Total**	**Gen. CAPEX**	**Gen. FOM**	**Gen. VOM**	**Gen. FUEL**	**Trans. subs.**	**Trans. line**	**CO2 transport**	**CO2 injection**
BL	2020	39.70	14.30	3.02	0.59	21.52	0.12	0.15	-	-
	2040	34.87	17.90	4.88	0.28	11.00	0.17	0.65	-	-
	2060	42.27	18.62	6.57	0.41	15.66	0.23	0.77	-	-
	2080	56.22	22.15	9.27	0.59	22.93	0.33	0.96	-	-
	2100	75.93	27.86	12.75	0.82	32.88	0.44	1.18	-	-
NZ1-MF	2020	39.71	14.30	3.02	0.59	21.53	0.13	0.15	-	-
	2040	38.67	25.06	7.36	0.22	4.84	0.19	0.72	0.03	0.25
	2060	55.44	31.22	11.45	0.63	7.56	0.25	0.94	0.14	3.24
	2080	71.66	40.93	17.22	0.68	7.34	0.36	1.24	0.18	3.71
	2100	98.65	54.62	24.09	0.98	11.13	0.51	1.74	0.25	5.33
NZ1-PF	2020	39.90	14.29	3.02	0.61	21.70	0.12	0.15	-	-
	2040	41.42	25.11	7.41	0.36	6.39	0.18	0.66	0.07	1.25
	2060	51.55	31.91	11.75	0.37	4.67	0.25	0.89	0.10	1.61
	2080	67.84	41.46	17.56	0.43	4.54	0.37	1.26	0.13	2.10
	2100	101.97	54.89	24.13	1.15	13.01	0.47	1.61	0.22	6.48
NZ2-MF	2020	39.71	14.30	3.02	0.59	21.53	0.13	0.15	-	-
	2040	37.00	22.63	6.35	0.28	6.89	0.17	0.69	-	-
	2060	48.02	30.06	11.11	0.32	4.70	0.26	0.91	0.04	0.62
	2080	66.96	41.30	17.53	0.39	4.02	0.42	1.35	0.10	1.84
	2100	93.52	54.96	24.43	0.66	7.59	0.54	1.89	0.15	3.29
NZ2-PF	2020	39.88	14.29	3.02	0.61	21.69	0.12	0.15	-	-
	2040	38.94	25.17	7.47	0.22	4.91	0.18	0.67	0.04	0.29
	2060	48.36	32.08	11.86	0.19	2.71	0.26	0.92	0.04	0.32
	2080	64.48	41.40	17.54	0.25	2.73	0.37	1.28	0.06	0.84
	2100	91.99	55.41	24.52	0.55	6.49	0.50	1.85	0.14	2.52
NZ3-MF	2020	39.71	14.30	3.02	0.59	21.53	0.13	0.15	-	-
	2040	35.55	19.81	5.15	0.31	9.43	0.17	0.69	-	-
	2060	44.58	26.40	9.35	0.33	7.42	0.23	0.84	-	-
	2080	63.29	39.99	16.81	0.30	3.88	0.37	1.29	0.02	0.62
	2100	92.90	55.10	24.48	0.62	7.08	0.53	1.88	0.18	3.02
NZ3-PF	2020	39.73	14.30	3.02	0.59	21.55	0.12	0.15	-	-
	2040	37.22	22.66	6.41	0.29	7.06	0.17	0.64	-	-
	2060	46.51	29.92	10.82	0.25	4.48	0.23	0.81	-	-
	2080	61.56	39.06	16.17	0.29	4.55	0.33	1.16	-	-
	2100	84.43	52.24	22.77	0.41	6.91	0.46	1.65	-	-
NZ4-MF	2020	39.71	14.30	3.02	0.59	21.53	0.13	0.15	-	-
	2040	36.05	21.00	5.64	0.30	8.26	0.17	0.69	-	-
	2060	44.80	26.88	9.58	0.32	6.94	0.24	0.84	-	-
	2080	60.95	37.64	15.61	0.35	5.78	0.35	1.21	-	-
	2100	86.22	52.87	23.23	0.44	6.44	0.50	1.84	0.05	0.86
NZ4-PF	2020	39.71	14.30	3.02	0.59	21.53	0.12	0.15	-	-
	2040	36.22	20.89	5.70	0.31	8.53	0.17	0.63	-	-
	2060	45.80	28.60	10.31	0.29	5.59	0.23	0.80	-	-
	2080	61.51	38.66	15.94	0.32	5.14	0.33	1.12	-	-
	2100	83.94	50.38	21.83	0.53	9.16	0.45	1.60	-	-

Table S18. Model results of capital investment.

(Million US$ in 2020 value) scenario	Y	Total	Generation	Trans. subs.	Trans. line	CO2 transport
BL	2020	18.73	18.59	0.14	-	-
	2040	112.31	102.16	0.69	9.46	-
	2060	92.36	88.92	1.00	2.44	-
	2080	135.28	130.42	1.40	3.47	-
	2100	183.69	177.74	1.74	4.20	-
NZ1-MF	2020	18.78	18.59	0.19	-	-
	2040	205.67	193.24	0.92	10.94	0.57
	2060	166.47	159.34	1.02	4.07	2.04
	2080	288.87	280.87	1.61	5.79	0.59
	2100	360.13	347.05	2.17	9.48	1.43
NZ1-PF	2020	18.70	18.56	0.14	-	-
	2040	204.53	192.78	0.81	9.72	1.23
	2060	176.19	170.21	1.16	4.30	0.52
	2080	296.92	287.40	1.74	7.16	0.62
	2100	356.06	346.11	1.56	6.70	1.69
NZ2-MF	2020	18.78	18.59	0.19	-	-
	2040	173.44	162.49	0.72	10.23	-
	2060	184.86	178.62	1.33	4.19	0.72
	2080	283.51	271.62	2.32	8.44	1.13
	2100	375.82	362.69	1.92	10.29	0.92
NZ2-PF	2020	18.70	18.56	0.14	-	-
	2040	205.88	194.57	0.86	9.80	0.66
	2060	178.07	172.01	1.20	4.78	0.08
	2080	294.52	285.47	1.70	6.97	0.38
	2100	372.78	358.61	1.90	10.79	1.48
NZ3-MF	2020	18.78	18.59	0.19	-	-
	2040	136.79	125.95	0.65	10.19	-
	2060	173.69	169.81	0.96	2.92	-
	2080	280.65	269.54	2.11	8.58	0.42
	2100	383.10	366.54	2.29	11.33	2.95
NZ3-PF	2020	18.72	18.58	0.14	-	-
	2040	172.74	162.75	0.71	9.28	-
	2060	181.14	177.00	0.84	3.30	-
	2080	262.91	254.76	1.55	6.60	-
	2100	363.66	352.44	1.90	9.33	-
NZ4-MF	2020	18.78	18.59	0.19	-	-
	2040	152.47	141.48	0.70	10.30	-
	2060	163.64	159.74	0.96	2.94	-
	2080	257.25	248.49	1.73	7.03	-
	2100	379.10	364.07	2.17	11.87	0.98
NZ4-PF	2020	18.72	18.58	0.14	-	-
	2040	150.02	140.29	0.64	9.08	-
	2060	186.53	182.41	0.90	3.22	-
	2080	253.36	245.53	1.57	6.26	-
	2100	350.62	339.84	1.78	9.00	-

Table S19. Model results of annual CO_2 emissions.

(Kton) scenario	y	CO2 net	CO2 gross	CO2 bioenergy	CO2 captured
BL	2020	160,359	170,366	10,007	-
	2040	294,490	295,516	1,025	-
	2060	402,095	402,472	377	-
	2080	589,255	589,650	394	-
	2100	844,806	845,715	909	-
NZ1-MF	2020	160,359	170,366	10,008	-
	2040	85,640	104,205	10,260	8,305
	2060	(72,696)	155,848	120,420	108,124
	2080	(116,766)	144,578	137,709	123,634
	2100	(163,360)	212,356	198,158	177,557
NZ1-PF	2020	154,074	165,079	11,005	-
	2040	50,774	139,461	47,179	41,509
	2060	(19,602)	93,944	59,933	53,614
	2080	(63,588)	84,435	78,082	69,941
	2100	(202,947)	254,648	241,565	216,030
NZ2-MF	2020	160,359	170,366	10,008	-
	2040	131,597	132,644	1,047	-
	2060	28,120	72,324	23,400	20,804
	2080	(55,307)	74,776	68,623	61,460
	2100	(95,634)	136,821	122,753	109,702
NZ2-PF	2020	154,282	165,281	10,999	-
	2040	84,199	105,485	11,707	9,579
	2060	25,681	48,283	12,065	10,537
	2080	(18,513)	40,649	31,317	27,845
	2100	(68,465)	109,974	94,337	84,102
NZ3-MF	2020	160,359	170,366	10,008	-
	2040	217,934	219,019	1,086	-
	2060	129,788	130,148	360	-
	2080	2,839	46,529	23,164	20,526
	2100	(87,279)	126,463	112,909	100,833
NZ3-PF	2020	159,016	169,386	10,370	-
	2040	130,841	131,927	1,085	-
	2060	55,522	55,937	416	-
	2080	35,253	35,688	435	-
	2100	54,116	55,042	926	-
NZ4-MF	2020	160,359	170,366	10,008	-
	2040	177,166	178,230	1,064	-
	2060	120,363	120,698	335	-
	2080	51,806	52,184	379	-
	2100	11,548	73,109	32,824	28,737
NZ4-PF	2020	159,870	169,883	10,012	-
	2040	175,910	176,988	1,078	-
	2060	78,745	79,160	415	-
	2080	39,990	40,432	442	-
	2100	72,310	73,248	937	-

Table S20. Model results of installed electricity generation capacity.

(MW)<br>scenario	y	BECCS	Bioenergy	Coal	Gas	Oil	Geothermal	Hydropower	Solar	Wind
BL	2020		8,093	35,016	19,273	18,252	2,134	5,496	292	82
	2040		1,616	53,196	35,324	2,929	4,669	8,979	13,048	154
	2060		1,320	58,306	62,724	2,852	4,546	9,867	28,127	72
	2080		1,418	83,440	90,420	3,144	3,285	10,653	39,704	
	2100		1,549	117,960	132,890	3,111	1,570	8,923	54,054	5
NZ1-MF	2020		8,092	35,016	19,273	18,249	2,134	5,496	292	82
	2040	900	1,703	40,476	19,534	3,131	5,629	12,869	162,131	154
	2060	10,350	321	13,556	14,744	521	5,571	15,440	298,396	72
	2080	11,700	413		15,600	762	4,605	16,722	512,657	
	2100	16,800	701		27,000	1,090	3,350	16,456	733,169	5
NZ1-PF	2020		8,091	35,016	19,273	18,247	2,134	5,496	292	82
	2040	3,900	1,967	40,476	17,404	3,749	5,384	11,548	155,320	154
	2060	5,100	1,179	13,556	12,694	2,522	5,586	14,660	334,982	72
	2080	6,600	751		13,990	1,551	4,600	16,042	547,852	
	2100	20,400	722		24,950	946	3,430	15,969	718,172	5
NZ2-MF	2020		8,092	35,016	19,273	18,249	2,134	5,496	292	82
	2040		1,873	40,476	37,154	3,529	5,384	12,308	120,977	154
	2060	2,100	357	13,556	31,784	604	5,616	15,237	319,034	72
	2080	5,850	375		14,150	673	4,660	16,572	548,714	
	2100	10,350	591		27,180	833	3,350	16,345	771,589	5
NZ2-PF	2020		8,091	35,016	19,273	18,247	2,134	5,496	292	82
	2040	900	2,030	40,476	19,104	3,896	5,384	11,653	169,052	154
	2060	1,650	1,137	13,556	14,984	2,426	5,586	14,770	354,205	72
	2080	2,700	844		17,360	1,768	4,600	16,161	563,253	
	2100	7,950	888		26,900	1,527	3,360	16,028	784,719	5
NZ3-MF	2020		8,092	35,016	19,273	18,249	2,134	5,496	292	82
	2040		2,286	41,676	40,164	4,491	5,274	12,010	53,872	154
	2060		1,157	15,356	50,384	2,470	5,396	15,634	241,644	72
	2080	1,950	599	600	32,970	1,195	4,605	16,944	535,730	
	2100	9,600	703		27,110	1,094	3,350	16,574	777,176	5
NZ3-PF	2020		8,091	35,016	19,273	18,247	2,134	5,496	292	82
	2040		2,260	40,476	36,904	4,432	5,219	11,481	125,631	154
	2060		1,747	13,556	35,624	3,848	5,586	14,788	321,372	72
	2080		1,427		40,420	3,127	4,490	16,317	523,197	
	2100		1,253		61,490	2,380	3,205	16,669	746,944	5
NZ4-MF	2020		8,092	35,016	19,273	18,249	2,134	5,496	292	82
	2040		2,053	41,576	41,024	3,949	5,329	12,003	82,423	154
	2060		889	14,656	47,734	1,845	5,506	15,171	252,967	72
	2080		832	1,200	50,310	1,740	4,550	16,781	494,154	
	2100	2,700	914	1,200	48,500	1,587	3,225	16,837	759,032	5
NZ4-PF	2020		8,091	35,016	19,273	18,247	2,134	5,496	292	82
	2040		2,183	40,476	40,594	4,252	5,274	10,972	86,052	154
	2060		1,738	13,556	40,174	3,828	5,586	14,466	295,568	72
	2080		1,496		44,720	3,289	4,545	16,061	513,731	
	2100		1,375		81,700	2,664	3,205	16,067	710,778	5

Table S21. Model results of annual electricity generation.

(MWh) scenario	y	BECCS	Bioenergy	Coal	Gas	Geothermal	Hydropower	Oil	Solar	Wind
BL	2020		16,537	117,779	78,775	13,525	20,745	36,387	412	162
	2040		1,080	300,480	76,163	34,650	37,811	678	18,256	225
	2060		457	415,883	134,715	33,768	43,380	409	39,429	63
	2080		657	609,276	201,264	24,393	48,237	514	55,936	
	2100		1,548	873,767	290,235	11,690	43,987	521	76,033	12
NZ1-MF	2020		16,537	117,779	78,775	13,525	20,745	36,387	412	162
	2040	6,152	1,090	86,488	39,609	41,798	61,303	703	227,549	225
	2060	80,093	314	29,833	23,360	41,401	75,975	73	417,743	63
	2080	91,582	577		13,476	34,222	82,943	107	716,075	
	2100	131,525	1,499		32,196	24,944	84,645	156	1,022,508	12
NZ1-PF	2020		17,394	108,417	87,089	13,525	20,745	36,399	412	162
	2040	30,748	1,128	86,411	35,045	39,974	53,585	790	217,981	225
	2060	39,714	434	29,833	18,845	41,512	72,242	353	468,619	63
	2080	51,808	626		12,489	34,185	79,363	220	764,860	
	2100	160,024	2,044		29,636	25,540	81,692	136	1,001,489	12
NZ2-MF	2020		16,537	117,779	78,775	13,525	20,745	36,387	412	162
	2040		1,113	112,962	81,556	39,974	58,006	759	169,946	225
	2060	15,411	318	29,833	61,179	41,736	74,822	85	446,345	63
	2080	45,526	572		11,935	34,632	82,299	94	765,972	
	2100	81,261	1,484		31,655	24,944	84,376	120	1,075,714	12
NZ2-PF	2020		17,384	108,778	86,652	13,525	20,745	36,399	412	162
	2040	7,096	1,136	86,574	38,694	39,974	54,320	810	237,232	225
	2060	7,805	428	29,833	25,463	41,512	72,674	340	495,512	63
	2080	20,626	639		21,072	34,185	79,870	251	786,449	
	2100	62,298	1,526		37,349	25,019	82,392	217	1,093,978	12
NZ3-MF	2020		16,537	117,779	78,775	13,525	20,745	36,387	412	162
	2040		1,170	206,783	87,484	39,155	55,769	894	75,495	225
	2060		432	106,391	107,240	40,097	76,281	349	338,728	63
	2080	15,205	606	1,986	55,925	34,222	83,657	173	748,530	
	2100	74,692	1,499		29,657	24,944	85,026	156	1,083,414	12
NZ3-PF	2020		16,845	115,978	80,026	13,525	20,745	36,387	412	162
	2040		1,169	110,284	85,391	38,746	52,904	886	176,401	225
	2060		514	31,142	76,547	41,360	72,808	542	449,800	63
	2080		723		96,884	33,366	80,646	447	730,651	
	2100		1,579		149,395	23,864	84,837	342	1,042,207	12
NZ4-MF	2020		16,537	117,779	78,775	13,525	20,745	36,387	412	162
	2040		1,138	166,016	85,581	39,565	56,152	818	115,511	225
	2060		394	98,357	101,631	40,917	73,561	262	354,225	63
	2080		639	8,935	122,109	33,813	82,632	250	690,838	
	2100	21,287	1,531	2,681	104,914	24,013	86,470	231	1,059,122	12
NZ4-PF	2020		16,541	117,103	79,324	13,525	20,745	36,387	412	162
	2040		1,158	160,713	93,917	39,155	49,965	860	120,819	225
	2060		513	53,059	90,649	41,360	71,059	539	413,812	63
	2080		733		109,977	33,775	79,551	470	717,776	
	2100		1,596		199,766	23,864	82,865	382	992,656	12

Table S22. Model results of installed capacity of transmission substations.

(MW) scenario	y	EHV	HHV	HV	MV
BL	2020	45,384	45,930	4,150	7
	2040	65,705	46,896	7,015	4,741
	2060	100,196	48,026	8,355	5,232
	2080	148,167	48,863	9,855	5,683
	2100	206,578	49,843	12,298	5,895
NZ1-MF	2020	46,494	46,553	5,194	7
	2040	76,610	47,863	9,490	5,199
	2060	111,763	48,007	10,771	5,397
	2080	166,348	48,214	15,814	5,510
	2100	236,985	54,306	26,038	6,108
NZ1-PF	2020	45,359	45,956	4,150	7
	2040	71,740	46,543	8,532	4,279
	2060	114,461	46,648	11,121	4,456
	2080	175,593	47,091	14,225	4,745
	2100	225,759	47,223	17,970	5,442
NZ2-MF	2020	46,494	46,553	5,194	7
	2040	68,450	47,683	9,947	4,959
	2060	110,701	53,759	24,780	5,234
	2080	185,890	67,481	51,239	5,394
	2100	250,473	67,652	52,954	6,029
NZ2-PF	2020	45,347	45,967	4,150	7
	2040	73,622	46,646	8,610	4,253
	2060	117,952	46,748	11,460	5,130
	2080	177,931	47,211	14,516	5,351
	2100	241,607	47,343	18,376	6,028
NZ3-MF	2020	46,494	46,553	5,194	7
	2040	65,440	47,537	9,998	4,822
	2060	98,633	47,732	10,988	5,679
	2080	174,755	48,063	12,787	5,999
	2100	250,873	59,500	19,222	6,615
NZ3-PF	2020	45,341	45,975	4,150	7
	2040	67,444	46,521	8,296	4,393
	2060	96,763	46,639	10,962	5,331
	2080	150,908	47,150	13,955	5,765
	2100	215,167	47,376	17,859	6,564
NZ4-MF	2020	46,494	46,553	5,194	7
	2040	67,298	47,638	9,793	5,270
	2060	100,533	47,854	10,866	5,834
	2080	160,363	48,106	12,186	6,450
	2100	236,027	48,450	14,265	7,159
NZ4-PF	2020	45,280	46,026	4,150	7
	2040	64,500	46,595	8,494	4,394
	2060	96,397	46,740	10,717	4,945
	2080	150,698	47,188	14,013	5,319
	2100	210,817	47,361	18,142	5,974

Table S23. Model results of installed capacity of transmission lines.

(MW-km) scenario	y	EHV	HHV	HV	MV
BL	2020	1,054,041	820,677	38,756	
	2040	5,847,724	872,726	682,277	467,064
	2060	7,190,871	979,105	788,128	492,135
	2080	9,243,126	1,014,883	936,922	537,215
	2100	11,789,044	1,052,025	1,143,259	549,645
NZ1-MF	2020	1,054,041	820,677	38,756	
	2040	6,658,291	875,859	845,627	490,184
	2060	9,108,004	875,859	1,100,697	505,249
	2080	12,863,354	875,859	1,334,969	512,929
	2100	18,940,308	875,859	1,615,749	548,003
NZ1-PF	2020	1,054,041	820,677	38,756	
	2040	5,993,505	826,081	833,610	437,812
	2060	8,736,030	826,081	1,061,308	449,811
	2080	13,370,969	826,081	1,368,522	468,495
	2100	17,304,503	826,081	1,770,852	506,036
NZ2-MF	2020	1,054,041	820,677	38,756	
	2040	6,141,670	856,792	871,757	479,927
	2060	8,657,989	856,792	1,111,730	502,744
	2080	14,340,330	856,792	1,353,712	513,271
	2100	21,095,788	856,792	1,608,665	549,427
NZ2-PF	2020	1,054,041	820,677	38,756	
	2040	6,027,720	826,081	852,800	436,468
	2060	9,031,047	826,081	1,114,603	466,686
	2080	13,555,250	826,081	1,414,596	483,598
	2100	20,508,344	826,081	1,801,204	519,589
NZ3-MF	2020	1,054,041	820,677	38,756	
	2040	6,025,607	856,792	934,138	479,771
	2060	7,705,076	856,792	1,116,001	525,196
	2080	13,440,699	856,792	1,327,778	546,203
	2100	20,965,269	856,792	1,581,517	578,631
NZ3-PF	2020	1,054,041	820,677	38,756	
	2040	5,710,609	826,081	791,554	442,456
	2060	7,677,043	827,883	1,001,044	477,196
	2080	11,929,727	827,883	1,256,860	501,273
	2100	17,968,320	827,883	1,579,698	557,013
NZ4-MF	2020	1,054,041	820,677	38,756	
	2040	6,123,161	856,792	882,434	494,298
	2060	7,777,681	856,792	1,101,019	529,651
	2080	12,411,711	856,792	1,270,113	565,226
	2100	20,412,239	856,792	1,482,312	599,669
NZ4-PF	2020	1,054,041	820,677	38,756	
	2040	5,469,967	826,081	868,777	444,812
	2060	7,400,724	827,883	1,074,649	470,511
	2080	11,368,484	827,883	1,357,257	492,383
	2100	17,019,412	827,883	1,792,504	536,005

Table S24. Model results of installed capacity of CO_2 transport.

(MtCO2pa-km) scenario	y	pipeL	pipeS
NZ1-MF	2040		526
	2060	7,628	1,112
	2080	9,431	1,344
	2100	11,009	2,262
NZ1-PF	2040	2,837	641
	2060	4,928	749
	2080	7,019	970
	2100	14,598	1,214
NZ2-MF	2060	1,266	446
	2080	5,448	767
	2100	7,098	1,330
NZ2-PF	2040		606
	2060		680
	2080	2,091	680
	2100	5,007	1,519
NZ3-MF	2080	2,091	33
	2100	13,498	552
NZ4-MF	2100	2,091	553

Methods S1. SELARU - MILP Formulation.

Overview

SELARU (**S**patially explicit **E**nergy and **LA**nd system Infrast**RU**cture) modelling framework is a high-resolution spatially explicit analytical tool for integrated assessments of energy and land systems using Mixed-Integer Linear Programming (MILP) optimization approach. The main feature of SELARU is its capability to address economies of scale that occur from geographical expansion of energy and land system infrastructures. This allows a more accurate system depiction to facilitate scientific investigations concerning long term infrastructure sitting/deployment in a highly complex and geographically diverse environment.

In this version, the application of SELARU is limited to Indonesia's electricity sector coupled with CCS infrastructures (**Figure S1**) to demonstrate the impact of myopic vis-à-vis perfect foresight decision-making in long-term energy system optimization. SELARU generates optimal configuration of technology applications for power generation, transmission lines and substations that minimize total system cost throughout the planning horizon. The optimization is achieved through minimizing total system costs while guaranteeing security of supply, ensuring technically feasible operation. SELARU endogenously determines the capacity expansion decisions in every 20-year timestep from 2020 to 2100.

The modelled energy system is represented as a network of interconnecting nodes. A node in the network represents a region in which energy can be utilized, converted, stored, or sent-in or -out. The lines connecting different nodes represent the connections or transmission corridors along which energy can be transported. Consequently, the basic framework of the energy system model is given by the energy balance and transfers at and from or to nodes in all times.

The required capacity deployment, generation, voltage-transformation, transmission flows, reserve capacity, and primary energy feedstock are endogenously determined by ensuring the nodal supply-demand balances are satisfied all the time. The modelled energy system is formulated using sets of equations defining the costs and technical constraints of capacity deployment and operation. The objective of optimization is to minimize the system's costs while guaranteeing security of supply, ensuring technically feasible operation, and considering region-specific resource availability and environmental restriction. Spatial-explicit information of resource, supply, and demand, as well as region-specific policy interventions are exogenously considered for each modelled timestep.

Spatial representation

SELARU uses nodes and lines to represent the spatial context of energy systems. The nodes represent geographic areas within which selection of technologies for power generation, storage, transmission lines and substations will be solved as decision variables. The nodes also contain information such as potential of renewables, energy demand, and area of exclusion zones originating from spatial aggregation within the regions that the nodes represent. The lines connecting different nodes represent eligible connections or transmission corridors along which electricity can be transported. The default mode of SELARU Indonesia application comprises 516 nodes and 1,624 lines that connect the nodes (**Figure S2**). The nodes are generated through clustering villages as the lowest administrative unit in Indonesia. This selection assumes that the village map is a suitable proximation for the geographic distribution of socio-economic activities. 83,458 villages are aggregated to 500 clusters using k-Clusters algorithm[1] performed in QGIS software. Under the algorithm, mean coordinates of villages that belong to a unique cluster—weighted using their population density and distances to neighbor villages—are used as the basis for Voronoi Tessellation[2] to generate polygons describing the clusters' bounding area. Clustered zones that include different islands or separated by water bodies are further divided, resulting in 516 nodes that represent areas ranging from 0.01-20,300 km^2, with an average of 217 km^2 which is comparable to 0.14° geographic grid resolution. International electricity trade with neighboring countries is not considered.

Nomenclature

Sets and indices

$y \in \mathbb{Y}$	year in parameterized time-step
$v \in \mathbb{Y}$	technology vintage year
$n\|n' \in \mathbb{N}$	nodes of energy supply and demand balancing
$nn' \in \mathbb{NN}$	lines of connection from node (n) to another node (n')
$i \in \mathbb{I}$	type of technology
$eg \in \mathbb{EG}$	type of electricity generation technology
$greg \in \mathbb{GREG}$	group of electricity generation technology
$ts \in \mathbb{TS}$	type of electricity transmission substation technology
$tl \in \mathbb{TL}$	type of electricity transmission line technology
$tc \in \mathbb{TC}$	type of CO_2 transport technology
$kv\|kv' \in \mathbb{KV}$	type of voltage
$f \in \mathbb{F}$	type of fuel

Parameters

yleng_y	(years) length of years of the modelled time-step (y)
$\mathrm{d}_{n,n2}$	(km) distance in km of corridor (n,n')
$\mathrm{D}_{n,y}^{\mathrm{ely}}$	(MWh) annual electricity demand at node (j) in year (y)
$\widehat{\mathrm{D}}_{n,y}^{\mathrm{ely}}$	(MW) peak electricity demand at node (j) in year (y)
typcap_i	(MW | $MtCO_2$ y^{-1}) typical capacity (size) of technology (i)
$\mathrm{life}_i^{\mathrm{tech}}$	(years) technical lifetime of technology (i)
$\mathrm{life}_i^{\mathrm{econ}}$	(years) economic lifetime of technology (i)
$\mathrm{CAPEX}_{n\|nn',i,y}$	($/MW) overnight capital costs per unit capacity of technology (i) at node (n) or corridor (nn') in year (y)
CRF_i	capital recovery factor of technology (i)
FOM_i	($/MW) fixed operating and maintenance costs per unit of capacity of technology (i)
VOM_i	($/MWh) variable operating and maintenance costs per unit of output of technology (i)
$\widehat{\mathrm{CF}}_i$	maximum capacity factor of technology (i)
$\widetilde{\mathrm{CF}}_i$	minimum capacity factor of technology (i)
η_i	rate of energy efficiency by technology (i)
η_i	rate of energy efficiency by technology (i)

[1] Hartigan, J. A. & Wong, M. A. Algorithm AS 136: A K-Means Clustering Algorithm. *J. R. Stat. Soc. Ser. C Appl. Stat.* **28**, 100–108 (1979)

[2] Boots, B., Sugihara, K., Chiu, S. N. & Okabe, A. *Spatial Tessellations: Concepts and Applications of Voronoi Diagrams*. (John Wiley & Sons, 2009)

$\mathrm{loss}_{nn',tl}$ (per 10 km) rate of energy losses of transmission line (tl) at corridor (n,n')
$\widehat{\mathrm{fmix}}_{eg,f}$ maximum share of fuel (f) in input aggregate for electricity generation (eg)
$\mathrm{landuse}_{eg}$ (km^2 MW^{-1}) area of land-use per unit of capacity of electricity generation (eg)
$\mathrm{P}_{n,f,y}$ (\$ GJ^{-1}) energy price of fuel (f) at node (n) in year (y)
$\mathrm{emsf}_f^{\mathrm{CO_2}}$ (tCO_2 GJ^{-1}) CO_2 emissions factor of fuel type (f)
$\mathrm{potential}_{n,greg}^{\mathrm{MW}}$ (MW) potential deployment capacity of electricity generation group (greg) at node (n)
GHI_n (kWh m^{-2} y^{-1}) annual global horizontal irradiance at node (n)
DNI_n (kWh m^{-2} y^{-1}) annual direct normal irradiance at node (n)
$\mathrm{CF}_{n,eg}^{\mathrm{WIND}}$ wind resource capacity factor at node (n) for electricity generation (eg)
$\mathrm{stock}_{n,eg,v,y}^{\mathbf{EG}}$ (MW) existing stock capacity of electricity generation (eg) at node (n) that was built in year (v)
$\mathrm{stock}_{n,ts,y}^{\mathbf{TS}}$ (MW) existing stock capacity of transmission substation (ts) at node (n) in year (y)
$\mathrm{stock}_{nn',tl,y}^{\mathbf{TL}}$ (MW) existing stock capacity of transmission line (tl) at corridor (n,n') in year (y)
$\mathrm{stock}_{nn',tc,y}^{\mathbf{TC}}$ ($MtCO_2$ y-1) existing stock capacity of CO_2 transport infrastructure (tc) at corridor (n,n') in year (y)
$\mathrm{prescribed}_{n,eg,v,y}^{\mathbf{EG}}$ (MW) existing stock capacity of electricity generation (eg) at node (n) that was built in year (v)
$\mathrm{prescribed}_{n,ts,y}^{\mathbf{TS}}$ (MW) existing stock capacity of transmission substation (ts) at node (n) in year (y)
$\mathrm{prescribed}_{nn',tl,y}^{\mathbf{TL}}$ (MW) existing stock capacity of transmission line (tl) at corridor (n,n') in year (y)
$\mathrm{prescribed}_{nn',tc,y}^{\mathbf{TC}}$ ($MtCO_2$ y-1) existing stock capacity of CO_2 transport infrastructure (tc) at corridor (nn') in year (y)
$\widehat{\mathrm{rate}}_n^{\mathbf{CINJECT}}$ ($MtCO_2$ y-1) maximum rate of CO_2 injection at node (n)
$\widehat{\mathrm{strorage}}_n^{\mathbf{CO2}}$ ($MtCO_2$) maximum CO_2 storage capacity at node (n)

Variables

Z (\$) cumulative total system costs
TSC_y (\$ y^{-1}) annual cost of the total system in year (y)
$TSC_{n,eg,v,y}^{\mathrm{EG}}$ (\$ y^{-1}) annual costs of electricity generation (eg) vintage year (v) at node (n) in year (y)
$TSC_{n,ts,y}^{\mathrm{TS}}$ (\$ y^{-1}) annual costs of transmission substation (sub) at node (n) in year (y)
$TSC_{nn',tl,y}^{\mathrm{TL}}$ (\$ y^{-1}) annual costs of transmission line (tl) at corridor (nn') in year (y)
$TSC_{nn',tl,y}^{\mathrm{TC}}$ (\$ y^{-1}) annual costs of CO2 transport infrastructure (tc) at corridor (nn') in year (y)

Electricity generation

$CAP_{n,eg,v,y}^{\mathbf{EG}}$ (units) installed capacity of electricity generation (eg) vintage year (v) at node (n) in year (y)
$NEW_{n,eg,v,y}^{\mathbf{EG}}$ (MW) new capacity (summarized) addition of electricity generation (eg) vintage year (v) at node (n) in year (y)
$NEWi_{n,eg,v,y}^{\mathbf{EG}}$ (unitless) new unit (integer) addition of electricity generation (eg) vintage year (v) at node (n) in year (y)
$NEWc_{n,eg,v,y}^{\mathbf{EG}}$ (MW) new capacity (continuous) addition of electricity generation (eg) vintage year (v) at node (n) in year (y)
$RET_{n,eg,v,y}^{\mathbf{EG}}$ (units) retired units of electricity generation (eg) vintage year (v) at node (n) in year (y)
$INPUT_{n,eg,v,y}$ (GJ y^{-1}) primary energy input mixture of electricity generation (eg) vintage year (v) at node (n) in year (y)
$OUTPUT_{n,eg,v,y}^{\mathbf{ely}}$ (MWh y^{-1}) electricity output of electricity generation (eg) vintage year (v) at node (n) in year (y)
$FUEL_{n,eg,v,f,y}$ (GJ y^{-1}) input fuel (f) in electricity generation (eg) vintage year (v) at node (n) in year (y)
$Cems_{n,eg,v,y}$ (tCO_2 y^{-1}) CO_2 emissions from electricity generation (eg) vintage year (v) at node (n) in year (y)
$Cbio_{n,eg,v,y}$ (tCO_2 y^{-1}) CO_2 emissions from bioenergy of electricity generation (eg) vintage year (v) at node (n) in year (y)

Transmission substation

$CAP_{n,ts,y}^{\mathbf{TS}}$ (MW) installed capacity of transformer substation (ts) at node (n) in year (y)

$NEW_{n,ts,y}^{\mathbf{TS}}$ (MW) new capacity (summarized) addition of transformer substation (ts) at node (n) in year (y)

$NEWi_{n,ts,y}^{\mathbf{TS}}$ (unitless) new unit (integer) addition of transformer substation (ts) at node (n) in year (y)

$NEWc_{n,ts,y}^{\mathbf{TS}}$ (MW) new capacity (continuous) addition of transformer substation (ts) at node (n) in year (y)

$Vup_{n,kv',kv,y}^{\mathbf{ely}}$ (MWh y^{-1}) transformation of electricity from lower (kv') to higher (kv) voltage class at node (n) in year (y)

$Vdo_{n,kv',kv,y}^{\mathbf{ely}}$ (MWh y^{-1}) transformation of electricity from higher (kv') to lower (kv) voltage class at node (n) in year (y)

$Vup_{n,kv',kv,y}^{\mathbf{plrsv}}$ (MW) transformation of planning reserve capacity from lower (kv') to higher (kv) voltage class at node (n) in year (y)

$Vdo_{n,kv',kv,y}^{\mathbf{plrsv}}$ (MW) transformation of planning reserve capacity from higher (kv') to lower (kv) voltage class at node (n) in year (y)

Transmission line

$CAP_{nn',tl,y}^{\mathbf{TL}}$ (MW) installed capacity of transmission line (tl) at corridor (nn') in year (y)

$NEW_{nn',tl,y}^{\mathbf{TL}}$ (MW) new capacity (summarized) addition of transmission line (tl) at corridor (nn') in year (y)

$NEWi_{nn',tl,y}^{\mathbf{TL}}$ (integer) new unit (integer) addition of transmission line (tl) at corridor (nn') in year (y)

$NEWc_{nn',tl,y}^{\mathbf{TL}}$ (MW) new capacity (continuous) addition of transmission line (tl) at corridor (nn') in year (y)

$FLOW_{nn',tl,y}^{\mathbf{ely}}$ (MWh y^{-1}) flow of electricity in transmission line (tl) at corridor (nn') in year (y)

$FLOW_{nn',tl,y}^{\mathbf{plrsv}}$ (MW y^{-1}) flow of planning reserve capacity in transmission line (tl) at corridor (nn') in year (y)

CO_2 capture, transport, and injection (storage)

$CCX_{n,eg,v,y}$ (tCO_2 y^{-1}) CO_2 captured from electricity generation (eg) vintage year (v) at node (n) in year (y)

$CAP_{nn',tc,y}^{\mathbf{TC}}$ (MW) installed capacity of CO_2 transport (tc) at corridor (nn') in year (y)

$NEW_{nn',tc,y}^{\mathbf{TC}}$ (MW) new capacity (summarized) addition of CO_2 transport (tc) at corridor (nn') in year (y)

$NEWi_{nn',tc,y}^{\mathbf{TC}}$ (MW) new unit (integer) addition of CO_2 transport (tc) at corridor (nn') in year (y)

$NEWc_{nn',tc,y}^{\mathbf{TC}}$ (MW) new capacity (continuous) addition of CO_2 transport (tc) at corridor (nn') in year (y)

$FLOW_{nn',tc,y}^{\mathbf{CO2}}$ (MWh y^{-1}) flow of CO_2 in CO_2 transport (tc) at corridor (nn') in year (y)

$CINJECT_{n,y}$ (tCO_2 y^{-1}) CO_2 injected at node (n) in year (y)

Objective function

The objective function of the optimization model is to minimize the cumulative total system costs (Z), which summarizes the total annual system costs ($TSC_y^{[\dots]}$) along the planning horizon ($\mathbb{Y}$) that consists of all deployed generation $\mathbb{EG}$, transmission substation $\mathbb{TS}$, transmissions $\mathbb{TL}$,. The Z is defined

$$Z = \sum_{y\in\mathbb{Y}} \left(\sum_{n\in\mathbb{N},eg\in\mathbb{EG},v} TSC^{\mathbf{EG}}_{n,eg,v,y} + \sum_{n\in\mathbb{N},ts\in\mathbb{TS}} TSC^{\mathbf{TS}}_{n,ts,y} + \sum_{nn'\in\mathbb{NN}',tl\in\mathbb{TL}} TSC^{\mathbf{TL}}_{nn',tl,y} + \sum_{nn'\in\mathbb{NN}',tc\in\mathbb{TC}} TSC^{\mathbf{TC}}_{nn',tc,y} + \sum_{n\in\mathbb{N}} TSC^{\mathbf{CINJECT}}_{n,y} \right)$$

The above formulation is set for a perfect foresight decision-making approach. However, the objective function needs to be adapted into a shorter time-horizon for a myopic decision-making approach. The optimization problem of the fully investigated time horizon is divided into multiple shorter time horizons that are solved recursively from one timestep to the next. We use loop function in GAMS to solve multiple optimizations in sequence of timesteps from start to end of planning horizon. All variables solved in previous timestep are carried over to the next timestep to ensure the balance of stock capacities by considering previously deployed capacities.

Cost equations

The TSC for each technology class comprises of costs of capacity investment annuities, operation and maintenance, and when applicable costs of input feedstock and CO_2 emissions tax penalty, given for each node n or corridor nn' and planning period y, given by

$$\forall n \in \mathbb{N}, eg \in \mathbb{EG}, y \in \mathbb{Y}, \quad TSC^{\mathbf{EG}}_{n,eg,y} = CAP_{n,eg,v,y} * \mathrm{CAPEX}_{n,eg,v} * \mathrm{CRF}_{eg} + CAP_{n,eg,v,y} * \mathrm{FOM}_{eg} + OUT^{\mathrm{ely}}_{n,eg,v,y} * \mathrm{VOM}_{eg} + \sum_{f} INPUT_{n,eg,v,f,y} * \mathrm{P}_{n,f,y} + \left(Cems_{n,eg,v,y} - Cbio_{n,eg,v,y}\right) * \mathrm{tax}^{\mathbf{Cems}}_{y}$$

$$\forall n \in \mathbb{N}, ts \in \mathbb{TS}, y \in \mathbb{Y}, \quad TSC^{\mathbf{TS}}_{n,ts,y} = CAP_{n,ts,y} * \mathrm{CAPEX}_{n,ts,y} * \mathrm{CRF}_{ts}$$

$$\forall nn' \in \mathbb{NN}', tl \in \mathbb{TL}, y \in \mathbb{Y}, \quad TSC^{\mathbf{TL}}_{nn',tl,y} = CAP_{nn',tl,y} * \mathrm{d}_{nn'} * \mathrm{CAPEX}_{nn',tl,y} * \mathrm{CRF}_{tre}$$

$$\forall nn' \in \mathbb{NN}', tc \in \mathbb{TC}, y \in \mathbb{Y}, \quad TSC^{\mathbf{TC}}_{nn',tc,y} = CAP_{nn',tc,y} * \mathrm{d}_{nn'} * \mathrm{CAPEX}_{nn',tl,y} * \mathrm{CRF}_{tre}$$

$$\forall n \in \mathbb{N}, y \in \mathbb{Y}, \quad TSC^{\mathbf{CINJECT}}_{n,y} = CINJECT_{n,y} * (\mathrm{ucost}^{\mathbf{CINJECT}}_{n,y} - \mathrm{taxcred}^{\mathbf{CINJECT}}_{y})$$

The annuity of investment for each technology computed by the quantity of deployed capacities CAP, their overnight-capital costs per unit of capacity (CAPEX), their capital recovery factor (CRF), and their length (d) of connection of line nn' or from node n to node n'. Technology-specific interest rates and economical lifetime determines the CRF for each technology. The operation and maintenance (O&M) costs are determined by the unit fixed O&M costs multiplied by installed production capacity (CAP) and by the level of production (OUT^{ely}) multiplied by the unit variable O&M costs (VOM). For fuel-firing power generation technologies, input feedstock costs are determined by the sum of all fuels f multiplied by the price of that fuel $\mathrm{P}_{n,f,y}$ at a given node n and in year y. CO_2 emissions tax-penalty accounts for the net-amount of CO_2 emissions subtracted by CO_2 neutral from the use of sustainably sourced bioenergy, multiplied by the tax on CO_2 emissions $\mathrm{tax}^{\mathrm{Cems}}_{y}$ applicable at given year y. Furthermore, costs of CO_2 injection are determined by the unit cost of injection in specific node n ($\mathrm{ucost}^{\mathbf{CINJECT}}$) minus the tax credits for CO_2 injection of a given year y ($\mathrm{taxcred}^{\mathbf{CINJECT}}$)

Electricity supply-demand matching constraints

Electricity supply-demand matching, or energy balance constraints ensure the fulfilment of electricity demand at all nodes $\mathbb{N}$ for all voltage classes $\mathbb{kv}$ in all planning periods $\mathbb{Y}$, given by

$$\forall n \in \mathbb{N}, kv \in \mathbb{kv}, y \in \mathbb{Y}, \qquad \mathrm{D}^{\mathbf{ely}}_{n,kv,y} * \left(1 + \mathrm{loss}^{\mathbf{dsub}}\right) * (1 + \mathrm{loss}^{\mathbf{dline}}) \leq S^{\mathbf{ely}}_{n,kv,y}$$

$$\forall n \in \mathbb{N}, kv \in \mathbb{kv}, y \in \mathbb{Y},$$

$$\begin{aligned} S^{\mathbf{ely}}_{n,kv,y} &= \sum_{eg \in (kv,eg), v \leq y} OUTPUT^{\mathbf{ely}}_{n,eg,v,y} + \sum_{n' \in n'n} FLOW^{\mathbf{ely}}_{n'n,kv,\,y} * \left(1 - loss_{tl \in (kv,tl)} * \mathrm{d}_{n'n}\right) \\ &- \sum_{n' \in nn'} FLOW^{\mathbf{ely}}_{nn',kv,\,y} + \sum_{kv' \leq kv} Vup^{\mathbf{ely}}_{n,kv',kv,y} + \sum_{kv' \geq kv} Vdo^{\mathbf{ely}}_{n,kv',kv,y} * \left(1 - loss_{ts \in (kv,ts)}\right) \\ &- \sum_{kv' \geq kv} Vup^{\mathbf{ely}}_{n,kv,kv',y} - \sum_{kv' \leq kv} Vdo^{\mathbf{ely}}_{n,kv,kv',y} * \left(1 - loss_{ts \in (kv,ts)}\right) \end{aligned}$$

Electricity demand at specific node n, voltage class kv, and planning period y ($\mathrm{D}^{\mathbf{ely}}$) is supplied ($S^{\mathbf{ely}}$) with the power generated $OUT^{\mathbf{ely}}_{eg \in (kv,eg)}$, plus all incoming transmission from all other nodes n', minus all outgoing transmission to all other nodes n' ($FLOW^{\mathbf{ely}}$), and plus net-change of voltage classes step-up $Vup^{\mathbf{ely}}$ and step-down $Vdo^{\mathbf{ely}}$ to and from other voltage classes kv' with considering transformation and transmissions losses ($loss_{ts}, loss_{tl}$).

Planning reserve supply-demand matching constraints

Planning reserves is required for the system to have sufficient firm capacity to meet the forecasted demand peak load plus a reserve margin, given by

$$\forall n \in \mathbb{N}, kv \in \mathbb{kv}, y \in \mathbb{Y},$$

$$\begin{aligned} \widehat{\mathrm{D}}^{\mathrm{ely}}_{n,kv,y} * \left(1 + \mathrm{margin}^{\mathbf{plrsv}}_{n}\right) &= \sum_{eg \in (kv,eg), v \leq y} CAP^{\mathrm{plrsv}}_{n,eg,v,y} + \sum_{n' \in n'n} FLOW^{\mathbf{plrsv}}_{n'n,tl,y} * \left(1 - loss_{tl \in (kv,tl)} * \mathrm{d}_{n'n}\right) \\ &- \sum_{n' \in nn'} FLOW^{\mathbf{plrsv}}_{nn',tl,y} + \sum_{kv' \leq kv} Vup^{\mathbf{plrsv}}_{n,kv',kv,y} + \sum_{kv' \geq kv} Vdo^{\mathbf{plrsv}}_{n,kv',kv,y} * \left(1 - loss_{ts \in (kv,ts)}\right) \\ &- \sum_{kv' \geq kv \in (kv,ts)} Vup^{\mathbf{plrsv}}_{n,kv,kv',y} - \sum_{kv' \leq kv} Vdo^{\mathbf{plrsv}}_{n,kv,kv',y} * \left(1 - loss_{ts \in (kv,ts)}\right) \end{aligned}$$

With $CAP^{\mathbf{plrsv}}_{e} \leq CAP_{n,eg,v,y} * \mathrm{capcredit}^{\mathbf{plrsv}}_{eg}$

The supplied reserve capacities must exceed the peak demand ($\widehat{\mathrm{D}}^{\mathbf{ely}}_{n,kv,y}$) plus a margin for planning reserve ($\mathrm{margin}^{\mathbf{plrsv}}$) of respective supply-demand balancing region n. $\widehat{\mathrm{D}}^{\mathbf{ely}}_{n,kv,y}$ is approximated using load factors of annual demand. Reserve capacities ($CAP^{\mathbf{plrsv}}_{n,eg,v,y}$) are supplied by all generation capacity capped by technology specific capacity reserve credits ($\mathrm{capcredit}^{\mathbf{plrsv}}_{eg}$), plus net incoming-outgoing flows of planning reserve capacities ($FLOW^{\mathbf{plrsv}}$), plus net-change of voltage class step-up and -down of reserve capacities ($Vup^{\mathbf{plrsv}}, Vdo^{\mathbf{plrsv}}$), with also taking into account the losses incurred at transmission substation and transmission line. Each technology is assigned with capacity credit. Reflecting on its expected availability when power is needed. For instance, conventional power generating technologies have their maximum availability committed for planning reserve capacity provision.

Electricity transmission and voltage transformation constraints

Transmissions of electricity ($FLOW^{\mathbf{ely}}$) are capped by built transmission line capacities (CAP), or maximum transfer limit, given by

$\forall nn' \in \mathbb{NN}', tl \in \mathbb{TL}, kv(kv, tl), y \in \mathbb{Y},$

$$CAP^{\mathbf{TL}}_{nn',tl\in(kv,tl),y} * 8760 \geq FLOW^{\mathbf{ely}}_{nn',kv,\,y}$$

$$CAP^{\mathbf{TL}}_{nn',tl\in(kv,tl),y} \geq FLOW^{\mathbf{plrsv}}_{nn',kv,y}$$

Transformer substation maximum capacity limit determines the maximum voltage transformation and reserves for extra high voltage (EHV) and high voltage (HV) transmission. However, for medium voltage (MV), substation capacity must cover all incoming and outgoing transmission lines' capacity. This ensures a must built MV substation for each MV line-connected neighboring nodes. Detailed design and analysis of lower voltage (LV) transmissions or distribution network are out of the scope of study. The constraints governing how voltage transformation flows and capacities interact are given by,

$\forall n \in \mathbb{N}, ts \in \mathbb{TS}, kv \in (kv, ts), y \in \mathbb{Y},$

$$CAP^{\mathbf{TS}}_{n,ts\in(kv,ts),y} * 8760 \geq \sum_{kv>kv'} Vup^{\mathbf{ely}}_{n,kv',kv,y}$$

$$CAP^{\mathbf{TS}}_{n,ts\in(kv,ts),y} * 8760 \geq \sum_{kv>kv'} Vdo^{\mathbf{ely}}_{n,kv,kv',y}$$

$$CAP^{\mathbf{TS}}_{n,ts\in(kv,ts),y} \geq \sum_{kv>kv'} Vup^{\mathbf{plrsv}}_{n,kv',kv,y}$$

$$CAP^{\mathbf{TS}}_{n,ts\in(kv,ts),y} \geq \sum_{kv>kv'} Vdo^{\mathbf{plrsv}}_{n,kv,kv',y}$$

$$\forall \ldots kv \in \{\text{MV}\}, \qquad CAP^{\mathbf{TS}}_{n,ts\in(kv,ts),y} \geq \sum_{n'} CAP^{\mathbf{TL}}_{nn',tl\in(kv,tl),y} + \sum_{e'} CAP^{\mathbf{TL}}_{n'n,trl\in(kv,tl),y}$$

Electricity generation constraints

For all power generating technologies $\mathbb{EG}$ the input-output balance of energy conversion and maximum or minimum generation limits are given by,

$\forall n \in \mathbb{N}, eg \in \mathbb{EG}, v, y \in \mathbb{Y},$

$$INPUT_{n,eg,v,y} * \eta_{eg} = OUTPUT^{\mathbf{ely}}_{n,eg,v,y}$$

$$CAP^{\mathbf{EG}}_{n,eg,v,y} * \widehat{\mathrm{CF}}_{eg} * 8760 \geq OUTPUT^{\mathbf{ely}}_{n,eg,v,y}$$

$$CAP^{\mathbf{EG}}_{n,eg,v,y} * \widetilde{\mathrm{CF}}_{eg} * 8760 \leq OUTPUT^{\mathbf{ely}}_{n,eg,v,y}$$

The output electricity generated ($OUTPUT^{ely}$) are influenced by the amount of energy input ($INPUT$) and the energy conversion efficiency (η) of specific electricity generation technology eg. Electricity generation is capped by $\widehat{\mathrm{CF}}$ as a factor of annual production of the installed generation capacity ($CAP^{\mathbf{EG}}$). For generation capacities that provide planning reserve capacities, $\widetilde{\mathrm{CF}}$ determines the minimum production threshold.

The input fuel feedstock constraints are given by,

$\forall n \in \mathbb{N}, eg \in \mathbb{EG}, v, y \in \mathbb{Y},$

$$INPUT_{n,eg,v,y} = \sum_{f} FUEL_{n,eg,v,f,y}$$

$$INPUT_{n,eg,v,y} * \widehat{\mathrm{fmix}}_{eg,f} \geq FUEL_{n,eg,v,f,y}$$

The amount of energy input ($INPUT$) comprises of single or mixture of fuels f ($FUEL$) that are specific for each type of electricity generation technology (eg). $\widehat{\mathrm{fmix}}$ regulates the share of each fuel f in the mixture.

Plant CO_2 emissions are given by,

$\forall n \in \mathbb{N}, eg \in \mathbb{EG}, v, y \in \mathbb{Y},$

$$Cems_{n,eg,v,y} = \sum_{f} INPUT_{n.eg,v,f,y} * \mathrm{emsf}^{\mathrm{CO_2}}_{f}$$

$$Cbio_{n,eg,v,y} = \sum_{f \in bioenergy} INPUT_{n.eg,v,f,y} * \mathrm{emsf}^{\mathrm{CO_2}}_{f} * \mathrm{neutral}^{\mathrm{CO_2}}_{f}$$

$$CCX_{n,eg,v,y} = Cems_{n,eg,v,y} * \mathrm{rateCCX}_{eg}$$

Plant annual CO_2 emissions ($Cems$) are accounted based on CO_2 emissions factor of fuels ($\mathrm{emsf}^{\boldsymbol{CO_2}}_{f}$) used and the respective consumption ($INPUT$). Note that biomass-based fuels are considered with a degree of emissions neutrality ($\mathrm{neutral}^{\mathrm{CO_2}}_{f}$). Furthermore, plant annual CO_2 capture is estimated by technology-specific CO_2 capture rate ($\mathrm{rateCCX}_{eg}$).

Resource constraints

Maximum potential-built capacity and availability of RES power generation $\mathbb{EG}^{\mathrm{RES}}$ at given location n is limited by the potential built capacity of RES group ($\mathrm{potential}^{\mathrm{RES}}$), given by

$$\forall n \in \mathbb{N}, y \in \mathbb{Y}, \qquad \sum_{eg \in \mathrm{HYDRO|GEOT}, v \le y} CAP^{\mathbf{EG}}_{n,eg,v,y} \le \mathrm{potential}^{\mathrm{HYDRO|GEOT}}_{n}$$

$$\forall n \in \mathbb{N}, y \in \mathbb{Y}, \qquad \sum_{eg \in \mathrm{SOLAR|WIND}, v \le y} CAP^{\mathbf{EG}}_{n,eg,v,y} * \mathrm{landuse}_{eg} \le \mathrm{landavail}_{n}$$

The potential RES capacities in MW are estimated to use a factor of capacity-land density for hydropower and geothermal power. Meanwhile for solar and wind power, potential deployable capacities are limited by land availability ($\mathrm{landavail}$) in each node n. The land availability excludes urban developed areas, water bodies, and protected areas.

Solar energy input is calculated based on by the size of irradiated-surface area multiplied by the average of annual Global Horizontal Irradiance (GHI) for PV $\mathbb{EG}^{\mathrm{PV}}$, or Direct Normal Irradiance (DNI) measured in kWh/m^2 for CSP $\mathbb{EG}^{\mathrm{CSP}}$.The availability of solar resource is given by,

$$\forall n \in \mathbb{N}, eg \in \mathbb{EG}^{\mathbf{UPV}}, v, y \in \mathbb{Y}, \qquad INPUT_{n,eg,v,y} \le CAP^{\mathbf{EG}}_{n,eg,v,y} * \mathrm{surface}^{\mathrm{PV}}_{eg} * \mathrm{GHI}^{\mathrm{UPV}}_{n} * 365.25$$

$$\forall n \in \mathbb{N}, eg \in \mathbb{EG}^{\mathbf{DPV}}, v, y \in \mathbb{Y}, \qquad INPUT_{n,eg,v,y} \le CAP^{\mathbf{EG}}_{n,eg,v,y} * \mathrm{surface}^{\mathrm{PV}}_{eg} * \mathrm{GHI}^{\mathrm{DPV}}_{n} * 365.25$$

$$\forall n \in \mathbb{N}, eg \in \mathbb{EG}^{\mathbf{CSP}}, v, y \in \mathbb{Y}, \qquad INPUT_{n,eg,v,y} \le CAP^{\mathbf{EG}}_{n,eg,v,y} * \mathrm{surface}^{\mathrm{CSP}}_{eg} * \mathrm{DNI}_{n} * 365.25$$

For wind power electricity generation $\mathbb{EG}^{\mathbf{WIND}}$, annual wind electricity generation are capped by the capacity factor of wind resource ($\mathrm{CF}^{\mathbf{WIND}}_{n,eg}$) that are geographically distributed and at different levels depending on the class of wind turbine technology suited for in situ wind speed and turbine heights. The classification of wind turbine technology refers to IEC 61400, the International Standard published by the International Electrotechnical Commission regarding wind turbines. The maximum production of wind electricity generation is given by,

$$\forall n \in \mathbb{N}, eg \in \mathbb{EG}^{\mathbf{WIND}}, v, y \in \mathbb{Y}, \qquad OUTPUT^{\mathbf{ely}}_{n,eg,v,y} \le 8760 * CAP^{\mathbf{EG}}_{n,eg,v,y} * \mathrm{CF}^{\mathbf{WIND}}_{n,eg}$$

For hydropower, annual electricity generation are capped by maximum capacity factors that are site specific. The maximum production of hydropower electricity generation is given by,

$$\forall n \in \mathbb{N}, eg \in \mathbb{EG}^{\mathbf{HYDD\,|\,HYDR}}, v, y \in \mathbb{Y}, \qquad OUTPUT^{\mathbf{ely}}_{n,eg,v,y} \le 8760 * CAP^{\mathbf{EG}}_{n,eg,v,y} * \mathrm{CF}^{\mathbf{HYDD\,|\,HYDR}}_{n,eg}$$

Capacity balance constraints

Capacity balance constraints ensure the planned capacity deployment with considering previously built, retired, and newly added capacities given by

$\forall n \in \mathbb{N}, eg \in \mathbb{EG}, v, y \in \mathbb{Y},$

$$CAP^{\mathrm{EG}}_{n,eg,v,y} = CAP^{\mathbf{EG}}_{n,eg,v,y-1} + NEW^{\mathbf{EG}}_{n,eg,v=y} - RET^{\mathbf{EG}}_{n,eg,v,y} + \mathrm{Stock}^{\mathbf{EG}}_{n,eg,v}[\mathrm{start}(y)], \forall n \in \mathbb{N}, eg \in \mathbb{EG}, v \leq y, y \in \mathbb{Y}$$

$\forall n \in \mathbb{N}, eg \in \mathbb{EG}, v,$

$$NEW^{\mathbf{EG}}_{n,eg,v} = \left(NEWi^{\mathbf{EG}}_{n,eg,v} * \mathrm{typcap}^{\mathbf{EG}}_{eg}\right) + NEWc^{\mathbf{EG}}_{n,eg,v} + \mathrm{Prescribed}^{\mathbf{EG}}_{n,eg,y},$$

with $NEWi^{\mathbf{EG}}_{n,eg,v} \in \mathbb{Z} \geq 0$, and $NEWc^{\mathbf{EG}}_{n,eg,v} \in \mathbb{R} \geq 0$

$\forall n \in \mathbb{N}, eg \in \mathbb{EG}, v, y \in \mathbb{Y},$

$$RET^{\mathbf{EG}}_{n,eg,v,y} = \sum\nolimits_{y-v \leq \mathrm{life}_{eg}} CAP^{\mathbf{EG}}_{n,eg,v,y-1}$$

In all locations $\mathbb{N}$, deployed generation capacity (CAP) of technology type eg in current period y includes the deployed capacities in previous period ($y-1$), minus capacities that exceed their economic lifetime ($y - v \leq \mathrm{life}_{eg}$), and plus new capacity additions (NEW). New capacity additions are defined in both integers ($NEWi \in \mathbb{Z}$) and real numbers ($NEWc \in \mathbb{R}$), respective to larger or smaller capacity numeration. Stock capacities (Stock) are considered in the initial period of the planning horizon. Moreover, prescribed capacities ($\mathrm{Prescribed}$) are also considered in new capacity additions.

Electricity transmission substation (ts), transmission line (tl), and CO_2 transport (tc) capacities are assumed to have no retirement and the only way is to expand, given by

$\forall n \in \mathbb{N}, ts \in \mathbb{TS}, y \in \mathbb{Y}, \quad CAP^{\mathbf{TS}}_{n,ts,y} = CAP^{\mathbf{TS}}_{n,ts,y-1} + NEW^{\mathbf{TS}}_{n,ts,v=y} + \mathrm{Stock}^{\mathbf{TS}}_{n,ts,v=y}[\mathrm{start}(y)]$

$\forall n \in \mathbb{N}, tl \in \mathbb{TL}, y \in \mathbb{Y}, \quad CAP^{\mathbf{TL}}_{nn',tl,y} = CAP^{\mathbf{TL}}_{nn',tl,y-1} + NEW^{\mathbf{TL}}_{nn',tl,v=y} + \mathrm{Stock}^{\mathbf{TL}}_{nn',tl,v=y}[\mathrm{start}(y)]$

$\forall n \in \mathbb{N}, tc \in \mathbb{TC}, y \in \mathbb{Y}, \quad CAP^{\mathbf{TC}}_{nn',tc,y} = CAP^{\mathbf{TC}}_{nn',tc,y-1} + NEW^{\mathbf{TC}}_{nn',tc,v=y} + \mathrm{Stock}^{\mathbf{TC}}_{nn',tc,v=y}[\mathrm{start}(y)]$

CO_2 transport and storage constraints

CO_2 source-sink matching ensures that all captured CO_2 (CCX) are either injected in the same node n ($CINJECT$) or transported to and from another node n' ($FLOW^{\mathbf{CO2}}$), given by

$$\forall n \in \mathbb{N}, y \in \mathbb{Y}, \qquad CINJECT_{n,y} = \sum_{eg \in \mathrm{CCX}(eg), v \le y} CCX_{n,eg,v,y} + \sum_{n' \in n'n, tc \in \mathbb{TC}} FLOW^{\mathbf{CO2}}_{n'n,tc,y} - \sum_{n' \in nn', tc \in \mathbb{TC}} FLOW^{\mathbf{CO2}}_{nn',tc,y}$$

CO_2 transport flow ($FLOW^{\mathbf{CO2}}$) is capped by built CO_2 transport capacity ($CAP^{\mathbf{TC}}$), or maximum transfer limit, given by

$$\forall n \in \mathbb{N}, tc \in \mathbb{TC}, y \in \mathbb{Y}, \ CAP^{\mathbf{TC}}_{nn',tc,y} \ge FLOW^{\mathbf{CO2}}_{nn',tc,y}$$

CO_2 injection and storage are capped by the annual maximum rate of CO2 injection ($\widehat{\mathrm{rate}}^{\mathbf{CINJECT}}$) and the CO_2 storage availability ($\widehat{\mathrm{storage}}^{\mathbf{CO2}}$), given by

$$\forall n \in \mathbb{N}, y \in \mathbb{Y}, \qquad CINJECT_{n,y} \le \widehat{\mathrm{rate}}^{\mathbf{CINJECT}}_n$$

$$\forall n \in \mathbb{N}, \qquad \sum_{y \in \mathbb{Y}} CINJECT_{n,y} \le \widehat{\mathrm{storage}}^{\mathbf{CO2}}_n$$

Climate policy constraints

Total system's long-term cumulative net CO_2 emissions cannot go over the long-term quota of CO_2 emissions ($LimitPF^{Cems}_y$). Scenarios that do not consider climate policy can put a very large number to virtually set without limits. Net CO_2 emissions are derived by subtracting CO_2 neutral ($Cbio$) and CO_2 captured (CCX) from gross CO_2 emissions ($Cems$).

$$LimitPF^{Cems} \ge \sum_{n,eg,v,y} Cems_{n,eg,v,y} - \sum_{n,eg,v,y} Cbio_{n,eg,v,y} - \sum_{n,eg,v,y} CCX_{n,eg,v,y}$$

The above formulation is suited under the perfect foresight decision-making approach. However, in myopic approach, decisions do not consider long-term implications. Therefore, constraint on system's CO_2 emissions is set for each timestep. For myopic target setting, the total system's annual net CO_2 emissions cannot go over the annual limit on CO_2 emissions ($LimitMF^{Cems}_y$).

$$LimitMF^{Cems}_y \ge \sum_{n,eg,v} Cems_{n,eg,v,y} - \sum_{n,eg,v} Cbio_{n,eg,v,y} - \sum_{n,eg,v,y} CCX_{n,eg,v,y}$$
